# Oral ginger-derived extracellular vesicles ameliorate arthritis via anti-inflammatory actions of microRNA-149 and 6-gingerol

**DOI:** 10.1016/j.omtn.2026.102840

**Published:** 2026-01-19

**Authors:** Hiroki Kaneta, Tomoyuki Nakasa, Dilimulati Yimiti, Dan Moriwaki, Riku Kawasaki, Toshihiko Ogura, Shigeru Miyaki, Nobuo Adachi

**Affiliations:** 1 Department of Orthopaedic Surgery, Graduate School of Biomedical and Health Sciences, Hiroshima University, 1-2-3 Kasumi, Minami-ku, Hiroshima 734-8551, Japan; 2 Department of Artificial Joints and Biomaterials, Graduate School of Biomedical and Health Sciences, Hiroshima University, 1-2-3 Kasumi, Minami-ku, Hiroshima 734-8551, Japan; 3 Program of Applied Chemistry, Graduate School of Advanced Science and Engineering, 1-4-1 Kagamiyama, Higashi-Hiroshima, Japan; 4 National Institute of Advanced Industrial Science and Technology (AIST), Central 6, Higashi, Tsukuba, Ibaraki 305-8566, Japan; 5 Medical Center for Translational and Clinical Research, Hiroshima University Hospital, 1-2-3 Kasumi, Minami-ku, Hiroshima 734-8551, Japan

**Keywords:** MT: Delivery Strategies, ginger-derived extracellular vesicles, rheumatoid arthritis, anti-inflammation, synovial fibroblasts, miR-149, oral arthritis therapy

## Abstract

Ginger-derived extracellular vesicles (GDEVs) have emerged as a novel anti-inflammatory agent with advantages such as oral bioavailability, natural origin, and cost-effective large-scale production. This study evaluated the therapeutic potential of GDEVs in rheumatoid arthritis (RA), a chronic autoimmune disease characterized by synovial inflammation and joint destruction. We conducted both *in vitro* and *in vivo* experiments using synovial fibroblasts derived from RA patients and a collagen antibody-induced arthritis (CAIA) mouse model. *In vitro*, GDEVs significantly suppressed the expression of pro-inflammatory cytokines tumor necrosis factor-α (TNF-α) and interleukin (IL)-1β and downstream mediators IL-6, Cox-2, and matrix metalloproteinase 3 (MMP3) and inhibited the proliferation and migration of RA synovial fibroblasts. *In vivo*, oral administration of GDEVs to CAIA mice reduced arthritis severity, attenuated synovitis, preserved cartilage integrity, and suppressed osteoclast activation. GDEVs were stable against gastric digestion and were efficiently taken up by intestinal cells, supporting their oral availability. Microarray and RNA sequencing identified miR-149 as a key regulatory molecule in GDEVs, associated with the suppression of inflammation-related signaling pathways, including Ras signaling and mitogen-activated protein kinase (MAPK) cascades. These findings highlight the potential of GDEVs as an anti-inflammatory therapy for RA. Given their stability and bioavailability, the oral administration of GDEVs could be a promising non-invasive treatment for future clinical applications.

## Graphical abstract

**Figure:**
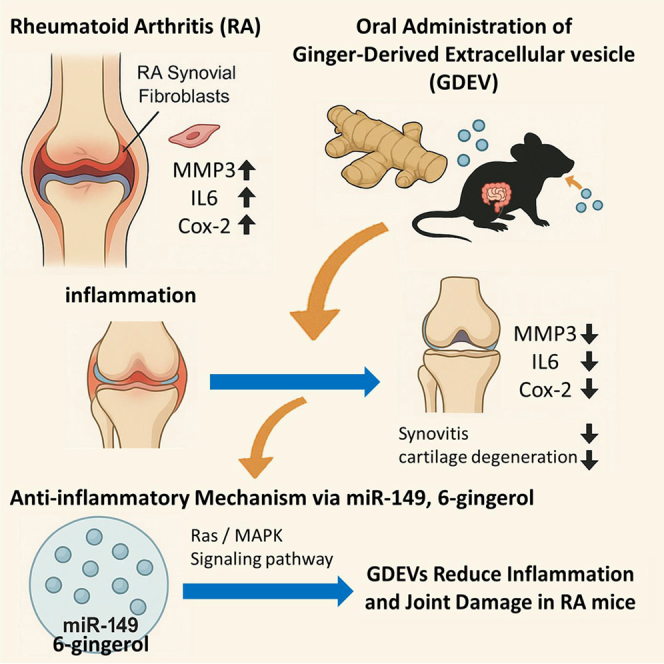

## Teaser

Nakasa and colleagues show that orally administered ginger-derived extracellular vesicles (GDEVs), enriched with miR-149 and 6-gingerol, alleviate rheumatoid arthritis by modulating inflammatory pathways. Their study highlights a novel, plant-based, non-invasive therapeutic strategy leveraging the bioactive and immunomodulatory properties of GDEVs with potential for clinical translation.

## Introduction

Rheumatoid arthritis (RA) is a chronic autoimmune disease characterized by persistent synovial inflammation leading to joint destruction and functional impairment.^1^^,^^2^ The primary pathological features of RA include hyperplasia of synovial fibroblasts, excessive production of proinflammatory cytokines, and recruitment of immune cells, all of which contribute to cartilage degradation and bone erosion.^3^ Uncontrolled RA can result in musculoskeletal deformities, impaired functionality, and high morbidity rates. Additionally, RA is associated with various extra-articular manifestations, including cardiovascular complications, due to its chronic systemic inflammatory and autoimmune nature.^4^^,^^5^ Modern therapeutic strategies for managing RA include the use of nonsteroidal anti-inflammatory drugs (NSAIDs), disease-modifying antirheumatic drugs (DMARDs), glucocorticoids, biologics, and Janus kinase inhibitors (JAKs). Although these treatments effectively reduce the symptoms and decelerate disease progression, they have notable limitations.^6^ These therapies are often associated with high costs and adverse effects, such as immunosuppression and increased infection risk, and require frequent administration, either through injections or prolonged use, which can lead to poor patient compliance and an increased risk of systemic complications.^7^^,^^8^ Therefore, the development of an effective and safe oral therapeutic strategy remains an urgent challenge in the management of RA.

Extracellular vesicles (EVs) have emerged as potential therapeutic agents because of their ability to mediate intercellular communication by delivering bioactive molecules, such as microRNAs (miRNAs) and messenger RNAs (mRNAs).^9^ Recent studies have highlighted the therapeutic potential of EVs in modulating immune responses and promoting tissue repair.^10^^,^^11^ However, the clinical application of EV therapy is hindered by challenges, such as high production costs, stability issues, and delivery inefficiencies.^12^ To overcome these limitations, plant-derived EVs (PDEVs) have been investigated as promising alternatives that offer advantages in terms of their stability, scalability, and cost-effectiveness.^13^ Several studies have shown that PDEVs are taken up by mammalian cells and exert immunomodulatory effects, indicating their potential role in inflammatory disease treatment.^14^^,^^15^ Furthermore, PDEVs have been shown to carry various bioactive molecules, including miRNAs, lipids, and metabolites, which contribute to their immunomodulatory and therapeutic effects in diverse diseases (e.g., colitis, liver injury, and cancer).^16^ These components can modulate inflammatory signaling pathways, enhance tissue repair, and influence immune cell functions, as summarized in Table S1.

Ginger (*Zingiber officinale*) is a widely used medicinal plant known for its potent anti-inflammatory properties.^17^^,^^18^ Ginger and its bioactive components have been reported to alleviate RA-related symptoms and may contribute to the suppression of disease progression in preclinical models.^19^^,^^20^ 6-Gingerol, a phenolic compound, has been shown to inhibit the production of inflammatory mediators such as tumor necrosis factor-α (TNF-α) and interleukin (IL)-1β by suppressing 5-lipoxygenase and prostaglandin synthase activity.^21^ Meanwhile, 6-shogaol has demonstrated the ability to inhibit the proliferation and migration of RA synovial fibroblasts (RASFs) via modulation of the phosphoinositide 3-kinase/protein kinase B and nuclear factor kappa-light-chain-enhancer of activated B cells (NF-κB) signaling pathways.^22^ Both compounds also reduce the expression of matrix metalloproteinases (MMPs), which are key contributors to cartilage degradation.^22^^,^^23^^,^^24^ Their effects have been validated in preclinical models of arthritis, including collagen- and carrageenan-induced arthritis, where they significantly reduced joint swelling, synovial inflammation, and histological damage.^22^^,^^25^ However, despite these promising findings, the direct application of ginger-derived bioactive compounds such as 6-gingerol faces several challenges. These compounds have limited bioavailability due to their rapid metabolism and poor solubility, which may reduce their therapeutic efficacy when administered orally or systemically.^18^ In addition, their potential off-target effects and cytotoxicity at high concentrations raise concerns regarding their long-term safety.^26^ Given these limitations, an alternative approach is required to optimize the therapeutic benefits of ginger-derived bioactive molecules while minimizing their adverse effects. Ginger has a long history of medicinal use, particularly in traditional medicine systems, for the treatment of inflammatory conditions. Accordingly, given that RA is a chronic inflammatory disease, the strong evidence supporting ginger’s ability to inhibit key RA-related processes—including synovial hyperplasia, cytokine overproduction, and cartilage degradation—makes it a rational candidate for EV-based exploration. Moreover, prior studies have demonstrated that the anti-inflammatory effects of ginger are mediated through bioactive molecules that can be packaged into EVs, suggesting that ginger-derived extracellular vesicles (GDEVs) could serve as a natural delivery platform for these agents. In this context, GDEVs have emerged as a promising solution to address these challenges. GDEVs encapsulate and protect bioactive molecules, enhancing their stability and bioavailability.^27^ Furthermore, EVs facilitate targeted delivery to immune cells and inflamed tissues, potentially improving therapeutic outcomes and reducing systemic side effects.^28^

Despite these advantages, the role of GDEVs in RA remains largely unknown. Given the potential to deliver bioactive miRNAs and modulate inflammatory pathways, we hypothesized that GDEVs could serve as a novel therapeutic approach for RA. This study aimed to investigate the efficacy of GDEVs in the treatment of RA using both *in vitro* and *in vivo* models and provide new insights into the potential of PDEVs as next-generation anti-inflammatory therapies. Furthermore, as GDEVs can be orally administered and naturally absorbed through the gastrointestinal tract, they represent a noninvasive and potentially better-tolerated alternative, especially compared to some current treatments such as JAKs.

## Results

### Characterization of ginger EVs

**Figure 1:**
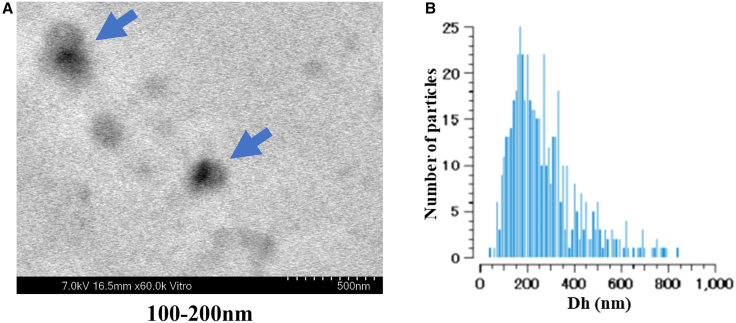
Characterization of GDEVs (A) SE-ADM images showing round vesicles with diameters of 100–200 nm (blue arrows indicate GDEVs). (B) Nanoparticle tracking analysis showing the particle size distribution and relative concentration of GDEVs. Dh, hydrodynamic diameter.

GDEVs were successfully isolated from ginger extract by differential centrifugation and ultracentrifugation, followed by filtration. Electron microscopy using a scanning electron-assisted dielectric microscopy (SE-ADM) system revealed that the isolated vesicles were predominantly in the exosome size range (100–200 nm), with some larger vesicles exceeding 200 nm (Figure 1A). Nanoparticle tracking analysis (VideoDrop, Meiwafosis, Japan) confirmed a median particle diameter of 239 nm and a concentration of 2.66 × 10^9^ particles/mL (Figure 1B, Table S2). The total protein concentration of GDEVs, measured using the Qubit Protein Assay (Qubit 2.0 Fluorometer, Invitrogen, USA), was 3.80 mg/mL (Table S2). Based on the yield calculation, approximately 47.5 mg of GDEVs were obtained per 1 kg of fresh ginger. These results are consistent with previous reports,^29^^,^^30^^,^^31^ supporting the successful isolation of GDEV.

### *In vitro* effects of GDEVs

To investigate the anti-inflammatory and anti-proliferative effects of GDEVs, we conducted a series of *in vitro* experiments using RASFs derived from human RA patients.

**Figure 2:**
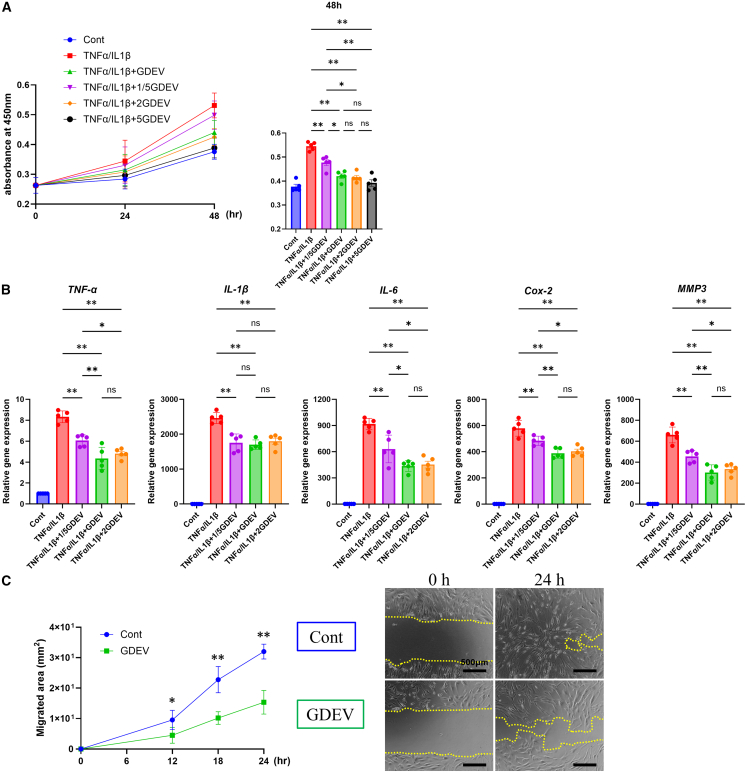
*In vitro* effects of GDEVs on RASF (A) Cell viability of RASF measured by MTT assay at 48 h. Six groups were analyzed: Cont, TNFα/IL1β, TNFα/IL1β + GDEV (0.2X), TNFα/IL1β + GDEV (1X), TNFα/IL1β + GDEV (2X), and TNFα/IL1β + GDEV (5X) (*n* = 5 per group). GDEVs dose dependently reduced RASF proliferation. (B) Gene expression analysis of inflammatory mediators in RASF treated with GDEVs (0.2X, 1X, 2X) under TNFα/IL1β stimulation (*n* = 5 per group). mRNA levels of TNFα, IL-1β, IL-6, COX-2, and MMP3 were measured by RT-qPCR. GDEVs significantly suppressed the expression of these inflammatory genes. (C) Scratch assay evaluating cell migration over 12, 18, and 24 h. Two groups were compared: Cont and TNFα/IL1β + GDEV (1X) (*n* = 5 per group). Representative images at 0 and 24 h are shown. GDEVs inhibited RASF migration. Cont, control. ∗∗*p* < 0.01, ∗*p* < 0.05.

Cell proliferation assays were performed using Cell Counting Kit-8 (CCK-8; Dojindo Laboratories, Kumamoto, Japan) at 24 and 48 h after inflammatory stimulation with TNFα and IL-1β. Compared to the inflammation-only group, treatment with GDEVs (1X) significantly reduced cell proliferation at 48 h by approximately 20% (Figure 2A). To evaluate inflammatory and catabolic gene expression, quantitative reverse-transcription polymerase chain reaction (RT-qPCR) was performed after 24-h treatment with GDEVs under inflammatory conditions. Compared to the inflammation-only group, GDEVs (1X) significantly suppressed mRNA expression of TNFα (35.1%) and IL-1β (23.2%), as well as downstream mediators IL-6 (50.4%) and cyclooxygenase-2 (COX-2) (30.7%). The catabolic enzyme MMP3 was also markedly reduced by 52.2% in GDEV-treated RASFs (Figure 2B). These genes are known to be involved in matrix degradation and inflammation in RA pathogenesis. After the analyses shown in Figure 2, we further evaluated the effects of GDEVs on cell proliferation and cytotoxicity to determine the optimal concentration for subsequent experiments. Cell proliferation was assessed using CCK-8, and cytotoxicity was examined by lactate dehydrogenase (LDH) release and Live/Dead staining (Figure S1). Higher concentrations of GDEVs (2X and 3X) showed a stronger inhibition of cell proliferation compared with the Control (Figure S1A). However, these concentrations also induced increased LDH release (Figure S1B), indicating cytotoxicity, and Live/Dead staining confirmed evident cell death at 2X and 3X (Figures S1C and S1D). In addition, concentrations above 2X induced cell detachment, further suggesting potential cytotoxicity. Based on these initial dose-response findings, 1X was selected as the maximum non-cytotoxic concentration for subsequent experiments. Based on the results shown in Figures 2A, 2B, and S1, the concentration corresponding to the green line (GDEV 1X) was selected as the standard dose for subsequent experiments. At this concentration, both cell proliferation and inflammatory gene expression were significantly suppressed compared with lower doses (0.2X), whereas increasing the dose did not provide additional benefits. Moreover, concentrations above 2X induced cell detachment, suggesting potential cytotoxicity. Therefore, GDEV 1X was considered the most effective and stable concentration for *in vitro* assays. Additionally, the scratch assay was used to assess cell migration. RASFs were either left untreated (Control) or treated with GDEVs (1X) under inflammatory conditions. Compared to the untreated RASFs, GDEV-treated cells showed significantly reduced migration over 24 h, with an approximate 52.1% decrease in the migrated area relative to the control group, as evidenced by slower closure of the scratched area (Figure 2C).

These data collectively demonstrate that GDEVs suppress inflammatory activation, proliferation, and migration of human RASFs *in vitro*.

### *In vivo* effects of GDEVs

**Figure 3:**
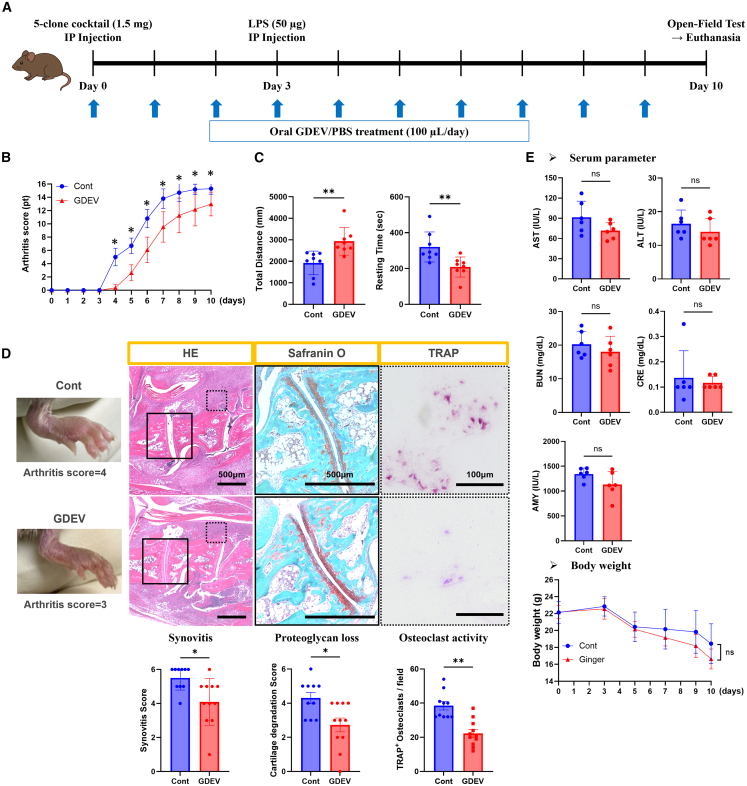
*In vivo* therapeutic effects of GDEVs in CAIA mice (A) Experimental protocol for CAIA induction and GDEV treatment. (B) Arthritis scores were assessed over time. Two groups were analyzed: Cont (*n* = 10) and GDEV-treated mice (GDEV, *n* = 11). GDEV treatment reduced arthritis severity. (C) Open-field test measuring total distance traveled and resting time at endpoint. Two groups: Cont (*n* = 8) and GDEV (*n* = 8). GDEVs improved locomotor activity and reduced resting time. (D) Representative macroscopic images of joint swelling and histological analysis. Synovitis (H&E staining), proteoglycan loss (safranin-O staining), and osteoclast activity (TRAP staining) were evaluated. Synovitis, cartilage degradation, and TRAP^+^ osteoclast scores are shown (Cont, *n* = 10; GDEV, *n* = 11). GDEV treatment significantly reduced synovitis, proteoglycan loss, and osteoclast activity. Solid and dotted squares in the images indicate the regions magnified for safranin-O and TRAP staining, respectively. (E) Serum biochemistry (AST, ALT, BUN, CRE, AMY) (Cont: *n* = 6, GDEV: *n* = 6) and body weight (Cont: *n* = 10, GDEV: *n* = 11) showed no systemic toxicity after GDEV treatment. pt, point; field, 500 μm × 500 μm; ns, not significant. ∗∗*p* < 0.01, ∗*p* < 0.05.

Oral GDEVs were evaluated for their therapeutic efficacy in a collagen antibody-induced arthritis (CAIA) mouse model (Figure 3A). Mice treated with oral GDEVs exhibited consistently lower arthritis scores compared to the control group from days 4 to 10, as assessed by a standardized clinical scoring system based on joint swelling and redness (Figure 3B, *p* < 0.05). Locomotor behavior was analyzed using an open-field test on day 10. GDEV-treated mice exhibited a greater total distance traveled (Control: 1,926.5 ± 550.8 mm, GDEV: 2,935.9 ± 644.5 mm, *p* < 0.01) and reduced resting time (Control: 320.6 ± 83.8 s, GDEV: 208.9 ± 56.0 s, *p* < 0.01), indicating improved mobility and reduced pain-related behavior (Figure 3C).

Histological analyses were performed on ankle joints collected at euthanasia. Hematoxylin and eosin (H&E) staining revealed that GDEV treatment significantly reduced synovial inflammation, with lower synovitis scores compared to the control group (Control: 5.5 ± 0.7, GDEV: 4.1 ± 1.4, *p* < 0.05). Safranin O-fast green staining demonstrated preservation of proteoglycan content in cartilage, as reflected by higher safranin O scores in the GDEV group (Control: 4.3 ± 1.1, GDEV: 2.7 ± 1.3, *p* < 0.05). Additionally, tartrate-resistant acid phosphatase (TRAP) staining revealed fewer TRAP-positive osteoclasts in GDEV-treated mice (Control: 38.5 ± 7.9, GDEV: 22.3 ± 7.6, *p* < 0.01), indicating suppressed bone resorption activity (Figure 3D). To further investigate fibroblast activation within the synovium, we performed fibroblast activation protein (FAP) immunofluorescence staining. The total number of 4′,6-diamidino-2-phenylindole (DAPI)^+^ nuclei did not differ significantly between groups (Control: 290.9 ± 118.3, GDEV: 284.3 ± 94.6). In contrast, the FAP-positive area (Control: 8,659.6 ± 7,482.1, GDEV: 2,611.0 ± 1,877.6, *p* < 0.05) was significantly reduced in the GDEV group (Figure S2).

To evaluate systemic safety, serum biomarkers—aspartate aminotransferase (AST), alanine aminotransferase (ALT), blood urea nitrogen (BUN), creatinine (CRE), and amylase (AMY)—which are widely recognized indicators of liver, kidney, and pancreatic function in preclinical toxicity studies,^32^ were analyzed, and body weights were monitored throughout the experiment. Compared to the control group, the GDEV-treated mice showed no significant alterations in serum parameters: AST (−21.7%), ALT (−14.7%), BUN (−11.0%), CRE (−14.6%), and AMY (−15.7%) (Figure 3E). Body weight changes were also comparable between the two groups on days 0 and 10 (Control: 22.1 ± 1.3 g to 18.4 ± 2.4 g; GDEV: 22.2 ± 0.8 g to 16.6 ± 1.2 g), with no statistically significant differences (Figure 3E). These findings indicate that oral GDEV administration did not induce systemic toxicity.

### Oral bioavailability of GDEVs

**Figure 4:**
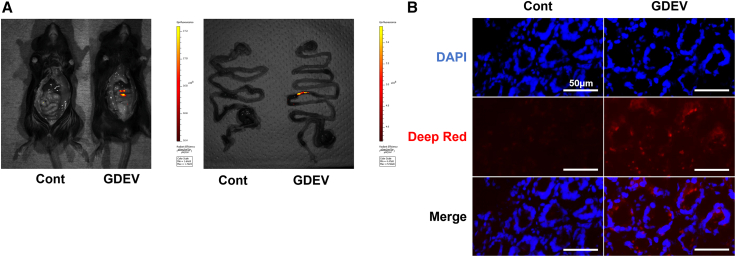
Gastrointestinal absorption of GDEVs (A) Fluorescence imaging using IVIS demonstrating uptake of orally administered GDEVs labeled with Aco-600 in mice. Mice administered free Aco-600 dye in PBS served as dye-only controls. (B) Representative frozen sections of the small intestine showing the distribution of Aco-600-labeled GDEVs. Nuclei were counterstained with DAPI (blue). Images are shown as DAPI (blue), Aco-600-labeled GDEVs (red), and merged images. Aco-600, fluorescent dye used to label GDEVs.

Fluorescence imaging using the In Vivo Imaging System (IVIS) Spectrum computed tomography (CT) system showed that fluorescently labeled GDEVs passed through the stomach without visible degradation and reached the small intestine within 2 h after oral administration. In contrast, mice administered the free Aco-600 dye (dye-only controls) showed no detectable intestinal fluorescence, confirming that the observed signal originated from the labeled GDEVs rather than the residual dye (Figure 4A). Subsequent fluorescence microscopy of frozen intestinal sections (prepared with Kawamoto’s film method and stained with DAPI) revealed uptake of EVs by intestinal villi (Figure 4B). Furthermore, immunofluorescence staining using an anti-junctional adhesion molecule-A (JAM-A) antibody demonstrated the localization of GDEVs within intestinal epithelial cells, confirming their internalization (Figure S3).

### Stability of GDEVs under simulated gastric conditions

**Figure 5:**
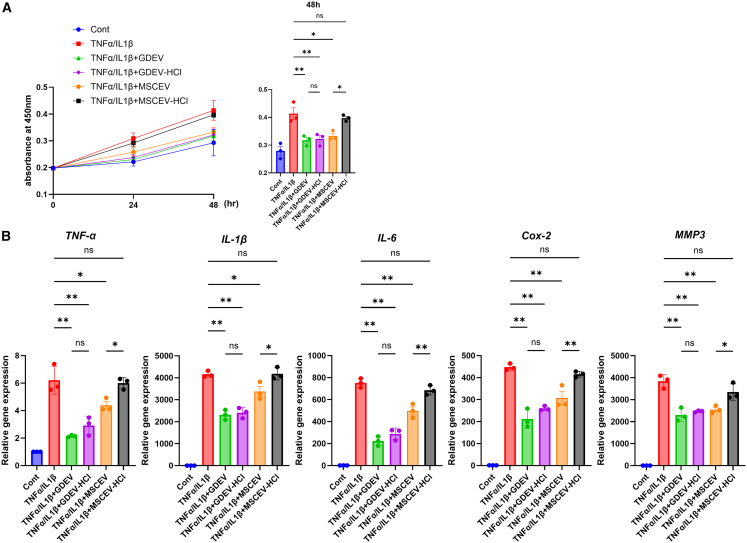
*In vitro* stability of GDEVs under simulated gastric conditions (A) Cell viability of RASF measured by MTT assay after 48 h. Six groups were analyzed (*n* = 3 per group): Cont, TNFα/IL1β, TNFα/IL1β + GDEV, TNFα/IL1β + GDEV-HCl, TNFα/IL1β + MSCEV, and TNFα/IL1β + MSCEV-HCl. GDEV-HCl retained proliferative activity equivalent to untreated GDEVs, whereas MSCEV-HCl showed reduced proliferation. (B) Gene expression analysis of inflammatory mediators (TNFα, IL-1β, IL-6, COX-2, and MMP3) by RT-qPCR under the same experimental conditions (*n* = 3 per group). GDEV-HCl maintained suppression of inflammatory gene expression comparable to untreated GDEVs, while MSCEV-HCl exhibited attenuated inhibitory effects. ns, not significant. ∗∗*p* < 0.01, ∗*p* < 0.05.

To determine whether GDEVs are stable under stomach-like acidic conditions, we performed *in vitro* assays comparing untreated and hydrochloric acid (HCl)-treated EVs. Specifically, GDEVs were exposed to HCl (pH 2.0, 60 min at 37°C), and their biological activity was compared with that of untreated GDEVs, as well as with mesenchymal stem cell-derived EVs (MSCEVs) and HCl-treated MSCEVs. Functional assays demonstrated that the proliferative effect of MSCEV-HCl was reduced by 19.5% compared with untreated MSCEVs, whereas GDEV-HCl retained proliferative activity equivalent to untreated GDEVs (Figure 5A). Similarly, the suppression of inflammatory gene expression (TNFα, IL-1β, IL-6, Cox2, and MMP3) was attenuated in MSCEV-HCl by 36.9%, 23.8%, 38.0%, 35.4%, and 31.4%, respectively, compared with untreated MSCEVs. In contrast, GDEV-HCl showed comparable suppressive effects to untreated GDEVs, indicating no loss of function after acid exposure (Figure 5B). These results suggest that GDEVs are resistant to gastric acid and maintain their biological activity, supporting their feasibility as orally administered therapeutic agents.

### Concentration of 6-gingerol and 6-shogaol in GDEVs

**Figure 6:**
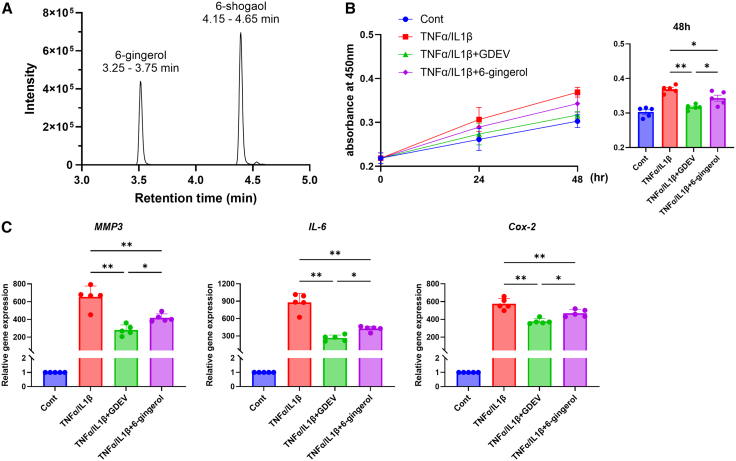
Evaluation of 6-gingerol and GDEVs on RASF (A) LC-MS chromatogram showing peaks of 6-gingerol (3.25–3.75 min) and 6-shogaol (4.15–4.65 min). (B) Cell viability of RASF measured by MTT assay (*n* = 5 per group). Four groups were analyzed: Cont, TNFα/IL1β, TNFα/IL1β + GDEV, and TNFα/IL1β + 6-gingerol. GDEVs significantly enhanced RASF viability compared with 6-gingerol. (C) Gene expression analysis of inflammatory mediators (MMP3, IL-6, and COX-2) in RASF following treatment (*n* = 5 per group). Four groups: Cont, TNFα/IL1β, TNFα/IL1β + GDEV, and TNFα/IL1β + 6-gingerol. GDEVs more effectively suppressed inflammatory gene expression compared with 6-gingerol. ∗∗*p* < 0.01, ∗*p* < 0.05.

Liquid chromatography-mass spectrometry (LC-MS) analysis using a triple quadrupole mass spectrometer (TQD, Waters Corporation, USA) (negative electrospray ionization mode) detected distinct peaks corresponding to the ginger bioactive compounds. 6-Gingerol was detected at a retention time of 3.25–3.75 min, while 6-shogaol was detected at 4.15–4.65 min (Figure 6A). Quantification indicated that 1 mg/mL of GDEV protein contained 6-gingerol at 5,312.3 ± 87.5 ng/mL (range: 5,225–5,400) and 6-shogaol at 137.3 ± 12.5 ng/mL (range: 125–150) (*n* = 3). These findings demonstrate that 6-gingerol is the predominant bioactive compound in GDEVs.

### Comparison of anti-inflammatory effects of GDEVs and 6-gingerol

Cell proliferation was assessed using the Cell Counting Kit-8 (CCK-8; Dojindo Laboratories, Kumamoto, Japan) in TNF-α/IL-1β-stimulated human RASFs. Treatment with GDEVs (1X) significantly reduced cell proliferation compared to the inflammation-only group (17.0% reduction) and the 6-gingerol group (10.3% reduction) (Figure 6B).

Similarly, RT-qPCR was performed to evaluate inflammatory and catabolic gene expression in the same cells. GDEV treatment markedly suppressed MMP3 (57.3%), IL-6 (70.0%), and COX-2 (34.4%) expression relative to the inflammation-only group. Notably, compared with the 6-gingerol group, GDEVs further decreased MMP3, IL-6, and COX-2 expression by 33.2%, 37.8%, and 19.6%, respectively (Figure 6C), indicating that GDEVs reduce inflammatory gene expression more effectively than 6-gingerol alone.

### RNA sequencing and miRNA analysis

**Figure 7:**
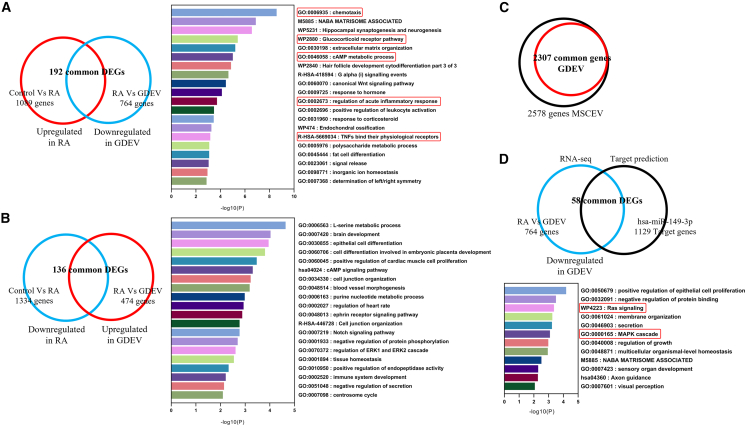
Transcriptomic analysis of GDEV effects on RASFs (A) DEGs upregulated in RASF under inflammatory conditions (RA group) and downregulated following GDEV treatment. GO enrichment clusters are shown. Genes related to arthritis exacerbation are boxed in red. A total of 192 genes were common between the comparisons (RA vs. control; GDEV vs. RA). (B) DEGs downregulated in RASF under inflammatory conditions (RA group) and upregulated following GDEV treatment. GO enrichment clusters are shown. A total of 136 genes were common between the comparisons. No arthritis-exacerbating pathways were enriched, indicating that GDEV treatment did not promote harmful gene expression. (C) Microarray comparison of human MSCEVs and GDEVs. A total of 2,578 miRNAs were detected in MSCEVs, of which 2,307 were also present in GDEVs. The top-expressed miRNAs (expression value > 1,000) were further analyzed, and miR-149 was selected as a candidate associated with anti-inflammatory effects. (D) RNA-seq analysis and miR-149 target gene identification. Downregulated DEGs in the GDEV-treated group (*n* = 764) were cross-compared with predicted miR-149 targets (*n* = 1,129) to identify 58 overlapping genes. GO enrichment analysis revealed modulation of key arthritis-related pathways, including Ras signaling and the MAPK cascade. Genes related to inflammation are boxed in red. Ras, Rat sarcoma virus oncogene.

RNA sequencing (RNA-seq) analysis revealed substantial transcriptional changes in RASFs after inflammation and following GDEV treatment. In Figure 7A, 1,089 genes were upregulated in the inflammation group compared with the control group, and 764 genes were downregulated in the GDEV group compared with the inflammation group. Among these, 192 genes were common to both comparisons. Gene Ontology (GO) enrichment analysis of these 192 genes identified that pathways such as chemotaxis, glucocorticoid receptor pathway, cAMP metabolic process, regulation of acute inflammatory response, and TNFs bind their physiological receptors, all of which are associated with arthritis exacerbation. Notably, these inflammation-related gene sets were suppressed by GDEV treatment.

In Figure 7B, 1,334 genes were downregulated in the inflammation group compared with the control group and 474 genes were upregulated in the GDEV group compared with the inflammation group. A total of 136 genes were common to both comparisons. GO analysis of these 136 genes did not reveal enrichment of arthritis-related pathways, indicating that GDEV treatment did not promote the expression of genes known to exacerbate arthritis.

To investigate whether miRNAs are associated with the anti-inflammatory effects of GDEVs, we performed microarray analysis to identify common miRNAs between MSCEVs, which have been reported to exert anti-inflammatory effects in RA^33^ and GDEVs. In Figure 7C, a total of 2,578 miRNAs were detected in MSCEVs, among which 2,307 were also present in GDEVs (the full list is available in the supplemental information file). Given the large number of shared miRNAs, we focused on the most abundant species by selecting those ranked within the top 20 in expression level and having expression values greater than 1,000 (Table S3). From this high-expression group, miR-149 was chosen because it has been previously reported in RASFs to inhibit cell proliferation and inflammatory cytokine expression,^34^ and it ranked among the top expressed miRNAs in GDEVs, making it a more generalizable candidate compared with the less-characterized miR-6087 and miR-6088.

Cross-comparison of predicted miR-149 target genes (*n* = 1,129, predicted using TargetScan) with RNA-seq of differentially expressed genes (DEGs) that were downregulated in the GDEV group compared to the inflammation group (*n* = 764) identified 58 overlapping genes (Figure 7D). GO enrichment analysis of these 58 genes revealed that key arthritis-related pathways were modulated, particularly Rat sarcoma virus oncogene (Ras) signaling and the mitogen-activated protein kinase (MAPK) cascade. Notably, genes such as Fms-related receptor tyrosine kinase 4 (FLT4), phospholipase A2 group IIA (PLA2G2A), kinase suppressor of Ras 1 (KSR1), and tau tubulin kinase 1 (TTBK1) were associated with Ras signaling, while insulin-like growth factor binding protein 4 (IGFBP4), SRY-box transcription factor 9 (SOX9), and dual specificity phosphatase 19 (DUSP19) were linked to the MAPK cascade (Table S4).

To experimentally validate these transcriptomic predictions, we next examined whether miR-149 could recapitulate the anti-inflammatory effects of GDEVs *in vitro*. Under TNF-α/IL-1β stimulation, cell proliferation assessed by CCK-8 was reduced by 13.4% with miR-149 compared to the inflammation-only group (Figure S4A). RT-qPCR further demonstrated that miR-149 significantly suppressed the expression of TNFα (46.0%), IL-1β (28.3%), IL-6 (54.8%), COX-2 (53.0%), and MMP3 (45.8%) relative to the inflammation-only group (Figure S4B).

**Figure 8:**
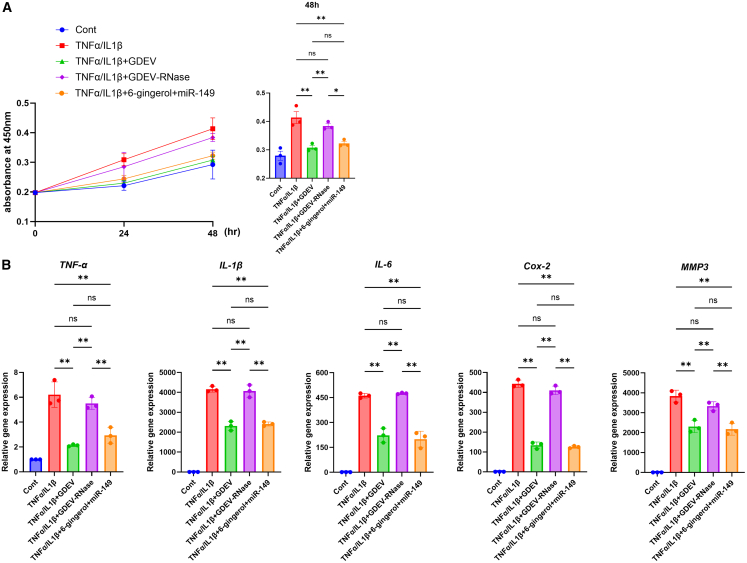
*In vitro* effects of RNA depletion of GDEVs and combination treatment with miR-149 and 6-gingerol on RASF (A) Cell viability of RASF measured by MTT assay after 48 h (*n* = 3 per group). Five groups were analyzed: Cont, TNFα/IL1β, TNFα/IL1β + GDEV, TNFα/IL1β + GDEV-RNase, and TNFα/IL1β + 6-gingerol + miR-149. The GDEV-RNase group showed significantly weaker suppression of proliferation compared with the GDEV group, whereas the combination of miR-149 and 6-gingerol reproduced anti-inflammatory effects comparable to intact GDEVs. (B) Gene expression analysis of inflammatory mediators (TNFα, IL-1β, IL-6, COX-2, and MMP3) by RT-qPCR under the same experimental conditions (*n* = 3 per group). The GDEV-RNase group exhibited attenuated suppression of inflammatory genes, while the combination of miR-149 and 6-gingerol reproduced effects similar to intact GDEVs. ∗∗*p* < 0.01, ∗ *p* < 0.05.

To further clarify whether miRNAs within GDEVs contribute to their anti-inflammatory effects, we compared the GDEV group, the GDEV treated with ribonuclease (GDEV-RNase) group, and a combination of miR-149 with 6-gingerol. The miR-149 + 6-gingerol group reproduced anti-inflammatory responses comparable to those of the GDEV group, showing 22% suppression of proliferation (Figure 8A) and reductions of TNFα (52.8%), IL-1β (42.0%), IL-6 (56.8%), COX-2 (71.8%), and MMP3 (43.4%) relative to the inflammation group (Figure 8B). In contrast, the GDEV-RNase group showed significantly weaker suppression of proliferation and inflammatory gene expression (proliferation: 13.2% vs. 22.0%; TNFα: 11.3% vs. 52.8%; IL-1β: 2.4% vs. 42.0%; IL-6: −3.0% vs. 56.8%; COX-2: 7.5% vs. 71.8%; MMP3: 13.2% vs. 43.4%) (Figure 8B). To examine whether miR-149 and 6-gingerol exert additive or synergistic effects, we compared the combined treatment with each single treatment. The miR-149 + 6-gingerol group showed an additional 14.9% inhibition of cell proliferation compared with miR-149 alone and 15.4% compared with 6-gingerol alone (Figure S5A). Similarly, the combined treatment produced greater anti-inflammatory effects, suppressing TNFα by an additional 31.0% (vs. miR-149) and 34.9% (vs. 6-gingerol), IL-1β by 22.3% and 22.5%, IL-6 by 28.1% and 32.2%, COX-2 by 61.8% and 58.3%, and MMP3 by 29.6% and 22.5%, respectively (Figure S5B). These findings suggest that the anti-inflammatory effects of GDEVs are at least partly attributable to their miRNA cargo, particularly miR-149, which may act synergistically with 6-gingerol.

## Discussion

This study revealed that oral administration of GDEV ameliorated arthritis in mice through its anti-inflammatory effects. GDEV contain high levels of miR-149 and 6-gingerol, both of which inhibited inflammation, cell proliferation, and migration in RASF, suggesting their therapeutic potential in RA. Our results highlight the potential for cross-kingdom communication, in which PDEVs, such as those from ginger, can influence mammalian cells.^35^ Unlike MSCEVs, GDEVs offer advantages, such as lower production costs, greater stability, and the added benefit of oral administration, making them viable for large-scale use.^36^

Previous studies have demonstrated that specific bioactive components of ginger, such as 6-gingerol and 6-shogaol, exert anti-inflammatory effects by modulating key pathways including NF-κB and MAPK (Table S5).^17^^,^^18^ In our study, GDEVs were found to contain higher levels of 6-gingerol compared to 6-shogaol, and importantly, GDEVs exhibited stronger anti-inflammatory effects than either compound alone. These findings indicate that the therapeutic efficacy of GDEVs cannot be attributed solely to the presence of ginger-derived small molecules. Our data further suggest that GDEVs provide enhanced therapeutic effects due to the synergistic action of bioactive molecules including miRNAs, proteins, and lipids, combined with efficient cellular uptake of EVs. Among these, miR-149 emerged as a key regulatory component. In line with this, our additional experiments demonstrated that RNase-treated GDEVs lost a significant portion of their anti-inflammatory capacity, supporting the conclusion that GDEV-associated RNase-sensitive cargos, particularly miRNAs, are functionally critical.

Compared to conventional anti-inflammatory therapies such as DMARDs, NSAIDs, and biologics, GDEVs offer several advantages. Conventional RA treatments are associated with high costs and adverse effects, including immunosuppression and increased risk of infection.^8^ In contrast, PDEVs provide a natural and potentially safer alternative with fewer side effects, particularly those derived from edible plants, which have been reported to exhibit minimal immunogenicity and favorable biocompatibility.^15^ Reported adverse effects of PDEVs are minimal, and their negative zeta potential contributes to colloidal stability, prevents aggregation, and enhances bioavailability.^36^ An additional critical advantage is their gastrointestinal stability. While previous studies mainly relied on fluorescent dyes (such as Aco-600) or morphological observation with transmission electron microscopy to suggest intestinal delivery, these approaches could not exclude dye release or failed to assess functional stability.^37^ In our study, mice administered free Aco-600 dye in PBS (dye-only controls) showed no detectable intestinal fluorescence, whereas Aco-600-labeled GDEVs were localized within JAM-A^+^ intestinal epithelial cells, confirming their cellular uptake and supporting their intestinal absorption. To extend these findings, we examined the stability of GDEVs under acidic conditions (HCl, pH 2.0) that mimic gastric acid. Notably, GDEVs maintained significant anti-inflammatory activity in CCK-8 and RT-qPCR assays after acid treatment, whereas MSCEVs lost activity under the same conditions. These results indicate that GDEVs are intrinsically resistant to acidic conditions, a property likely attributable to their lipid bilayer composition.^38^ This stability supports their feasibility as orally administered therapeutics. Thus, GDEVs, unlike many mammalian EVs, can remain intact and biologically active through the gastrointestinal tract.

In the present study, RNase treatment of GDEVs markedly reduced their anti-inflammatory effects, indicating that RNA cargos, including miRNAs, play a critical functional role. Analysis of these RNA cargos highlighted miR-149 as a key regulatory molecule mediating the anti-inflammatory effects of GDEVs. RNA-seq revealed that several genes in the Ras and MAPK pathways, including FLT4, PLA2G2A, KSR1, and DUSP19, were significantly downregulated in the EV-treated group compared to the inflammation group (Table S4). These genes are conserved targets of miR-149,^39^^,^^40^ suggesting that GDEVs exert their effects at least in part through miR-149-mediated suppression of pro-inflammatory signaling. Mechanistically, reduced FLT4 expression could modulate immune cell trafficking,^41^ while suppression of PLA2G2A may attenuate eicosanoid production.^42^ Consistent downregulation of KSR1 and DUSP19 further implies that GDEVs fine-tune MAPK activity to prevent excessive inflammation.^43^^,^^44^ These mechanistic insights align with our functional assays, where miR-149 suppressed proliferation and reduced expression of MMP-3, IL-6, COX-2, TNFα, and IL-1β. Taken together, our findings indicate that miR-149 appears to be a central mediator of the RNA-dependent anti-inflammatory activity of GDEVs, although other miRNAs and bioactive cargos may also contribute to the broader therapeutic effects.^16^

Despite these promising findings, this study had several limitations. First, although the *in vitro* experiments using RASFs clearly demonstrated the anti-inflammatory effects of GDEVs, to evaluate potential clinical applications, it is necessary to first confirm these effects *in vivo*. Considering the concept of cross-kingdom communication,^35^ in which PDEVs such as GDEVs can exert effects across species, a mouse model was used. As with previous studies on PDEVs, further preclinical research and clinical trials will be necessary to confirm the reproducibility of these findings in human patients with RA and to evaluate the safety and efficacy of GDEVs in clinical settings.^29^ Second, the bioavailability and pharmacokinetics of orally administered GDEVs require further investigation. Although our data confirmed intestinal uptake, the circulatory half-life and joint-specific accumulation of GDEVs remain unclear, as we did not measure blood concentration or percent injected dose. Furthermore, unlike mammalian EVs, GDEVs lack specific surface markers such as CD9 or CD63, limiting precise tracking of their biodistribution.^45^ In addition, we were unable to quantify whether miR-149 levels increased in the joint after oral GDEV administration. In the mouse arthritis model used in this study, the volume of synovial fluid and the size of joint tissues were extremely small, making reliable quantitative assessment difficult. Moreover, miR-149 is present in GDEVs at relatively low abundance and may be rapidly consumed within inflamed joint tissues, further complicating detection. Future studies using more sensitive analytical approaches or larger-animal models will be required to clarify the *in vivo* kinetics and joint accumulation of miR-149. However, GDEVs may exert systemic anti-inflammatory effects, potentially mediated in part through modulation of the gut microbiota, as reported in models of colitis.^31^ In our study, the observed functional outcomes provide indirect evidence that orally administered GDEVs can reach target tissues and exert anti-inflammatory effects. Future investigations should evaluate blood levels, joint distribution, and impacts on the gut microbiome in arthritis models. Third, the optimal dosing regimen and long-term effects of ginger EVs remain unclear. Although direct comparison with raw ginger was not performed in the present study, previous studies have shown that GDEVs contain higher levels of miRNAs and exert stronger anti-inflammatory effects than equivalent amounts of raw or cooked ginger.^16^ In the present study, the administered dose was equivalent to approximately 120 individual ginger units per 60-kg human body weight. One ginger unit weighed approximately 35 g, corresponding to a total of roughly 4.2 kg of fresh ginger. Direct consumption of such a large amount of ginger (≥4 g per day) could cause gastrointestinal discomfort or other adverse effects,^46^ whereas GDEVs can be concentrated and administered conveniently.^47^ Future investigations should evaluate the long-term effects, dose dependency, and optimal dosing intervals of GDEVs to establish safe and effective therapeutic regimens. Last, although miR-149 is a key anti-inflammatory component of GDEVs, the precise mechanisms remain incompletely understood. Combined administration of miR-149 and 6-gingerol reproduced effects comparable to GDEVs, suggesting these are major contributors. GDEVs also contain other miRNAs, proteins, and lipids, which may collectively enhance their therapeutic potential,^16^ highlighting their promise as a novel therapeutic strategy.

In conclusion, this study demonstrated that GDEVs exert significant therapeutic effects in RA, partly through RNA cargos such as miR-149 that mediate anti-inflammatory activity. Their oral availability, strong efficacy, and scalable production highlight the novelty and clinical potential of GDEVs as a natural and noninvasive therapeutic strategy for inflammatory diseases.

## Materials and methods

All procedures were performed in accordance with the Guidelines for Animal Experimentation, Hiroshima University and with the approval of the Committee of Research Facilities for Laboratory Animal Sciences, Graduate School of Biomedical Sciences, Hiroshima University (Approval number: A22-144).

### Isolation and characterization of GDEVs

#### Isolation of GDEVs

The GDEVs were isolated using the following procedure as previously described.^48^^,^^49^ Fresh ginger was chopped and homogenized using a blender, and the homogenate was squeezed using a food squeezer to obtain the crude extract. The extract was sequentially centrifuged at 300 × *g* for 10 min at 4°C, followed by 2,000 × *g* for 20 min at 4°C to remove cellular debris. The supernatant was then filtered through 0.45- and 0.22-μm filters. The filtered extract was ultracentrifuged at 39,500 rpm (approximately 100,000 × *g*) for 160 min at 4°C using a SW55Ti rotor (Beckman Coulter, Brea, CA, USA) in 5.2-mL open-top polypropylene tubes. After ultracentrifugation, the EV pellets were resuspended in 100 μL of phosphate-buffered saline (PBS) per tube, pooled together, and stored at 4°C. The isolated EVs were used for further analysis within 1 week.

#### High-resolution SE-ADM system

We evaluated EVs using an SE-ADM imaging system based on field emission scanning electron microscopy with an SU5000 (Hitachi High-Tech Corp., Japan), following a method described previously.^50^ This system enables observation of biological specimens in water without metal staining. Exosomes were imaged under the following conditions: magnification of 60,000×, resolution of 1,280 × 1,024 pixels, 40 s scanning time, 7 mm working distance, 7 kV accelerating electron beam, and 10 pA current. The original SE-ADM images were processed using a two-dimensional Gaussian filter (GF) with a kernel size of 7 × 7 pixels and a radius of 1.2 σ. Background subtraction was performed by subtracting the SE-ADM images from the filtered images by using a broad GF (400 × 400 pixels, 160 σ). EVs were identified based on their characteristic spherical morphology and a diameter of 50–150 nm, consistent with previous reports.^50^ Particles that were damaged, overlapping, or not clearly distinguishable from the background were excluded from analysis. Quantitative measurements were performed on at least three independent images per sample to ensure reproducibility.

#### Nanoparticle imaging analysis

The size distribution and concentration of the GDEVs were analyzed using a nanoparticle imaging analyzer (VideoDrop, Meiwafosis, Japan).^51^ This system enables real-time visualization and quantification of nanoparticles in a liquid suspension without the need for labeling. A diluted EV suspension (1:100 in PBS) was loaded into the VideoDrop chamber, and measurements were performed according to the manufacturer’s protocol. VideoDrop software (v.3.2) was used to determine particle size distribution and concentration.^51^ Each sample was analyzed in triplicate.

#### Protein quantification

The protein content of GDEVs was measured using the Qubit Protein Assay Kit (Invitrogen, Carlsbad, CA, USA) according to the manufacturer’s instructions.^52^ Briefly, the Qubit working solution was prepared using Qubit Reagent and Qubit Buffer. GDEV samples were mixed with the working solution, and fluorescence was measured using the Qubit 2.0 Fluorometer (Invitrogen, Carlsbad, CA, USA).

### *In vitro* experiment: Cell culture and assays

*In vitro* experiments were performed to evaluate the effects of GDEVs on inflammation and cellular function.

### Patients and RASF culture

Fresh synovial tissues were obtained from 5 RA patients (3 females, 2 males; mean age, 65.6 years; range, 57–75; mean disease duration, 12.2 years; range, 5–27) undergoing total knee arthroplasty, total hip arthroplasty, or total ankle arthroplasty at Hiroshima University (Table S6). All patients met the 2010 American College of Rheumatology/European Alliance of Associations for Rheumatology (ACR/EULAR) classification criteria for RA.^53^ The study protocol was approved by the Institutional Review Board of Hiroshima University (Approval No. E-508), and written informed consent was obtained from all participants.

Synovial tissue samples (approximately 0.5–1.0 cm^3^ each) were collected from three distinct biopsy sites per patient, selected on the basis of macroscopic signs of active inflammation, such as pronounced synovial hypertrophy, vascular proliferation, and erythema. RA affects multiple joints systemically. Thus, tissues were collected from various joints to reflect this systemic inflammation and minimize site-specific bias. Tissues were rinsed with PBS, minced into ∼1 mm^3^ fragments, and placed in 10-cm culture dishes containing Dulbecco’s modified Eagle medium ([DMEM], high glucose, with L-glutamine, phenol red, and sodium pyruvate; FUJIFILM Wako) supplemented with 10% fetal bovine serum ([FBS], Thermo Fisher Scientific) and 1% penicillin-streptomycin-amphotericin B (AB; FUJIFILM Wako).^54^ Cultures were maintained at 37°C with 5% CO_2_ in a humidified incubator, allowing fibroblast-like synoviocytes to migrate out from tissue explants. Nonadherent tissue pieces were removed after 5–7 days, and the medium was replaced every 3–4 days.

Although RASFs at passage 2 maintain morphological stability, they may still reflect acute inflammatory status from the donor, leading to sample variability. Fourth to sixth passages of RASFs were used for subsequent experiments to ensure high fibroblast purity, reduce patient variability, and maintain RA-specific inflammatory phenotypes.^55^

### Induction of inflammation

To induce an inflammatory response, cells were treated with a combination of TNF-α (5 ng/mL, PeproTech, Rocky Hill, NJ, USA) and IL-1β (5 ng/mL, PeproTech, Rocky Hill, NJ, USA) for 24 h. For cell proliferation assays, the cells were treated with TNF-α and IL-1β for 24 h or 48 h, and the proliferation was assessed at both time points. This inflammatory model was selected based on previous studies, which have shown that TNF-α and IL-1β are key pro-inflammatory cytokines involved in RA pathogenesis.^3^^,^^56^ This ensured a reproducible inflammatory model regardless of donor variability.

### Cell proliferation assay

Cell proliferation was evaluated using the 3-(4,5-dimethylthiazol-2-yl)-2,5-diphenyltetrazolium bromide (MTT) Assay (CCK-8; Dojindo Laboratories, Kumamoto, Japan).^49^ RASFs were seeded in 96-well plates at a density of 3 × 10^3^ cells/well and cultured for 15 h in DMEM (high glucose, without L-glutamine and phenol red; FUJIFILM WAKO) supplemented with 10% FBS and 1% AB. After this, the following treatment groups were formed: Control group (100 μL/well), DMEM 100 μL; inflammatory group (100 μL/well), DMEM 100 μL + TNFα (5 ng/mL) + IL-1β (5 ng/mL); EV group (100 μL per well), DMEM 95 μL + TNFα (5 ng/mL) + IL-1β (5 ng/mL) + GDEVs (0.5 μL at various concentrations) + PBS (4.5 μL). The GDEVs were diluted in PBS to achieve the final concentration, with the total volume of EVs and PBS adjusted to 5 μL (GDEVs 0.5 μL + PBS 4.5 μL). After treatment, the CCK-8 reagent was added to each well, and the cells were incubated for 1 h. Absorbance was measured at 450 nm using a microplate reader (Tecan Infinite 200 PRO, Tecan Group Ltd., Männedorf, Switzerland) at 0, 24, and 48 h to evaluate cell proliferation.

### Gene expression analysis by RT-qPCR

RT-qPCR was performed to evaluate the expression levels of catabolic factors associated with arthritis, as described in our previous reports.^57^ Total RNA was extracted from RASFs using ISOGEN reagent (Nippon Gene, Tokyo, Japan) and RNA purification kit (Direct-zol RNA Microprep, Zymo Research). Complementary DNA (cDNA) was synthesized using the iScript Supermix Reverse Transcription System (Bio-Rad Laboratories, Hercules, CA, USA) according to the manufacturer’s protocol. Real-time PCR was performed using TaqMan Gene Expression Assay probes (Thermo Fisher Scientific, Waltham, MA, USA) for the following inflammation-related cytokines and enzymes involved in cartilage degradation: TNF-α, IL-1β, IL-6, Cox-2, and MMP3. The assay ID for each gene is listed in the supplemental information (Table S7). The RASFs were seeded in 24-well plates at a density of 5 × 10^4^ cells/well and cultured for 15 h in DMEM supplemented with 10% FBS and 1% AB. After this, the following treatments were applied for 24 h: Control group: DMEM (500 μL); inflammatory group: DMEM (500 μL) + TNFα (5 ng/mL) + IL-1β (5 ng/mL); EV group: DMEM (480 μL) + TNFα (5 ng/mL) + IL-1β (5 ng/mL) + GDEVs (2 μL) + PBS (18 μL). After 24 h of treatment, total RNA was extracted using ISOGEN reagent, and 120 ng of RNA was used for reverse transcription to synthesize cDNA for subsequent RT-qPCR analysis. Gene expression was normalized to that of glyceraldehyde-3-phosphate dehydrogenase (GAPDH), which was used as the internal control. The delta-delta cycle threshold (ΔΔCt) method was used to calculate relative gene expression levels.

### Scratch assay for cell migration

The effect of GDEVs on cell migration was evaluated using the scratch assay.^57^ RASFs were seeded in 24-well plates at a density of 5 × 10^4^ cells/well and allowed to reach confluence. Once confluence was reached, a straight-line scratch was made through the cell monolayer using a 200 μL pipette tip. After scratching, the cells were washed twice with PBS and replaced with DMEM containing 10% FBS and 1% AB. The following treatment groups were used: Control group (DMEM) and GDEVs group (DMEM + ginger EVs). Migration was monitored using an inverted microscope (Olympus, Tokyo, Japan), and images of the scratched areas were captured at 0, 12, 18, and 24 h. The migrated area was quantified by measuring the difference in the scratch area at each time point relative to the initial area at 0 h using ImageJ software (v.1.53p; NIH, USA).

### *In vivo* experiment

#### Animal model and treatment

Male DBA/1J mice (Japan SLC, Tokyo, Japan) aged 7–9 weeks (body weight, mean 22.2 ± 1.1 g, range 19.6–24.0 g; *n* = 21) were used in this study, as this strain and sex are commonly employed in CAIA models according to previous reports.^58^ After 1 week of acclimatization, mice were housed in temperature-controlled quarters (23°C ± 1°C) with a 12-h light-dark cycle, in groups of two to five per cage (S cage: 143 mm × 293 mm × H148 mm). All mice had free access to food and water. CAIA was induced using a CAIA induction kit (Chondrex, Redmond, WA, USA) as previously reported.^59^ Mice were injected intraperitoneally (IP) with a 5-clone monoclonal antibody cocktail (1.5 mg) on day 0, followed by an IP injection of lipopolysaccharide (50 μg) on day 3 to enhance arthritis induction. The mice were monitored daily for development of arthritis. GDEVs or PBS were administered orally. The treatment groups were as follows: EV group (*n* = 11): GDEV 20 μL + PBS 80 μL = 100 μL/day, Control group (*n* = 10): PBS 100 μL/day. Mice were euthanized on day 10 with isoflurane anesthesia followed by cervical dislocation, based on previous reports using the DBA/1J CAIA model.^58^ On sacrifice, blood and bilateral ankle joints were collected for further analysis.^60^

#### Arthritis score evaluation

Arthritis severity was assessed using a qualitative clinical scoring system based on joint swelling and redness, as described previously.^61^^,^^62^ Each paw was scored individually using the following criteria: 0, normal; 1, mild but definite redness and swelling of the ankle or wrist or redness and swelling limited to individual digits; 2, moderate redness and swelling of the ankle or wrist; 3, severe redness and swelling of the entire paw, including the digits; and 4, maximally inflamed limb involving multiple joints. The total arthritis score was calculated as the sum of the scores from all four paws, with a maximum possible score of 16 points.

#### Open-field test

To assess locomotor activity and pain-related behavior, mice were subjected to an open-field test on day 10. The test was conducted in a square arena (100 cm long × 100 cm wide × 60 cm high) under a central light intensity of 500 lx based on a previously described method.^63^ Each mouse was placed in a corner of the arena and allowed to explore freely for 10 min. Locomotor activity was automatically recorded using a tracking system (SMART; Panlab SL, Barcelona, Spain). The total distance traveled and resting time were measured as key parameters to evaluate movement and pain-related behavior.

#### Histological analysis

At the time of euthanasia, the ankle joints were collected and fixed in 4% paraformaldehyde (PFA) at 4°C for 48 h. The samples were then decalcified using an ethylenediaminetetraacetic acid (EDTA)-based neutral decalcifying solution (EDT-X; FALMA Co., Tokyo, Japan) at room temperature for 4 days. After decalcification, the tissues were embedded in paraffin and sectioned into 6-μm-thick slices. Histological evaluation was performed using HE, safranin-O, and fast green staining, as described in our previous reports.^49^^,^^57^ To evaluate osteoclast activity, TRAP staining was performed using a commercially available kit (Wako Pure Chemical Industries, Ltd., Osaka, Japan) according to the manufacturer’s protocol.^64^ Several parameters were used in the histological scoring of the foot joints^65^^,^^66^: Synovitis score: For synovial inflammation, high-power magnification fields (HPFs) were scored for the percentage of infiltrating mononuclear inflammatory cells as follows: 0 = absent, 1 = mild (1%–10%), 2 = moderate (11%–50%), and 3 = severe (51%–100%). Cartilage degradation score: Cartilage degradation was assessed based on safranin-O staining of proteoglycans, with results expressed as the percentage of cartilage that lost staining: 0 = no loss of staining, 1 = mild loss (1%–10%), 2 = moderate loss (11%–50%), 3 = severe loss (51%–100%). TRAP+ osteoclast score: TRAP-positive multinucleated cells containing more than three nuclei were identified as osteoclasts. Osteoclast counts were determined by evaluating three fields (500 μm × 500 μm each) and calculating their average. Observations and measurements were performed using a BZ-X710 all-in-one microscope (KEYENCE Corporation, Osaka, Japan) and its software. Histological scores were calculated as the sum of both left and right joints. Three different researchers, blinded to the experimental groups, performed the scoring.

#### Serum parameter evaluation and body weight monitoring

Blood samples were collected via cardiac puncture after euthanasia. After coagulation at 4°C for 1 h, the blood samples were centrifuged at 1,500 × *g* for 30 min at 4°C. The supernatants were collected for subsequent analyses.^60^ Serum biomarkers, including AST, ALT, BUN, CRE, and AMY, were analyzed at Nagahama Life Science Laboratory (Nagahama, Japan) using routine laboratory methods. Specifically, AST, ALT, and AMY were measured using the standardized method of the Japan Society of Clinical Chemistry, whereas BUN and CRE were measured using enzymatic methods. These analyses were performed to assess the potential systemic side effects of the treatment. Additionally, changes in body weight were monitored on days 0, 3, 5, 7, 9, and 10 to evaluate any systemic or side effects of the treatment.

### Evaluation of oral absorption of GDEVs

#### Fluorescence imaging analysis

Fluorescence imaging was performed using an IVIS Spectrum CT instrument (PerkinElmer Inc., Waltham, MA, USA).^67^ The EVs were labeled with Aco-600 (Acoerela, Inc., Singapore) at a final concentration of 5 μM before administration.^68^ Living Image 4.3.1 software was used to analyze the images. The excitation filter was set at 640 nm to capture the fluorescence signals. C57BL/6J male mice (8–10 weeks old; Japan SLC, Tokyo, Japan) were orally administered either labeled GDEVs (20 μL GDEVs +80 μL PBS = 100 μL) or free Aco-600 dye in PBS (5 μM, 100 μL; dye-only control) after an 18-h fasting period, under the same dosing regimen described in the in vivo experiment section. Fluorescence imaging was performed 2 h post-administration using the IVIS system.

#### Tissue distribution analysis (fluorescence microscopy on frozen sections)

At the designated time points, the animals were euthanized, and the small intestine, where fluorescence was detected via IVIS, was harvested. The tissues were immediately frozen in Optimal Cutting Temperature (OCT) compound (Tissue-Tek, Sakura Finetek, Tokyo, Japan) and sectioned into 5-μm thick slices using a CryoStar NX50 (Thermo Fisher Scientific, Waltham, MA, USA) with Kawamoto’s film method (SECTION-LAB, Co. Ltd., Yokohama, Japan). Briefly, Kawamoto’s film method employs a special adhesive film to support the frozen tissue during sectioning, which allows preparation of high-quality thin sections without curling or fragmentation, even from fragile or undecalcified tissues.^69^ The frozen sections were stained with DAPI (Dojindo Laboratories Co., Ltd. Kumamoto, Japan) to visualize nuclei and were then analyzed using a BZ-X710 all-in-one fluorescence microscope (KEYENCE, Osaka, Japan).^57^

#### *In vitro* stability of GDEVs under simulated gastric conditions

To evaluate the stability of GDEVs in stomach-like acidic conditions, GDEVs were incubated with HCl (pH 2.0; Fujifilm Wako Pure Chemical, Tokyo, Japan) at 37°C for 1 h. After incubation, the samples were re-purified by ultracentrifugation at 39,500 rpm for 160 min at 4°C to remove residual acid.^37^^,^^70^ The same procedure was applied to MSCEVs to obtain HCl-treated MSCEVs. The recovered GDEV-HCl and MSCEV-HCl were subsequently applied to RASF for functional assays. Cell proliferation was assessed using the method described above, and the expression levels of inflammatory genes (TNFα, IL1β, IL6, Cox2, and MMP3) were determined by RT-qPCR. Untreated GDEVs and untreated MSCEVs were used as controls.

#### 6-Gingerol concentration measurement

The beneficial effects of ginger can be attributed to biologically active compounds in its rhizome, such as gingerols and shogaols, which are known for their anti-inflammatory properties.^18^ Among the various bioactive compounds found in ginger, 6-gingerol is the most abundant and pharmacologically active constituent.^71^^,^^72^ To quantify the concentration of 6-gingerol in GDEVs, LC-MS analysis was performed by TQD triple quadrupole mass spectrometer (Waters, USA) using negative-mode electrospray ionization (ESI−).^37^ A reference standard of 6-gingerol (purchased from Selleckchem, USA) was used for calibration, and curcumin was employed as internal standard for analysis.^73^ The parameters of the mass spectrometer were optimized as follows: capillary voltage, −3,000 V; core voltage, −20 V for 6-gingerol, −22 V for curcumin; source temperature, 120°C; desolvation temperature, 350°C; desolvation gas flow, 600 L/h; cone gas flow, 50 L/h; collision, 10 for gingerol and 18 for curcumin detection; the m/z 293 > 193 and m/z 367 > 149 ion transitions were employed for gingerol and curcumin detection as (M-H)−. Chromatographic separation was using ACQUITY UPLC system and BEH C18 column (130 Å, 1.7 μm, 2.1 mm × 50 mm, Waters, USA).^73^ Chromatographic separations were carried out at 40°C. The mobile phases consisted of 10% acetonitrile containing 0.1% formic acid (A) and 10% acetonitrile containing 0.1% formic acid (B). Gradient elution at a flow of 200 μL/min was performed with changing %A as follows: 0–7 min: 0%–100%; 7.5–8.5 min: 100%; 8.5–9 min 100%–0%: 9–10 min: 0%. The extracted EV samples were analyzed using an LC-MS system operated under optimized conditions, including multiple reaction monitoring (MRM) mode, to ensure high sensitivity and specificity.

### RNA-seq and miRNA analysis

#### RNA-seq analysis (DEG analysis and pathway analysis)

RNA-seq was done to analyze the gene expression profiles of Control, Inflammation, and GDEV-treated RASFs.^74^ Low-quality reads (such as those containing adaptor contamination or with low Phred quality scores) were filtered out to ensure that only high-quality reads were retained, and the remaining high-quality reads were mapped to the human reference genome (GRCh38). Gene expression was normalized using appropriate methods for RNA-seq, and DEGs were identified between the following groups: Control vs. Inflammation and Inflammation vs. GDEV-treated.^75^ The RNA-seq data were submitted for analysis to Bioinformatics, Inc. (Tokyo, Japan), where the gene expression data were evaluated. DEGs were then subjected to GO enrichment analysis using the gprofiler2 (v.0.2.1) and clusterProfiler (v.4.6.2) packages to identify biological processes, cellular components, and molecular functions associated with gene expression changes. These R packages are widely used for functional enrichment and pathway analysis, allowing the identification of overrepresented GO terms and pathways from DEG lists and providing robust statistical control for multiple testing.^76^^,^^77^ These analyses provide insights into the biological mechanisms underlying the therapeutic effects of GDEVs.

#### Microarray analysis of miRNA

Microarray analysis was performed using a human miRNA chip (Filgen, Inc., Nagoya, Japan) to detect hsa-miRs enriched in GDEVs.^78^ The hsa-miRNA profiles were compared with those of MSCEVs to identify common hsa-miRs, as MSCEVs are also known to contain therapeutic miRNAs.^79^^,^^80^ The analysis focused on hsa-miRs, which are potentially involved in the therapeutic effects of GDEVs. Among the detected miRNAs, those with expression values greater than 1,000 were selected for further investigation, as highly abundant miRNAs in EVs are more likely to exert functional effects due to their competitive binding to target mRNAs.^81^ Target genes of highly expressed miRNAs were identified using TargetScan (https://www.targetscan.org), and a cross-study comparison was performed with genes identified by RNA-seq, which had showed decreased expression following GDEV administration. This approach helped identify common miRNA-targeted genes and those downregulated by GDEV treatment. Commonly expressed genes were extracted and subjected to GO enrichment analysis using Metascape (https://metascape.org) to identify the associated biological processes, cellular components, and molecular functions associated with the identified genes.^82^

#### Transfection of miR-149-3p mimic into RASF

See supplemental information.

#### RNA depletion of GDEVs and combination treatment with miR-149 and 6-gingerol

To deplete RNA from GDEVs, purified GDEVs were mixed with RNase (10 μg/mL; Nippon Gene, Tokyo, Japan) and incubated at 37°C for 1 h. The GDEV-RNase was subsequently washed with PBS and pelleted by ultracentrifugation (39,500 rpm, 160 min, 4°C).^83^ The resulting GDEV-RNase was used for functional assays.

For combination experiments, RASF were transfected with miR-149 mimics as described in the supplemental information and treated with 6-gingerol at the same concentration as described above.

### Statistical analysis

Statistical analyses were performed using GraphPad Prism 10.0 (San Diego, CA, USA). Data were analyzed using an unpaired *t* test for comparisons between two groups and one-way ANOVA with Tukey’s post-hoc test for comparisons among three or more groups. Tukey’s post-hoc test was applied to correct for multiple comparisons and is widely used to identify specific group differences after ANOVA.^84^ Results are presented as mean ± standard deviation. For all statistical analyses, a *p* value <0.05 was considered statistically significant.

## Data availability

The datasets during and/or analyzed during the current study are available from the corresponding author on reasonable request.

## Acknowledgments

We thank E. Ueda, T. Miyata, and Y. Takagi for their technical support. We would like to thank Editage (www.editage.com) for English language editing. This research was supported by a 10.13039/100019522JCR Grant for Promoting Research for Early RA, the JSPS Program for Forming 10.13039/501100001691Japan’s Peak Research Universities (JSPS J-PEAKS), and the 10.13039/100009619Japan Agency for Medical Research and Development (AMED) under the HK^2^-MIRAI Program (grant number JP256f0137011). The LC-MS analysis was conducted using facilities at the 10.13039/501100024233Natural Science Center for Basic Research and Development (N-BARD) and 10.13039/501100003790Hiroshima University (NBARD-0100).

## Author contributions

H.K., T.N., and S.M. contributed to the study conception and design. Material preparation, experimental procedures, and data analysis were performed by H.K., T.N., Y.D., D.M., R.K., T.O., and S.M. The first draft of the manuscript was written by H.K., and all authors commented on previous versions of the manuscript. All authors read and approved of the final manuscript.

## Declaration of interests

The authors declare no competing interests.

## Supplemental information

Document S1. Figures S1–S5 and Tables S1–S7

Table S8. List of common miRNAs between MSCEVs and GDEVs

Document S2. Article plus supplemental information
