# Supplementary material for: Oral ginger-derived extracellular vesicles ameliorate arthritis via anti-inflammatory actions of microRNA-149 and 6-gingerol
Source: Mol Ther Nucleic Acids. 2026 Jan 19;37(1):102840. doi: 10.1016/j.omtn.2026.102840 (PMC12887272; doi:10.1016/j.omtn.2026.102840)
Supplement: Document S1. Figures S1–S5 and Tables S1–S7 [file mmc1.pdf]

OMTN, Volume *37*

Supplemental information

Oral ginger-derived extracellular vesicles ameliorate arthritis via anti-inflammatory actions of microRNA-149 and 6-gingerol

Hiroki Kaneta, Tomoyuki Nakasa, Dilimulati Yimiti, Dan Moriwaki, Riku Kawasaki, Toshihiko Ogura, Shigeru Miyaki, and Nobuo Adachi

Supplemental Figures

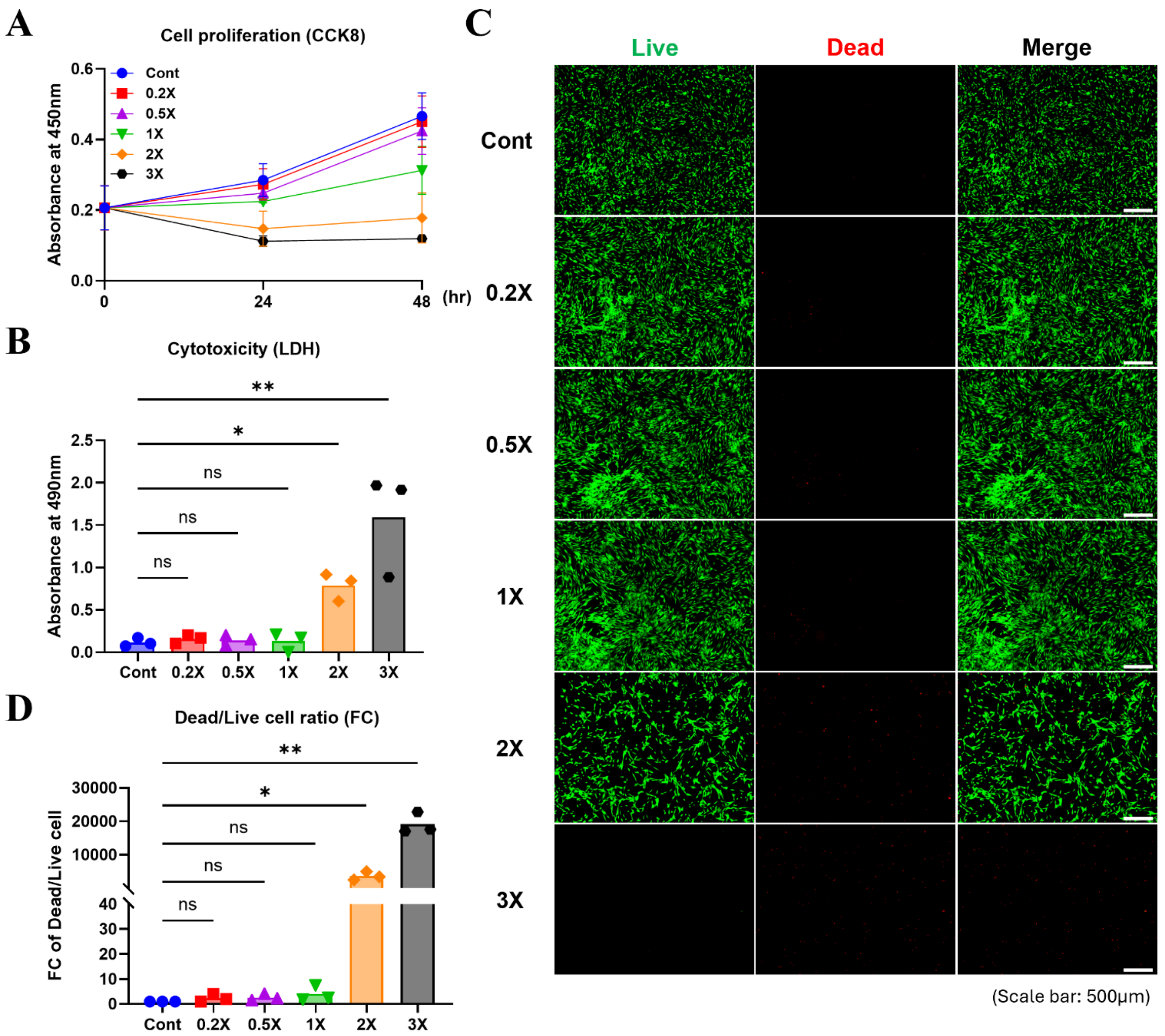

Figure S1. Dose-dependent effects of GDEVs on RASF proliferation and cytotoxicity. (A) Cell viability of RASF treated with different concentrations of GDEVs (0.2X, 0.5X, 1X, 2X, 3X) for 24 and 48 h, measured using the CCK-8 assay (n = 3 per group). Higher concentrations (2X and 3X) showed stronger inhibition of proliferation compared with 1X. (B) Cytotoxicity assessed by LDH release after 24 h of treatment with various concentrations of GDEVs (n = 3 per group). LDH release increased markedly at concentrations ≥ 2X, indicating cytotoxicity. (C, D) Live/Dead staining of RASF treated with different GDEV concentrations. In

the fluorescence images, live cells appear green and dead cells appear red. Representative fluorescence images (C) and quantification of live/dead cell ratios (D) are shown (n = 3 per group). GDEVs at 2X and 3X induced evident cell death, whereas 1X did not show cytotoxic effects. GDEV, ginger-derived extracellular vesicles; RASF, rheumatoid arthritis synovial fibroblasts; CCK-8, Cell Counting Kit-8; LDH, lactate dehydrogenase; FC, Fold Change; cont, control; ns, not significant. **, $p < 0.01$; *, $p < 0.05$.

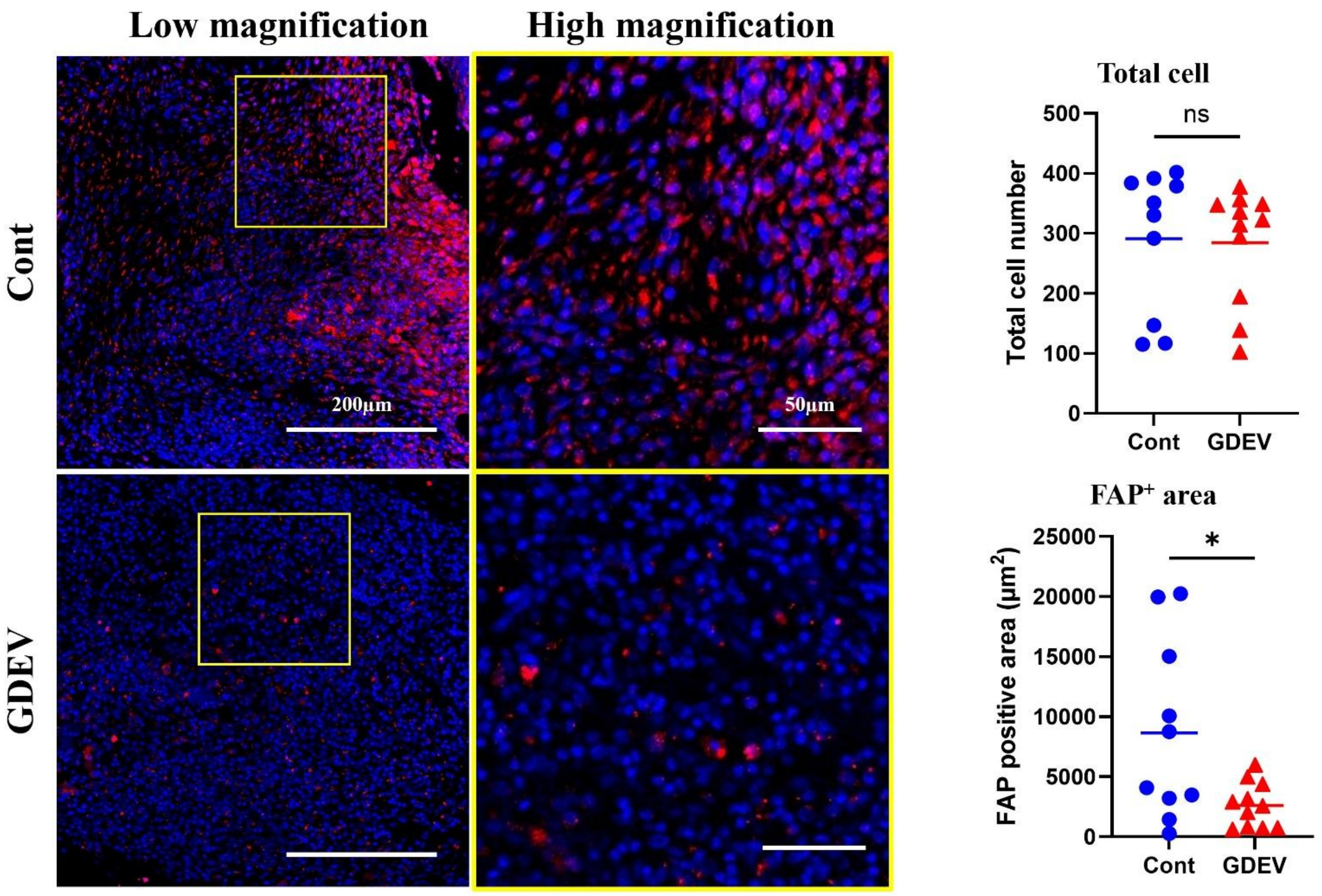

Figure S2. FAP immunofluorescence staining in CAIA mice. Representative images of synovial tissue stained with anti-FAPα antibody (red) and DAPI (blue) are shown at low (left) and high (right) magnification. Yellow squares in low-magnification images indicate regions of interest (200 μm × 200 μm). Quantification of total $DAPI^{+}$ nuclei and FAP-positive area are shown in the graphs. GDEV treatment did not significantly affect total $DAPI^{+}$ nuclei, but significantly reduced FAP-positive area compared with the control group (Cont: n = 10, GDEV: n = 11). FAP, fibroblast activation protein-α; CAIA, collagen antibody-induced arthritis; DAPI, 4′,6-diamidino-2-phenylindole; GDEV, ginger-derived extracellular vesicle; cont, control; ns, not significant. *, $p < 0.05$

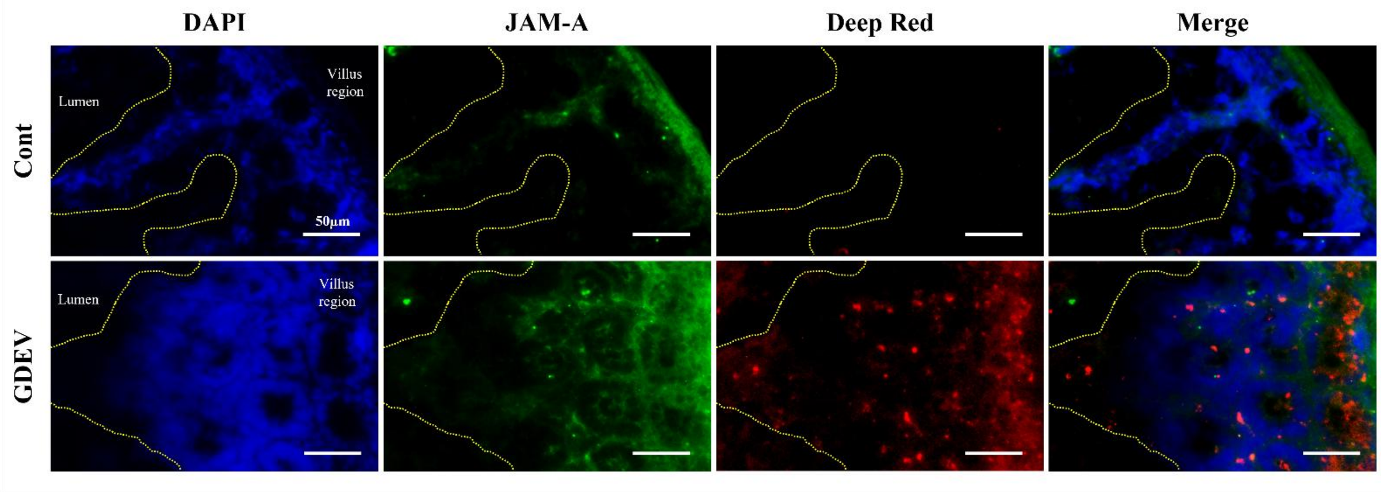

Figure S3. Uptake of GDEVs by intestinal epithelial cells (immunofluorescence for JAM-A).

Representative frozen sections of the small intestine obtained from the same experimental conditions shown in Figure 4, with additional immunofluorescence staining for JAM-A to identify intestinal epithelial cells. Nuclei were counterstained with DAPI (blue), GDEVs were labeled with Aco600 (red), and JAM-A was visualized in green. The luminal side is shown on the left, and the intestinal epithelial layer (villus region) is on the right. Merged images demonstrate the localization and internalization of Aco600-labeled GDEVs within JAM-A^{+} epithelial cells. GDEV, ginger-derived extracellular vesicles; DAPI, 4′,6-diamidino-2-phenylindole; Aco600, fluorescent dye used to label GDEVs; JAM-A, junctional adhesion molecule-A; cont, control.

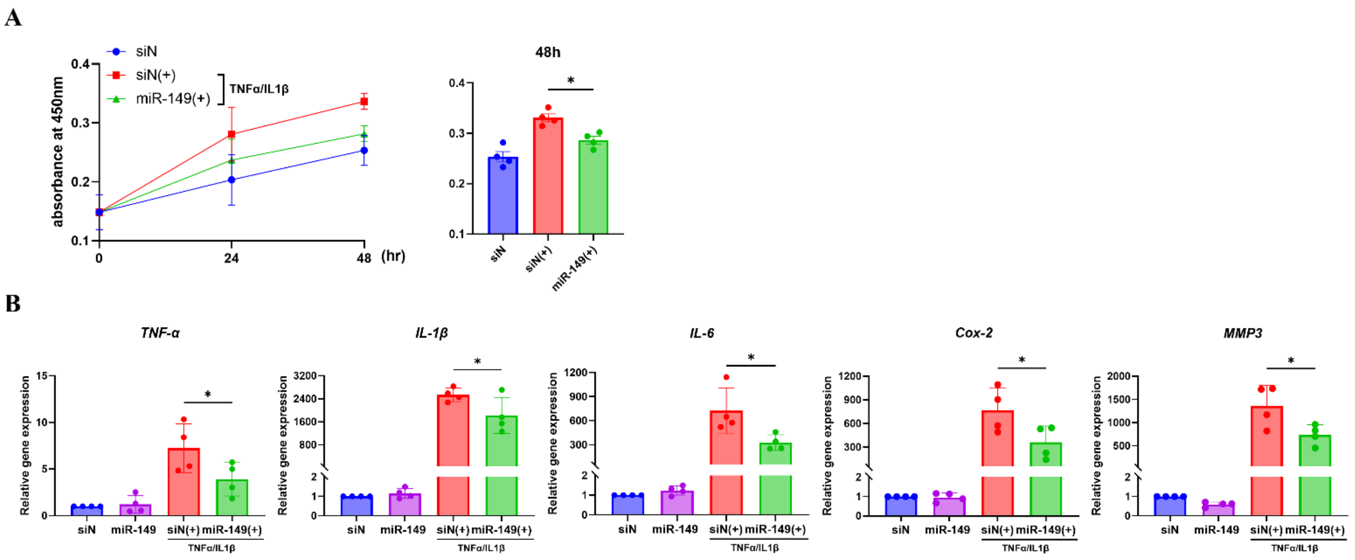

Figure S4. In vitro analysis of miR-149 effects on RASF. (A) Cell viability of RASF measured by MTT assay (n = 4 per group). Three groups were analyzed: SiN, SiN + TNFα/IL1β, TNFα/IL1β + miR-149. MiR-149 suppressed RASF proliferation under TNFα/IL1β stimulation. (B) Gene expression analysis of inflammatory mediators (TNFα, IL-1β, IL-6, COX-2, and MMP3) by qRT-PCR under the same experimental conditions (n = 4 per group). Four groups were analyzed: SiN, miR-149, SiN + TNFα/IL1β, and TNFα/IL1β + miR-149. MiR-149 significantly suppressed expression of these inflammatory genes compared with the inflammation-only group. RASF, rheumatoid arthritis synovial fibroblasts; MTT, 3-(4,5-dimethylthiazol-2-yl)-2,5-diphenyltetrazolium bromide; SiN, scrambled negative control; TNFα, tumor necrosis factor alpha; IL-1β, interleukin-1 beta; IL-6, interleukin-6; COX-2, cyclooxygenase-2; MMP3, matrix metalloproteinase-3; qRT-PCR, quantitative reverse-transcription polymerase chain reaction. *, $p < 0.05$

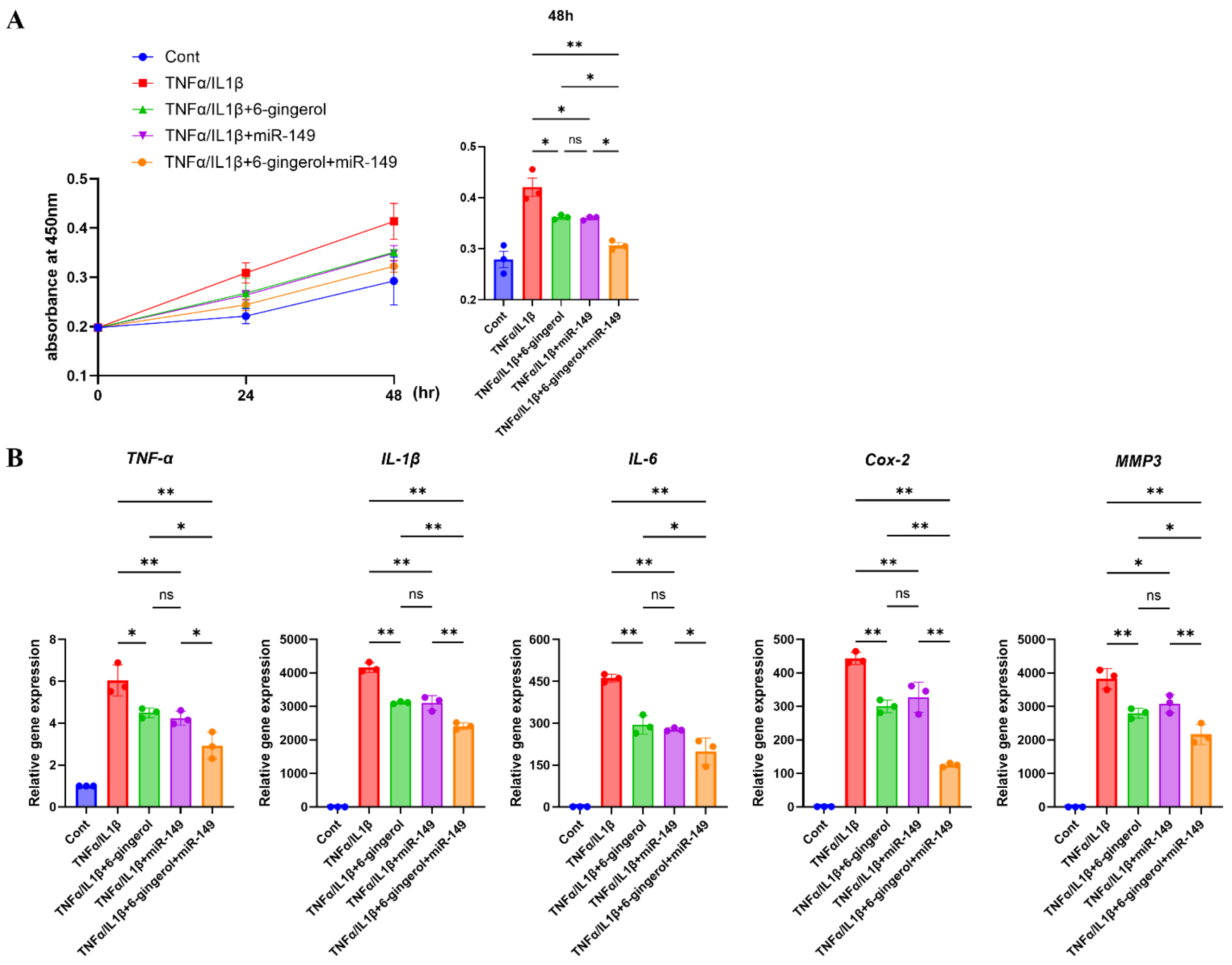

Figure S5. In vitro effects of combination treatment with miR-149 mimic and 6-gingerol on RASF. (A) RASFs were transfected with miR-149 mimic and subsequently treated with 6-gingerol. Cell viability was assessed using CCK-8 after 24 h (n = 3 per group). Compared with each single treatment, the combination of miR-149 mimic and 6-gingerol showed an enhanced suppressive effect on cell activation. (B) Gene expression analysis of inflammatory mediators was performed by qRT-PCR under the same conditions (n = 3 per group). The combination treatment resulted in greater suppression of inflammatory gene expression compared with either treatment alone. miR, microRNA; RASF, rheumatoid arthritis synovial fibroblasts; CCK-8, Cell Counting Kit-8; qRT-PCR, quantitative reverse-transcription polymerase chain reaction. **, $p < 0.01$; *, $p < 0.05$.

Supplemental Tables

Table S1: Representative plant-derived EV (PDEV) bioactive components and their therapeutic potential

Disease / Condition	Source of PDEVs	Key miRNAs / Components	Mechanism / Therapeutic Potential	Reference
Colitis	Ginger EVs	miR-7267-3p, bioactive lipids	Targets ycNE, increasing I3A and IL-22, leading to attenuation of colitis , and modulates gut microbiota	Suppl. Ref.1
Colitis	Ginger EVs	>125 miRNAs (unidentified individually)	Downregulates TNF-α, IL-6, IL-1β, and upregulates IL-10 and IL-22, providing protection against DSS-induced colitis	Suppl. Ref.2
Colitis	Ginger EVs	27 highly expressed miRNAs	Inhibits NF-κB, IL-6, IL-8, and TNF-α; uptake occurs via caveolin-mediated endocytosis and micropinocytosis	16
Intestinal barrier / inflammation	Broccoli EVs	Plant miRNAs, sulforaphane-containing lipids	Activates AMPK in dendritic cells, inducing tolerogenic DCs and improving DSS colitis	Suppl. Ref.3
Cancer (chemo-sensitization)	Grapefruit EVs	miR-17	Modulates MHC-I expression, enhancing chemo-sensitivity	Suppl. Ref.4
Rheumatoid arthritis	Milk EVs	miR-30a, miR-223, miR-92a	Downregulates IL-6, MCP-1, and IgG2a, ameliorating cartilage and bone inflammation	Suppl. Ref.5
Liver injury (hepatoprotection)	Ginger EVs	miRNAs + 6-gingerol / 6-shogaol	Reduces ALT and AST, decreases TG, and protects against alcohol-induced liver injury	Suppl. Ref.6

EV, Extracellular vesicle; miRNA/miR, microRNA; ycNE, yeast cell–derived nanovesicles; I3A, indole-3-aldehyde; IL, interleukin; DSS, dextran sulfate sodium; NF-κB, nuclear factor kappa-light-chain-enhancer of activated B cells; TNF-α, Tumor necrosis factor-alpha; AMPK, AMP-activated protein kinase; DCs, dendritic cells; MHC-I, major histocompatibility complex class I; MCP-1, monocyte chemoattractant protein-1; IgG2a, immunoglobulin G2a; ALT, alanine aminotransferase; AST, aspartate aminotransferase; TG, triglyceride; Suppl. Ref, Supplemental References

Table S2: Characterization of Ginger EVs

Parameter		ginger EV
Particle concentration	(particles/mL)	2.66×10^9
Median diameter	(nm)	239
Protein concentration	(mg/mL)	3.8

EV, Extracellular vesicle

Table S3: Highly Expressed miRNAs in Ginger EVs (Microarray Analysis)

No.	miRNA	Expression value	Mechanism / Target	Therapeutic Effect / Disease	Reference
1	hsa-miR-6087	15942.4	Regulates CCNA2, RFC3/4, BLM in hESC cardiomyogenesis via polysome association	Controls cell cycle during cardiomyocyte differentiation; tissue regeneration	Suppl. Ref. 7
2	hsa-miR-6088	14876.4	Regulated in glioma cells via ceRNA effect of lncRNA MEG3, affecting SMARCB1 expression	Tumor-suppressive role in glioma	Suppl. Ref. 8
3	hsa-miR-149-3p	4776.4	Inhibits HIPK2-mediated RASF proliferation, migration, invasion; modulates circ_0008410 axis	Anti-inflammatory; suppresses RASF dysfunction	34
			Inhibits NF-κB activation; downregulates TNF-α, IL-6, IL-1β	Anti-inflammatory; suppresses inflammation in RA	40
			Enhances AMPK, inhibits NF-κB; downregulated in IBD	Anti-inflammatory; ameliorates colitis; potential biomarker for IBD severity	Suppl. Ref. 9,10
4	hsa-miR-6800-5p	4485.4	Highly expressed in patient serum; biomarker with high diagnostic accuracy	Potential biomarker for ovarian cancer	Suppl. Ref. 11
5	hsa-miR-6085	4238.4	Not defined	Not defined	
6	hsa-miR-6089	3429.4	Targets TLR4, downregulating IL-6, IL-29, TNF-α in LPS-stimulated macrophages	Anti-inflammatory; potential biomarker/therapeutic target in rheumatoid arthritis	Suppl. Ref. 12
7	hsa-miR-3665	2825.4	Downregulated by promoter hypermethylation; MAPK/RAS pathway	Promoter hypermethylation linked to poor prognosis in esophageal squamous cell carcinoma	Suppl. Ref. 13
8	hsa-miR-6763-5p	2558.4	Significantly downregulated in plasma exosomes of epithelial ovarian cancer (EOC) patients	Potential non-invasive diagnostic biomarker for EOC	Suppl. Ref. 14
9	hsa-miR-4281	2345.4	Upregulated in fulminant myocarditis; inflammation-related	Potential circulating biomarker for fulminant myocarditis	Suppl. Ref. 15

10	hsa-miR-3188	2205.4	Targets mTOR, suppressing PI3K/AKT signaling	Tumor-suppressive; inhibits cell proliferation in non-small cell lung cancer	Suppl. Ref. 16
11	hsa-miR-3960	2006.4	Targets PHLDA2; inhibits IL-1β-mediated inflammation in chondrocytes	Anti-inflammatory; protects cartilage in osteoarthritis	Suppl. Ref. 17
12	hsa-miR-8072	1692.4	Not defined	Not defined	
13	hsa-miR-937-5p	1649.4	Targets IL-1β; suppresses TNF-α/IL-17 signaling in airway epithelial cells	Anti-inflammatory; protects airway epithelial cells post-CSE damage in COPD	Suppl. Ref. 18
14	hsa-miR-6511b-5p	1517.4	Upregulated in serum exosomes of RA patients in remission	Potential biomarker for RA disease activity	Suppl. Ref. 19
15	hsa-miR-5787	1401.4	Targets TLR4/NF-κB signaling in macrophages	Anti-inflammatory; inhibits macrophage proliferation and migration (cerebral infarction model)	Suppl. Ref. 20
16	hsa-miR-6090	1310.4	Not defined	Not defined	
17	hsa-miR-642a-3p	1257.4	Targets SERPINE1; promotes EMT, migration, and invasion	Tumor progression in hepatocellular carcinoma	Suppl. Ref. 21
			Protects β cells against glucolipotoxicity	β cell protection in diabetes	Suppl. Ref. 22
18	hsa-miR-4529-3p	1109.4	Targets RB1; activates ERK1/2 pathway	Promotes progression of retinoblastoma	Suppl. Ref. 23
19	hsa-miR-150-3p	1014.4	Targets Trim14; modulates NF-κB and IFN-β signaling	Suppresses cartilage degradation; protective effect in osteoarthritis	Suppl. Ref. 24

EV, Extracellular vesicle; hsa, Homo sapiens (human); miRNA/miR, microRNA; CCNA2, Cyclin A2; RFC3/4, Replication factor C subunit 3/4; BLM, Bloom syndrome RecQ-like helicase; hESC, Human embryonic stem cell; ceRNA, Competing endogenous RNA; lncRNA, Long non-coding RNA; MEG3, Maternally expressed gene 3; SMARCB1, SWI/SNF-related, matrix-associated, actin-dependent regulator of chromatin subfamily B member 1; HIPK2, Homeodomain-interacting protein kinase 2; RASF, Rheumatoid arthritis synovial fibroblasts; NF-κB, Nuclear factor kappa-light-chain-enhancer of activated B cells; TNF-α, Tumor necrosis factor-alpha; IL, Interleukin; AMPK, AMP-activated protein kinase; IBD, Inflammatory bowel disease; TLR4, Toll-like receptor 4; LPS, Lipopolysaccharide; MAPK, Mitogen-activated protein kinase; RAS, Rat sarcoma (proto-oncogene family); EOC, Epithelial ovarian cancer; mTOR, Mechanistic target of rapamycin; PI3K, Phosphoinositide 3-kinase; AKT, Protein kinase B; PHLDA2, Pleckstrin homology-like domain, family A, member 2; CSE, Cigarette smoke extract; COPD, Chronic obstructive pulmonary disease; SERPINE1, Serpin family E member 1 (plasminogen activator inhibitor-1, PAI-1); EMT, Epithelial-mesenchymal transition; RB1, Retinoblastoma protein; ERK1/2, Extracellular signal-regulated kinase 1/2; Trim14, Tripartite motif-containing protein 14; IFN-β, Interferon-beta; Suppl. Ref, Supplemental References

Table S4: Target Genes Based on Across-Study Analysis

Pathway	Target Genes	Functional relevance	Reference
Ras signaling	FLT4	Lymphangiogenesis, immune cell trafficking	[41]
	PLA2G2A	Eicosanoid production, pro-inflammatory mediator	[42]
	KSR1	MAPK scaffold, regulates ERK activity	[43]
	TTBK1	Neuroinflammation, immune modulation	[Suppl. Ref. 25]
MAPK cascade	IGFBP4	IGF signaling, cytokine regulation	[Suppl. Ref. 26]
	SOX9	Cartilage homeostasis, ECM maintenance	[Suppl. Ref. 27]
	DUSP19	MAPK phosphatase, fine-tunes inflammation	[44]

Ras, Rat sarcoma virus oncogene; MAPK, mitogen-activated protein kinase; FLT4, Fms related receptor tyrosine kinase 4; PLA2G2A, Phospholipase A2 group IIA; KSR1, Kinase suppressor of Ras 1; TTBK1, Tau tubulin kinase 1; IGFBP4, Insulin-like growth factor binding protein 4; SOX9, SRY-box transcription factor 9; DUSP19, Dual specificity phosphatase 19; ERK, extracellular signal–regulated kinase; ECM, extracellular matrix; Suppl. Ref, Supplemental References

Table S5: Ginger-derived Bioactive Components and Reported Therapeutic Effects

Compound	Reported therapeutic effects	References
6-Gingerol	Anti-inflammatory: Inhibits synovitis; reduces MMPs, TNF-α, IL-6; suppresses 5-LOX and prostaglandin synthase activity	[21]
	Anti-osteoclastogenic: Inhibits inflammation-associated osteoclast differentiation via reduction of PGE_2 levels	[23]
6-Shogaol	Anti-inflammatory: Inhibits synovitis; reduces proliferation and apoptosis of synovial tissue via PI3K/Akt/NF-κB signaling; effective in collagen-induced and carrageenan-induced arthritis models	[22, 25]
	Cartilage-protective: Inhibits chondrocyte innate immune responses and cathepsin-K activity, thereby reducing cartilage degradation.	[24]
8-Shogaol	Anti-inflammatory: Inhibits synovitis; suppresses TNF-α, IL-1β, IL-17-mediated inflammation and migration; improves arthritis in vivo (AIA rat model)	[19]
Zingerone	Anti-inflammatory (inhibits NF-κB/MAPK, reduces TNF-α, IL-1β, IL-6); Antioxidant (scavenges free radicals, prevents lipid peroxidation)	[Suppl. Ref. 28]
6-Paradol	Anti-inflammatory; inhibits proliferation and metastasis via EGFR/PI3K/AKT; reduces cytokines IL-6, TNF-α; antioxidant	[Suppl. Ref. 29]

MMP, matrix metalloproteinase; TNF-α, tumor necrosis factor-alpha; IL-6, interleukin-6; 5-LOX, 5-lipoxygenase; PGE_2, prostaglandin E_2; PI3K, phosphatidylinositol 3-kinase; Akt, protein kinase B; NF-κB, nuclear factor-kappa B; IL-1β, interleukin-1 beta; IL-17, interleukin-17; AIA, adjuvant-induced arthritis; MAPK, mitogen-activated protein kinase; EGFR, Epidermal Growth Factor Receptor; Suppl. Ref, Supplemental References

Table S6: Rheumatoid arthritis patient profiles

No.	Age (years)	Sex	BMI (kg/m^2)	Larsen grade *	Medicine	RF (IU/mL)	CRP (mg/dL)	ESR (mm/h)	Duration of disease (years)	Surgical treatment
1	57	F	18.6	3	Methotrexate, Bucillamine, Salazosulfapyridine, Prednisolone	21.5	0.96	37	7	TAA
2	73	F	23.5	4	Celecoxib, Salazosulfapyridine, Prednisolone, Tocilizumab	45.8	1.15	46	5	TAA
3	75	F	17.6	4	Methotrexate, Prednisolone, Etanercept	127.7	3.30	122	7	TKA
4	65	M	15.3	4	Loxoprofen, Methotrexate, Golimumab	6.7	4.11	78	15	TKA
5	58	M	24.7	3	Celecoxib, Prednisolone, Etanercept	170.5	0.67	9	27	THA

BMI, Body Mass Index; RF, Rheumatoid Factor; CRP, C-Reactive Protein; ESR, Erythrocyte Sedimentation Rate; TAA, Total Ankle Arthroplasty; TKA, Total Knee Arthroplasty; THA, Total Hip Arthroplasty; *, Supplemental reference [30]

Table S7. TaqMan Gene Expression Assay IDs for qRT-PCR

Gene	Target Name	Assay ID
TNF-α	Tumor necrosis factor-alpha	Hs00174128_m1
IL-1β	Interleukin-1 beta	Hs01555410_m1
IL-6	Interleukin-6	Hs00985639_m1
Cox-2	Cyclooxygenase-2	Hs00153133_m1
MMP3	Matrix metalloproteinase 3	Hs00233962_m1
GAPDH	Glyceraldehyde-3-phosphate dehydrogenase	Hs99999905_m1

qRT-PCR, quantitative reverse-transcription polymerase chain reaction.

Table S8. List of common miRNAs between MSCEVs and GDEVs

This table provides the list of 2,307 miRNAs commonly detected in MSCEVs and GDEVs by microarray analysis.

Due to the large dataset, this table is provided as a separate Excel file.

Supplemental Materials and Methods

Cell Proliferation Assay

To evaluate the appropriate concentration range of ginger-derived extracellular vesicles (GDEVs), Rheumatoid arthritis synovial fibroblasts (RASFs) were seeded in 96-well plates at a density of 3×10^3 cells/well in Dulbecco's modified Eagle medium (DMEM; high glucose, without L-glutamine and phenol red; FUJIFILM Wako Pure Chemical Corporation, Osaka, Japan) supplemented with 10% fetal bovine serum (FBS; Thermo Fisher Scientific, Waltham, MA, USA) and 1% penicillin–streptomycin–amphotericin B (AB; FUJIFILM Wako Pure Chemical Corporation, Osaka, Japan).

After overnight incubation, the following treatment groups were prepared: non-treated control and GDEVs at various concentrations (0.2X, 0.5X, 1X, 2X, 3X). After 24 h of treatment, CCK-8 reagent (Dojindo Laboratories, Kumamoto, Japan) was added to each well and incubated for 1 h.

Absorbance was measured at 450 nm using a microplate reader (Tecan Infinite 200 PRO, Tecan Group Ltd., Männedorf, Switzerland) at 0, 24, and 48 h to evaluate cell proliferation. This procedure was performed following the same methodology described in the Main Methods section.

Cytotoxicity Assay (LDH Release and Live/Dead Cell Staining)

Cytotoxicity induced by GDEVs was evaluated using a Lactate Dehydrogenase (LDH) release assay. RASFs were seeded in 24-well plates at 5×10^4 cells/well and treated with various concentrations of GDEVs for 24 h.

Culture supernatants (100 μL) were collected and mixed with reaction reagent from the Cytotoxicity LDH Assay Kit-WST (Dojindo Laboratories, Kumamoto, Japan) according to the manufacturer's protocol. After a 30-min incubation at room temperature in the dark, absorbance was measured at 490 nm.[31]

Live and dead cells were assessed using the Cellstain Double Staining Kit (Dojindo Laboratories, Kumamoto, Japan). After washing with phosphate-buffered saline (PBS), cells were incubated with calcein-AM (live cell indicator) and propidium iodide (dead cell indicator) diluted in PBS for 15 min at 37 °C.[32]

Fluorescent images were acquired using a fluorescence microscope (BZ-X710, Keyence Corporation, Osaka, Japan), and live/dead cells were counted using Keyence analysis software.

FAP immunofluorescence staining and quantification

Fibroblast activation protein-α (FAPα) is highly expressed in synovial fibroblasts of patients with rheumatoid arthritis (RA), where it has been implicated in synovial inflammation and joint destruction.[33] Based on this disease relevance, FAP expression was evaluated in our experimental model by immunofluorescence staining. Paraffin sections of EDTX-decalcified mouse joint tissue were deparaffinized and treated with Proteinase K (DAKO, S3020, Glostrup, Denmark) for antigen retrieval. After PBS washes, sections were blocked with 5% normal goat serum (Invitrogen, MA, USA), 1% BSA (Wako, Osaka, Japan), and 0.1% Triton X-100 (Sigma-Aldrich, MO, USA) in PBS. Sections were incubated overnight at 4 °C with the primary antibody against fibroblast activation protein-α (FAPα; Cloud-Clone, PAC469Mu01, TX, USA) or rabbit IgG isotype control (Invitrogen, MA, USA). After rinsing with PBS, sections were treated with ImmunoClear (Matsunami, Osaka, Japan), followed by incubation with Alexa Fluor 568-conjugated anti-rabbit IgG secondary antibody (Invitrogen, A-11011, MA, USA; 1:500) and DAPI (Dojindo Laboratories Co., Ltd., Kumamoto, Japan; 1:1000) for 1 h at room temperature. Finally, sections were treated with ImmunoClear, washed with PBS, and mounted with antifade medium (Fluoromount, Cosmo Bio, Tokyo, Japan).

Fluorescence images were acquired using a KEYENCE BZ-X710 fluorescence microscope (KEYENCE, Osaka, Japan) under identical acquisition settings. For each sample, five random regions of interest (ROIs; 0.04 mm^2 each) were selected within the synovial tissue, excluding non-synovial tissues such as bone and muscle using the KEYENCE BZ-X Analyzer. Quantitative analysis was performed using the KEYENCE BZ-X Analyzer, measuring total DAPI-stained nuclei and FAP-positive area. The mean values of the five ROIs per sample were used for statistical analysis.

Immunofluorescence staining of JAM-A to identify intestinal epithelial cells

Junctional adhesion molecule-A (JAM-A) is a tight junction protein specifically expressed in intestinal epithelial cells, and thus was used as a marker to identify epithelial regions.[34] Cryosections of mouse intestinal tissue were air-dried and rinsed in PBS. Sections were blocked with 5% normal goat serum (Invitrogen, MA, USA), 1% BSA (Wako, Osaka, Japan), and 0.1% Triton X-100 (Sigma-Aldrich, MO, USA) in PBS for 30 min at 37 °C. The primary antibody against JAM-A (Proteintech, 16183-1-AP, IL, USA; rabbit polyclonal, 1:100 dilution in blocking buffer) was applied overnight at 4 °C, following a previously described method.[35] The following day, sections were washed twice with ImmunoClear (Matsunami, Osaka, Japan) and three times with PBS. Secondary antibody (Alexa Fluor 488–conjugated donkey anti-rabbit IgG (H+L); Invitrogen, A32790, MA, USA; 1:500 dilution in blocking buffer) and DAPI (Dojindo Laboratories Co., Ltd., Kumamoto, Japan; 1:1000) were applied for 1 h at 37 °C. Finally, sections were washed with PBS and mounted with antifade medium (Fluoromount, Cosmo Bio, Tokyo, Japan).

Transfection of miR-149-3p mimic into RASF

Double-stranded RNA oligonucleotides representing the mature miR-149-3p sequence (sense: 5′-UCUGGCUCCGUGUCUUCACUCCC-3′ and antisense: 5′-GGGAGUGAAGACACGGAGCCAGA-3′) were synthesized by Hokkaido System Science Co., Ltd. (Sapporo, Japan). A final concentration of 10 nM miR-149-3p mimic or a negative control RNA (Silencer Negative Control siRNA #1, Thermo Fisher Scientific, Waltham, MA, USA) was transfected into rheumatoid arthritis synovial fibroblasts (RASF) using Lipofectamine RNAiMAX reagent (Thermo Fisher Scientific, Waltham, MA, USA) according to the manufacturer's instructions.[36] The concentration of 10 nM was selected based on our preliminary experiments showing stable transfection efficiency and minimal cytotoxicity at 1, 5, and 10 nM, as well as previously reported studies employing similar concentrations for miR-149-3p transfection in cultured cells.[37] After 24 h of incubation, transfected cells were used for the subsequent analyses.

Combination Treatment of miR-149 and 6-Gingerol

To evaluate the combinatory effects of miR-149 and 6-gingerol, RASFs were assigned to three groups: (1) miR-149 only, (2) 6-gingerol only, and (3) miR-149 + 6-gingerol (combined treatment). miR-149 mimic transfection and 6-gingerol administration were performed as described in the main Methods. After treatment under inflammatory stimulation (TNFα 5 ng/mL and IL-1β 5 ng/mL), cell proliferation was assessed by CCK-8 assay, and inflammatory gene expression (TNFα, IL-1β, IL-6, COX-2, MMP3) was analyzed by qRT-PCR following standard procedures described above. This procedure was performed following the same methodology described in the Main Methods section.

Supplemental References

1. Teng, Y., Ren, Y., Sayed, M., Hu, X., Lei, C., Kumar, A., Hutchins, E., Mu, J., Deng, Z., Luo, C., et al. (2018). Plant-Derived Exosomal MicroRNAs Shape the Gut Microbiota. Cell Host Microbe *24*, 637-652 e638.
2. Zhang, M., Viennois, E., Prasad, M., Zhang, Y., Wang, L., Zhang, Z., Han, M.K., Xiao, B., Xu, C., Srinivasan, S., et al. (2016). Edible ginger-derived nanoparticles: A novel therapeutic approach for the prevention and treatment of inflammatory bowel disease and colitis-associated cancer. Biomaterials *101*, 321-340.
3. Deng, Z., Rong, Y., Teng, Y., Mu, J., Zhuang, X., Tseng, M., Samykutty, A., Zhang, L., Yan, J., Miller, D., et al. (2017). Broccoli-Derived Nanoparticle Inhibits Mouse Colitis by Activating Dendritic Cell AMP-Activated Protein Kinase. Mol Ther *25*, 1641-1654.
4. Zhuang, X., Teng, Y., Samykutty, A., Mu, J., Deng, Z., Zhang, L., Cao, P., Rong, Y., Yan, J., Miller, D., et al. (2016). Grapefruit-derived Nanovectors Delivering Therapeutic miR17 Through an Intranasal Route Inhibit Brain Tumor Progression. Mol Ther *24*, 96-105.
5. Arntz, O.J., Pieters, B.C., Oliveira, M.C., Broeren, M.G., Bennink, M.B., de Vries, M., van Lent, P.L., Koenders, M.I., van den Berg, W.B., van der Kraan, P.M., et al. (2015). Oral administration of bovine milk derived extracellular vesicles attenuates arthritis in two mouse models. Mol Nutr Food Res *59*, 1701-1712.
6. Zhuang, X., Deng, Z.B., Mu, J., Zhang, L., Yan, J., Miller, D., Feng, W., McClain, C.J., and Zhang, H.G. (2015). Ginger-derived nanoparticles protect against alcohol-induced liver damage. J Extracell Vesicles *4*, 28713.
7. Machado, H.C., Bispo, S., and Dallagiovanna, B. (2023). miR-6087 Might Regulate Cell Cycle-Related mRNAs During Cardiomyogenesis of hESCs. Bioinform Biol Insights *17*, 11779322231161918.
8. Gong, X. and Huang, M.Y. (2020). Tumor-Suppressive Function of lncRNA-MEG3 in Glioma Cells by Regulating miR-6088/SMARCB1 Axis. Biomed Res Int *2020*, 4309161.
9. Feng, Q., Li, Y., Zhang, H., Wang, Z., Nie, X., Yao, D., Han, L., Chen, W.D., and Wang, Y.D. (2022). Deficiency of miRNA-149-3p shaped gut microbiota and enhanced dextran sulfate sodium-induced colitis. Mol Ther Nucleic Acids *30*, 208-225.
10. Luo, S. and Chen, X.H. (2024). Tissue and serum miR-149-3p/5p in hospitalized patients with inflammatory bowel disease: Correlation with disease severity and inflammatory markers. Kaohsiung J Med Sci *40*, 131-138.
11. Hamidi, F., Gilani, N., Arabi Belaghi, R., Yaghoobi, H., Babaei, E., Sarbakhsh, P., and Malakouti, J. (2023). Identifying potential circulating miRNA biomarkers for the diagnosis and prediction of ovarian cancer using machine-learning approach: application of Boruta. Front Digit Health *5*, 1187578.
12. Xu, D., Song, M., Chai, C., Wang, J., Jin, C., Wang, X., Cheng, M., and Yan, S. (2019). Exosome-encapsulated miR-6089 regulates inflammatory response via targeting TLR4. J Cell Physiol *234*, 1502-1511.
13. Zhou, J., Liu, S., Zhang, J., Zeng, Q., Lin, Z., Fu, R., Lin, Y., and Hu, Z. (2025). Discovery and validation of Hsa-microRNA-3665 promoter methylation as a potential biomarker for the prognosis of esophageal squaous cell carcinoma. Int J Clin Oncol *30*, 309-319.
14. Wang, S., Song, X., Wang, K., Zheng, B., Lin, Q., Yu, M., Xie, L., Chen, L., and Song, X. (2022). Plasma

exosomal miR-320d, miR-4479, and miR-6763-5p as diagnostic biomarkers in epithelial ovarian cancer. Front Oncol *12*, 986343.

15. Nie, X., He, M., Wang, J., Chen, P., Wang, F., Lai, J., Li, C., Yu, T., Zuo, H., Cui, G., et al. (2020). Circulating miR-4763-3p Is a Novel Potential Biomarker Candidate for Human Adult Fulminant Myocarditis. Mol Ther Methods Clin Dev *17*, 1079-1087.
16. Wang, C., Liu, E., Li, W., Cui, J., and Li, T. (2018). MiR-3188 Inhibits Non-small Cell Lung Cancer Cell Proliferation Through FOXO1-Mediated mTOR-p-PI3K/AKT-c-JUN Signaling Pathway. Front Pharmacol *9*, 1362.
17. Ye, P., Mi, Z., Wei, D., Gao, P., Ma, M., and Yang, H. (2022). miR-3960 from Mesenchymal Stem Cell-Derived Extracellular Vesicles Inactivates SDC1/Wnt/beta-Catenin Axis to Relieve Chondrocyte Injury in Osteoarthritis by Targeting PHLDA2. Stem Cells Int *2022*, 9455152.
18. Liu, T. (2021). miR-937 serves as an inflammatory inhibitor in cigarette smoke extract-induced human bronchial epithelial cells by targeting IL1B and regulating TNF-alpha/IL-17 signaling pathway. Tob Induc Dis *19*, 55.
19. Lim, M.K., Yoo, J., Sheen, D.H., Ihm, C., Lee, S.K., and Kim, S.A. (2020). Serum Exosomal miRNA-1915-3p Is Correlated With Disease Activity of Korean Rheumatoid Arthritis. In Vivo *34*, 2941-2945.
20. Bao, Z., Zhang, S., and Li, X. (2021). MiR-5787 Attenuates Macrophages-Mediated Inflammation by Targeting TLR4/NF-kappaB in Ischemic Cerebral Infarction. Neuromolecular Med *23*, 363-370.
21. Zhang, S., Cao, G., Shen, S., Wu, Y., Tan, X., and Jiang, X. (2024). CAF-derived miR-642a-3p supports migration, invasion, and EMT of hepatocellular carcinoma cells by targeting SERPINE1. PeerJ *12*, e18428.
22. Pinhancos, S.S., Teixeira de Oliveira, J., Alves, C.H., Deus, C.M., de Winter, T.J.J., Viana, S., Reis, F., Santos, J., Buitinga, M., Carlotti, F., et al. (2025). miRNA-642a-3p protects beta cells from glucolipotoxicity. Mol Ther Nucleic Acids *36*, 102498.
23. Gao, Y. and Du, P. (2024). miR-4529-3p Promotes the Progression of Retinoblastoma by Inhibiting RB1 Expression and Activating the ERK Signaling Pathway. Mol Biotechnol *66*, 102-111.
24. Wang, H., Shu, J., Zhang, C., Wang, Y., Shi, R., Yang, F., and Tang, X. (2022). Extracellular Vesicle-Mediated miR-150-3p Delivery in Joint Homeostasis: A Potential Treatment for Osteoarthritis? Cells *11*.
25. Dillon, G.M., Henderson, J.L., Bao, C., Joyce, J.A., Calhoun, M., Amaral, B., King, K.W., Bajrami, B., and Rabah, D. (2020). Acute inhibition of the CNS-specific kinase TTBK1 significantly lowers tau phosphorylation at several disease relevant sites. PLoS One *15*, e0228771.
26. Bayati, P., Taherian, M., and Mojtabavi, N. (2024). Immunomodulatory effects of the induced pluripotent stem cells through expressing IGF-related factors and IL-10 in vitro. Int J Immunopathol Pharmacol *38*, 3946320241276899.
27. Haseeb, A., Kc, R., Angelozzi, M., de Charleroy, C., Rux, D., Tower, R.J., Yao, L., Pellegrino da Silva, R., Pacifici, M., Qin, L., et al. (2021). SOX9 keeps growth plates and articular cartilage healthy by inhibiting chondrocyte dedifferentiation/osteoblastic redifferentiation. Proc Natl Acad Sci U S A *118*.
28. Ahmad, B., Rehman, M.U., Amin, I., Arif, A., Rasool, S., Bhat, S.A., Afzal, I., Hussain, I., Bilal, S., and Mir, M. (2015). A Review on Pharmacological Properties of Zingerone (4-(4-Hydroxy-3-methoxyphenyl)-2-butanone).

ScientificWorldJournal *2015*, 816364.

29. Jiang, X., Wang, J., Chen, P., He, Z., Xu, J., Chen, Y., Liu, X., and Jiang, J. (2021). [6]-Paradol suppresses proliferation and metastases of pancreatic cancer by decreasing EGFR and inactivating PI3K/AKT signaling. Cancer Cell Int *21*, 420.
30. Larsen, A., Dale, K., and Eek, M. (1977). Radiographic evaluation of rheumatoid arthritis and related conditions by standard reference films. Acta Radiol Diagn (Stockh) *18*, 481-491.
31. Takeda, Y. and Dai, P. (2022). Capsaicin directly promotes adipocyte browning in the chemical compound-induced brown adipocytes converted from human dermal fibroblasts. Sci Rep *12*, 6612.
32. Zhu, L., Yang, J., Zhang, J., and Peng, B. (2014). A comparative study of BioAggregate and ProRoot MTA on adhesion, migration, and attachment of human dental pulp cells. J Endod *40*, 1118-1123.
33. Bauer, S., Jendro, M.C., Wadle, A., Kleber, S., Stenner, F., Dinser, R., Reich, A., Faccin, E., Godde, S., Dinges, H., et al. (2006). Fibroblast activation protein is expressed by rheumatoid myofibroblast-like synoviocytes. Arthritis Res Ther *8*, R171.
34. Wong, E.A. and Kinstler, S.R. (2023). Research Note: Junctional adhesion molecule A is expressed in epithelial cells of the crypt and villi whereas junctional adhesion molecule 2 is expressed in vascular cells. Poult Sci *102*, 102693.
35. Li, Y., Peng, L., Cao, X., Yang, K., Wang, Z., Xiao, Y., Xiao, H., Qian, C., and Liu, H. (2022). The Long Non-Coding RNA HOXC-AS3 Promotes Glioma Progression by Sponging miR-216 to Regulate F11R Expression. Front Oncol *12*, 845009.
36. Sumimoto, Y., Harada, Y., Yimiti, D., Watanabe, C., Miyaki, S., and Adachi, N. (2024). MicroRNA-26a deficiency attenuates the severity of frozen shoulder in a mouse immobilization model. J Orthop Res *42*, 2623-2633.
37. Jin, L., Li, Y., Liu, J., Yang, S., Gui, Y., Mao, X., Nie, G., and Lai, Y. (2016). Tumor suppressor miR-149-5p is associated with cellular migration, proliferation and apoptosis in renal cell carcinoma. Mol Med Rep *13*, 5386-5392.
