# Supplementary material for: Oral ginger-derived extracellular vesicles ameliorate arthritis via anti-inflammatory actions of microRNA-149 and 6-gingerol
Source: Mol Ther Nucleic Acids. 2026 Jan 19;37(1):102840. doi: 10.1016/j.omtn.2026.102840 (PMC12887272; doi:10.1016/j.omtn.2026.102840)
Supplement: Document S2. Article plus supplemental information [file mmc3.pdf]

Molecular Therapy
Nucleic Acids
Original Article

Oral ginger-derived extracellular vesicles ameliorate arthritis via anti-inflammatory actions of microRNA-149 and 6-gingerol

Hiroki Kaneta,[1] Tomoyuki Nakasa,[2] Dilimulati Yimiti,[1] Dan Moriwaki,[1] Riku Kawasaki,[3] Toshihiko Ogura,[4] Shigeru Miyaki,[1,5] and Nobuo Adachi[1]

[1]Department of Orthopaedic Surgery, Graduate School of Biomedical and Health Sciences, Hiroshima University, 1-2-3 Kasumi, Minami-ku, Hiroshima 734-8551, Japan; [2]Department of Artificial Joints and Biomaterials, Graduate School of Biomedical and Health Sciences, Hiroshima University, 1-2-3 Kasumi, Minami-ku, Hiroshima 734-8551, Japan; [3]Program of Applied Chemistry, Graduate School of Advanced Science and Engineering, 1-4-1 Kagamiyama, Higashi-Hiroshima, Japan; [4]National Institute of Advanced Industrial Science and Technology (AIST), Central 6, Higashi, Tsukuba, Ibaraki 305-8566, Japan; [5]Medical Center for Translational and Clinical Research, Hiroshima University Hospital, 1-2-3 Kasumi, Minami-ku, Hiroshima 734-8551, Japan

Ginger-derived extracellular vesicles (GDEVs) have emerged as a novel anti-inflammatory agent with advantages such as oral bioavailability, natural origin, and cost-effective large-scale production. This study evaluated the therapeutic potential of GDEVs in rheumatoid arthritis (RA), a chronic autoimmune disease characterized by synovial inflammation and joint destruction. We conducted both *in vitro* and *in vivo* experiments using synovial fibroblasts derived from RA patients and a collagen antibody-induced arthritis (CAIA) mouse model. *In vitro*, GDEVs significantly suppressed the expression of pro-inflammatory cytokines tumor necrosis factor-α (TNF-α) and interleukin (IL)-1β and downstream mediators IL-6, Cox-2, and matrix metalloproteinase 3 (MMP3) and inhibited the proliferation and migration of RA synovial fibroblasts. *In vivo*, oral administration of GDEVs to CAIA mice reduced arthritis severity, attenuated synovitis, preserved cartilage integrity, and suppressed osteoclast activation. GDEVs were stable against gastric digestion and were efficiently taken up by intestinal cells, supporting their oral availability. Microarray and RNA sequencing identified miR-149 as a key regulatory molecule in GDEVs, associated with the suppression of inflammation-related signaling pathways, including Ras signaling and mitogen-activated protein kinase (MAPK) cascades. These findings highlight the potential of GDEVs as an anti-inflammatory therapy for RA. Given their stability and bioavailability, the oral administration of GDEVs could be a promising non-invasive treatment for future clinical applications.

INTRODUCTION

Rheumatoid arthritis (RA) is a chronic autoimmune disease characterized by persistent synovial inflammation leading to joint destruction and functional impairment.[1,2] The primary pathological features of RA include hyperplasia of synovial fibroblasts, excessive production of proinflammatory cytokines, and recruitment of immune cells, all of which contribute to cartilage degradation and bone erosion.[3] Uncontrolled RA can result in musculoskeletal deformities, impaired functionality, and high morbidity rates. Additionally, RA is associated with various extra-articular manifestations, including cardiovascular complications, due to its chronic systemic inflammatory and autoimmune nature.[4,5] Modern therapeutic strategies for managing RA include the use of nonsteroidal anti-inflammatory drugs (NSAIDs), disease-modifying antirheumatic drugs (DMARDs), glucocorticoids, biologics, and Janus kinase inhibitors (JAKs). Although these treatments effectively reduce the symptoms and decelerate disease progression, they have notable limitations.[6] These therapies are often associated with high costs and adverse effects, such as immunosuppression and increased infection risk, and require frequent administration, either through injections or prolonged use, which can lead to poor patient compliance and an increased risk of systemic complications.[7,8] Therefore, the development of an effective and safe oral therapeutic strategy remains an urgent challenge in the management of RA.

Extracellular vesicles (EVs) have emerged as potential therapeutic agents because of their ability to mediate intercellular communication by delivering bioactive molecules, such as microRNAs (miRNAs) and messenger RNAs (mRNAs).[9] Recent studies have highlighted the therapeutic potential of EVs in modulating immune responses and promoting tissue repair.[10,11] However, the clinical application of EV therapy is hindered by challenges, such as high production costs, stability issues, and delivery inefficiencies.[12] To overcome these limitations, plant-derived EVs (PDEVs) have been investigated as promising alternatives that offer advantages in terms of their stability, scalability, and cost-effectiveness.[13] Several studies

Received 22 May 2025; accepted 14 January 2026;
https://doi.org/10.1016/j.omtn.2026.102840.

Corresponding author:
E-mail: tnakasa0@gmail.com

© 2026 The Authors.
Published by Elsevier Inc. on behalf of The American Society of Gene and Cell Therapy.
This is an open access article under the CC BY license (http://creativecommons.org/licenses/by/4.0/).

have shown that PDEVs are taken up by mammalian cells and exert immunomodulatory effects, indicating their potential role in inflammatory disease treatment.[14,15] Furthermore, PDEVs have been shown to carry various bioactive molecules, including miRNAs, lipids, and metabolites, which contribute to their immunomodulatory and therapeutic effects in diverse diseases (e.g., colitis, liver injury, and cancer).[16] These components can modulate inflammatory signaling pathways, enhance tissue repair, and influence immune cell functions, as summarized in Table S1.

Ginger (*Zingiber officinale*) is a widely used medicinal plant known for its potent anti-inflammatory properties.[17,18] Ginger and its bioactive components have been reported to alleviate RA-related symptoms and may contribute to the suppression of disease progression in preclinical models.[19,20] 6-Gingerol, a phenolic compound, has been shown to inhibit the production of inflammatory mediators such as tumor necrosis factor-α (TNF-α) and interleukin (IL)-1β by suppressing 5-lipoxygenase and prostaglandin synthase activity.[21] Meanwhile, 6-shogaol has demonstrated the ability to inhibit the proliferation and migration of RA synovial fibroblasts (RASFs) via modulation of the phosphoinositide 3-kinase/protein kinase B and nuclear factor kappa-light-chain-enhancer of activated B cells (NF-κB) signaling pathways.[22] Both compounds also reduce the expression of matrix metalloproteinases (MMPs), which are key contributors to cartilage degradation.[22–24] Their effects have been validated in preclinical models of arthritis, including collagen- and carrageenan-induced arthritis, where they significantly reduced joint swelling, synovial inflammation, and histological damage.[22,25] However, despite these promising findings, the direct application of ginger-derived bioactive compounds such as 6-gingerol faces several challenges. These compounds have limited bioavailability due to their rapid metabolism and poor solubility, which may reduce their therapeutic efficacy when administered orally or systemically.[18] In addition, their potential off-target effects and cytotoxicity at high concentrations raise concerns regarding their long-term safety.[26] Given these limitations, an alternative approach is required to optimize the therapeutic benefits of ginger-derived bioactive molecules while minimizing their adverse effects. Ginger has a long history of medicinal use, particularly in traditional medicine systems, for the treatment of inflammatory conditions. Accordingly, given that RA is a chronic inflammatory disease, the strong evidence supporting ginger's ability to inhibit key RA-related processes—including synovial hyperplasia, cytokine overproduction, and cartilage degradation—makes it a rational candidate for EV-based exploration. Moreover, prior studies have demonstrated that the anti-inflammatory effects of ginger are mediated through bioactive molecules that can be packaged into EVs, suggesting that ginger-derived extracellular vesicles (GDEVs) could serve as a natural delivery platform for these agents. In this context, GDEVs have emerged as a promising solution to address these challenges. GDEVs encapsulate and protect bioactive molecules, enhancing their stability and bioavailability.[27] Furthermore, EVs facilitate targeted delivery to immune cells and inflamed tissues, potentially improving therapeutic outcomes and reducing systemic side effects.[28]

Despite these advantages, the role of GDEVs in RA remains largely unknown. Given the potential to deliver bioactive miRNAs and modulate inflammatory pathways, we hypothesized that GDEVs could serve as a novel therapeutic approach for RA. This study aimed to investigate the efficacy of GDEVs in the treatment of RA using both *in vitro* and *in vivo* models and provide new insights into the potential of PDEVs as next-generation anti-inflammatory therapies. Furthermore, as GDEVs can be orally administered and naturally absorbed through the gastrointestinal tract, they represent a noninvasive and potentially better-tolerated alternative, especially compared to some current treatments such as JAKs.

RESULTS

Characterization of ginger EVs

GDEVs were successfully isolated from ginger extract by differential centrifugation and ultracentrifugation, followed by filtration. Electron microscopy using a scanning electron-assisted dielectric microscopy (SE-ADM) system revealed that the isolated vesicles were predominantly in the exosome size range (100–200 nm), with some larger vesicles exceeding 200 nm (Figure 1A). Nanoparticle tracking analysis (VideoDrop, Meiwafosis, Japan) confirmed a median particle diameter of 239 nm and a concentration of 2.66×10^9 particles/mL (Figure 1B, Table S2). The total protein concentration of GDEVs, measured using the Qubit Protein Assay (Qubit 2.0 Fluorometer, Invitrogen, USA), was 3.80 mg/mL (Table S2). Based on the yield calculation, approximately 47.5 mg of GDEVs were obtained per 1 kg of fresh ginger. These results are consistent with previous reports,[29–31] supporting the successful isolation of GDEV.

In vitro effects of GDEVs

To investigate the anti-inflammatory and anti-proliferative effects of GDEVs, we conducted a series of *in vitro* experiments using RASFs derived from human RA patients.

Cell proliferation assays were performed using Cell Counting Kit-8 (CCK-8; Dojindo Laboratories, Kumamoto, Japan) at 24 and 48 h after inflammatory stimulation with TNFα and IL-1β. Compared to the inflammation-only group, treatment with GDEVs (1X) significantly reduced cell proliferation at 48 h by approximately 20% (Figure 2A). To evaluate inflammatory and catabolic gene expression, quantitative reverse-transcription polymerase chain reaction (RT-qPCR) was performed after 24-h treatment with GDEVs under inflammatory conditions. Compared to the inflammation-only group, GDEVs (1X) significantly suppressed mRNA expression of TNFα (35.1%) and IL-1β (23.2%), as well as downstream mediators IL-6 (50.4%) and cyclooxygenase-2 (COX-2) (30.7%). The catabolic enzyme MMP3 was also markedly reduced by 52.2% in GDEV-treated RASFs (Figure 2B). These genes are known to be involved in matrix degradation and inflammation in RA pathogenesis. After the analyses shown in Figure 2, we further evaluated the effects of GDEVs on cell proliferation and cytotoxicity to determine the optimal concentration for subsequent experiments. Cell proliferation was assessed using CCK-8, and cytotoxicity was examined by lactate dehydrogenase (LDH) release and Live/Dead staining

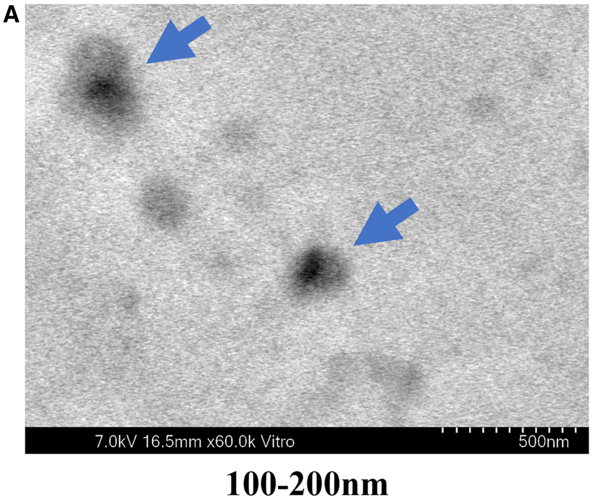

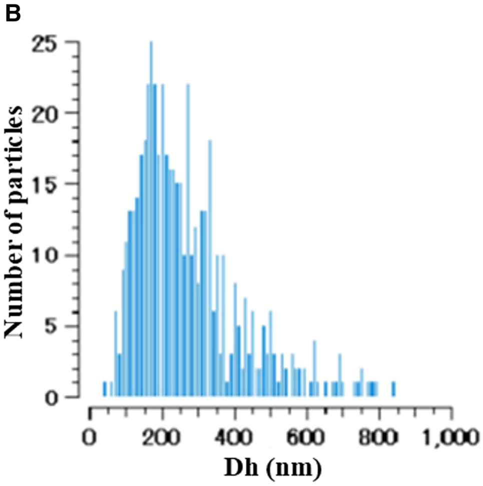

Figure 1. Characterization of GDEVs

(A) SE-ADM images showing round vesicles with diameters of 100–200 nm (blue arrows indicate GDEVs). (B) Nanoparticle tracking analysis showing the particle size distribution and relative concentration of GDEVs. Dh, hydrodynamic diameter.

(Figure S1). Higher concentrations of GDEVs (2X and 3X) showed a stronger inhibition of cell proliferation compared with the Control (Figure S1A). However, these concentrations also induced increased LDH release (Figure S1B), indicating cytotoxicity, and Live/Dead staining confirmed evident cell death at 2X and 3X (Figures S1C and S1D). In addition, concentrations above 2X induced cell detachment, further suggesting potential cytotoxicity. Based on these initial dose-response findings, 1X was selected as the maximum non-cytotoxic concentration for subsequent experiments. Based on the results shown in Figures 2A, 2B, and S1, the concentration corresponding to the green line (GDEV 1X) was selected as the standard dose for subsequent experiments. At this concentration, both cell proliferation and inflammatory gene expression were significantly suppressed compared with lower doses (0.2X), whereas increasing the dose did not provide additional benefits. Moreover, concentrations above 2X induced cell detachment, suggesting potential cytotoxicity. Therefore, GDEV 1X was considered the most effective and stable concentration for *in vitro* assays. Additionally, the scratch assay was used to assess cell migration. RASFs were either left untreated (Control) or treated with GDEVs (1X) under inflammatory conditions. Compared to the untreated RASFs, GDEV-treated cells showed significantly reduced migration over 24 h, with an approximate 52.1% decrease in the migrated area relative to the control group, as evidenced by slower closure of the scratched area (Figure 2C).

These data collectively demonstrate that GDEVs suppress inflammatory activation, proliferation, and migration of human RASFs *in vitro*.

In vivo effects of GDEVs

Oral GDEVs were evaluated for their therapeutic efficacy in a collagen antibody-induced arthritis (CAIA) mouse model (Figure 3A). Mice treated with oral GDEVs exhibited consistently lower arthritis scores compared to the control group from days 4 to 10, as assessed by a standardized clinical scoring system based on joint swelling and redness (Figure 3B, $p < 0.05$). Locomotor behavior was analyzed using an open-field test on day 10. GDEV-treated mice exhibited a greater total distance traveled (Control: 1,926.5 ± 550.8 mm, GDEV: 2,935.9 ± 644.5 mm, $p < 0.01$) and reduced resting time (Control: 320.6 ± 83.8 s, GDEV: 208.9 ± 56.0 s, $p < 0.01$), indicating improved mobility and reduced pain-related behavior (Figure 3C).

Histological analyses were performed on ankle joints collected at euthanasia. Hematoxylin and eosin (H&E) staining revealed that GDEV treatment significantly reduced synovial inflammation, with lower synovitis scores compared to the control group (Control: 5.5 ± 0.7, GDEV: 4.1 ± 1.4, $p < 0.05$). Safranin O-fast green staining demonstrated preservation of proteoglycan content in cartilage, as reflected by higher safranin O scores in the GDEV group (Control: 4.3 ± 1.1, GDEV: 2.7 ± 1.3, $p < 0.05$). Additionally, tartrate-resistant acid phosphatase (TRAP) staining revealed fewer TRAP-positive osteoclasts in GDEV-treated mice (Control: 38.5 ± 7.9, GDEV: 22.3 ± 7.6, $p < 0.01$), indicating suppressed bone resorption activity (Figure 3D). To further investigate fibroblast activation within the synovium, we performed fibroblast activation protein (FAP) immunofluorescence staining. The total number of 4′,6-diamidino-2-phenylindole $(DAPI)^+$ nuclei did not differ significantly between groups (Control: 290.9 ± 118.3, GDEV: 284.3 ± 94.6). In contrast, the FAP-positive area (Control: 8,659.6 ± 7,482.1, GDEV: 2,611.0 ± 1,877.6, $p < 0.05$) was significantly reduced in the GDEV group (Figure S2).

To evaluate systemic safety, serum biomarkers—aspartate aminotransferase (AST), alanine aminotransferase (ALT), blood urea nitrogen (BUN), creatinine (CRE), and amylase (AMY)—which are

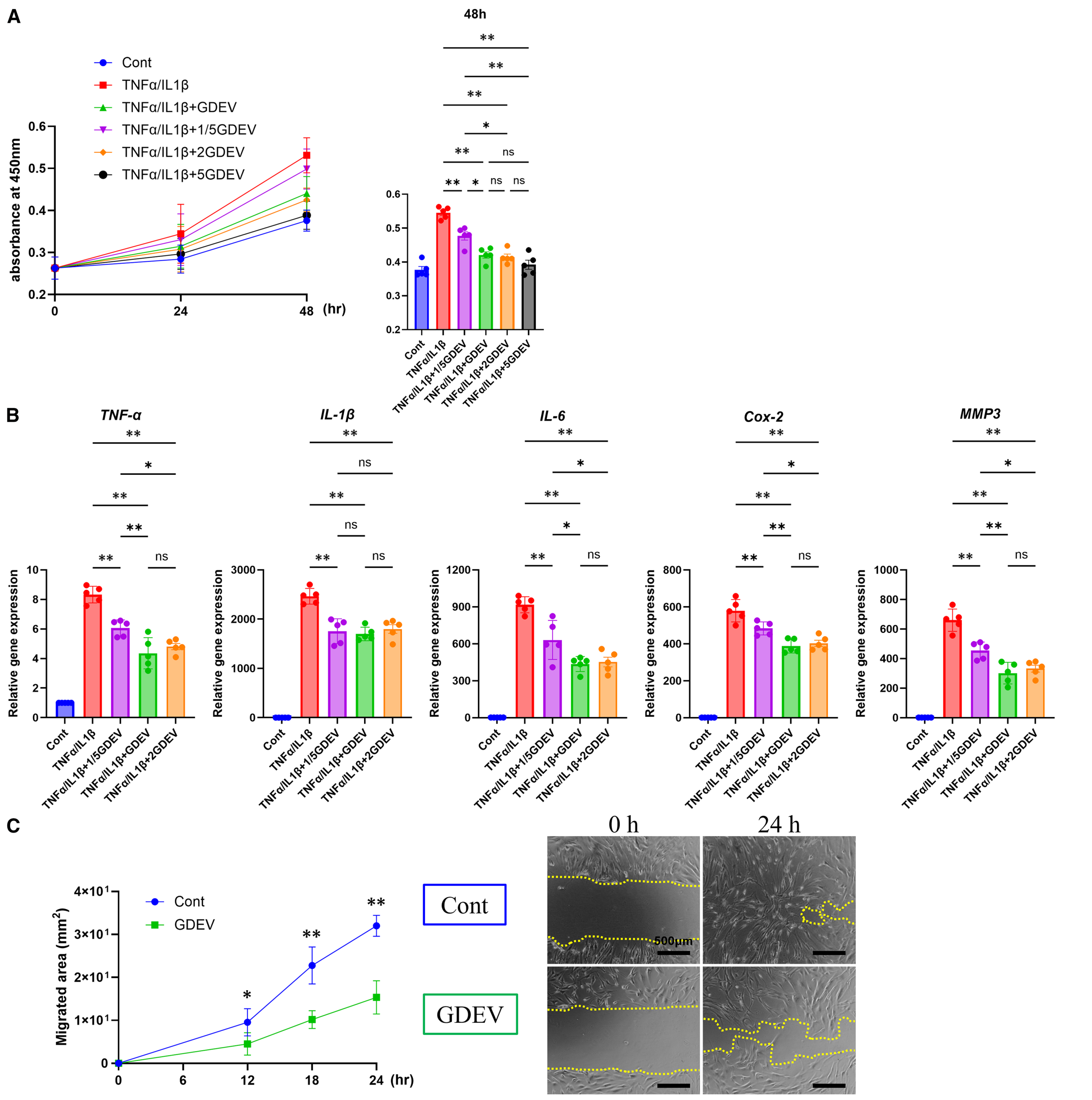

Figure 2. *In vitro* effects of GDEVs on RASF

(A) Cell viability of RASF measured by MTT assay at 48 h. Six groups were analyzed: Cont, TNFα/IL1β, TNFα/IL1β + GDEV (0.2X), TNFα/IL1β + GDEV (1X), TNFα/IL1β + GDEV (2X), and TNFα/IL1β + GDEV (5X) ($n = 5$ per group). GDEVs dose dependently reduced RASF proliferation. (B) Gene expression analysis of inflammatory mediators in RASF treated with GDEVs (0.2X, 1X, 2X) under TNFα/IL1β stimulation ($n = 5$ per group). mRNA levels of TNFα, IL-1β, IL-6, COX-2, and MMP3 were measured by RT-qPCR. GDEVs significantly suppressed the expression of these inflammatory genes. (C) Scratch assay evaluating cell migration over 12, 18, and 24 h. Two groups were compared: Cont and TNFα/IL1β + GDEV (1X) ($n = 5$ per group). Representative images at 0 and 24 h are shown. GDEVs inhibited RASF migration. Cont, control. $^{**}p < 0.01$, $^{*}p < 0.05$.

widely recognized indicators of liver, kidney, and pancreatic function in preclinical toxicity studies,[32] were analyzed, and body weights were monitored throughout the experiment. Compared to the control group, the GDEV-treated mice showed no significant alterations in serum parameters: AST (−21.7%), ALT (−14.7%), BUN (−11.0%), CRE (−14.6%), and AMY (−15.7%) (Figure 3E). Body

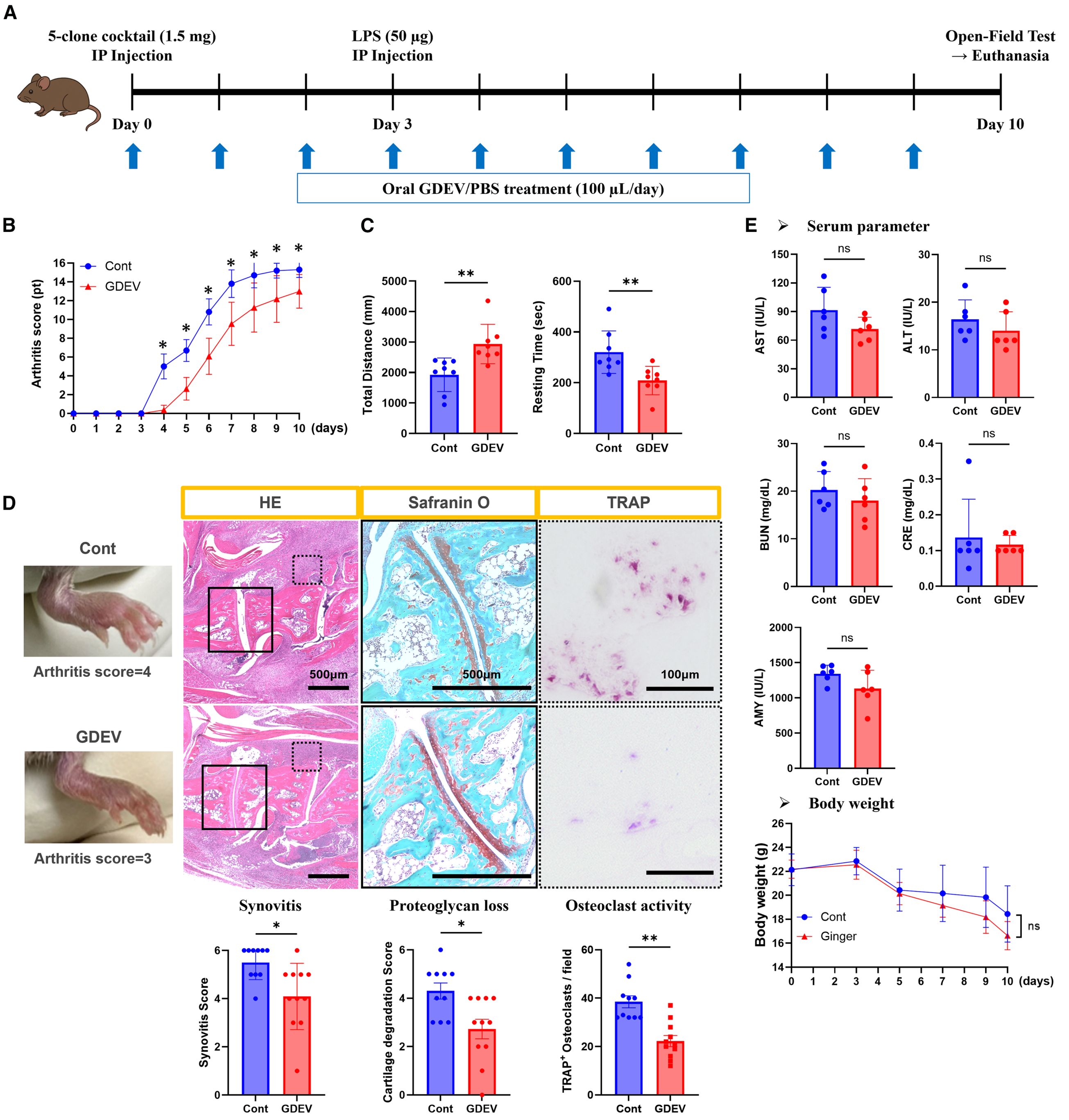

Figure 3. *In vivo* therapeutic effects of GDEVs in CAIA mice

(A) Experimental protocol for CAIA induction and GDEV treatment. (B) Arthritis scores were assessed over time. Two groups were analyzed: Cont (n = 10) and GDEV-treated mice (GDEV, n = 11). GDEV treatment reduced arthritis severity. (C) Open-field test measuring total distance traveled and resting time at endpoint. Two groups: Cont (n = 8) and GDEV (n = 8). GDEVs improved locomotor activity and reduced resting time. (D) Representative macroscopic images of joint swelling and histological analysis. Synovitis (H&E staining), proteoglycan loss (safranin-O staining), and osteoclast activity (TRAP staining) were evaluated. Synovitis, cartilage degradation, and TRAP$^+$ osteoclast scores are shown (Cont, n = 10; GDEV, n = 11). GDEV treatment significantly reduced synovitis, proteoglycan loss, and osteoclast activity. Solid and dotted squares in the images indicate the regions magnified for safranin-O and TRAP staining, respectively. (E) Serum biochemistry (AST, ALT, BUN, CRE, AMY) (Cont: n = 6, GDEV: n = 6) and body weight (Cont: n = 10, GDEV: n = 11) showed no systemic toxicity after GDEV treatment. pt, point; field, 500 µm × 500 µm; ns, not significant. $^{**}p < 0.01$, $^{*}p < 0.05$.

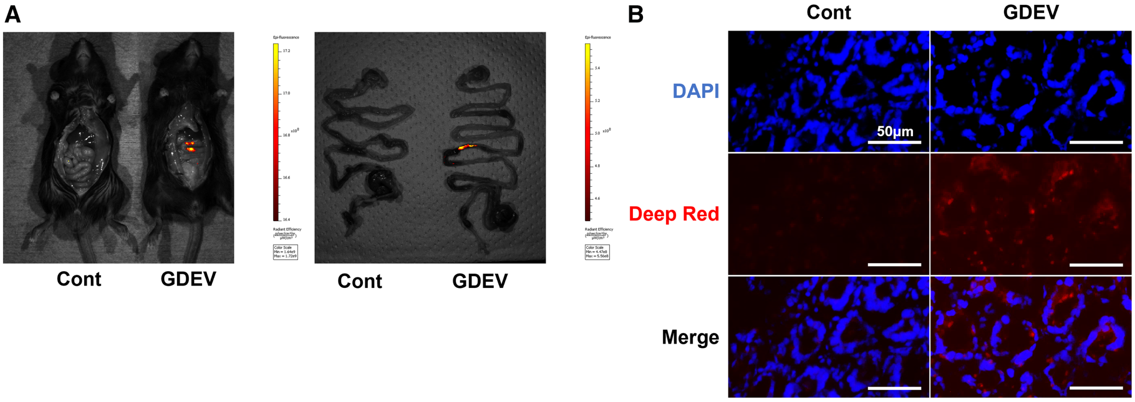

Figure 4. Gastrointestinal absorption of GDEVs

(A) Fluorescence imaging using IVIS demonstrating uptake of orally administered GDEVs labeled with Aco-600 in mice. Mice administered free Aco-600 dye in PBS served as dye-only controls. (B) Representative frozen sections of the small intestine showing the distribution of Aco-600-labeled GDEVs. Nuclei were counterstained with DAPI (blue). Images are shown as DAPI (blue), Aco-600-labeled GDEVs (red), and merged images. Aco-600, fluorescent dye used to label GDEVs.

weight changes were also comparable between the two groups on days 0 and 10 (Control: 22.1 ± 1.3 g to 18.4 ± 2.4 g; GDEV: 22.2 ± 0.8 g to 16.6 ± 1.2 g), with no statistically significant differences (Figure 3E). These findings indicate that oral GDEV administration did not induce systemic toxicity.

Oral bioavailability of GDEVs

Fluorescence imaging using the In Vivo Imaging System (IVIS) Spectrum computed tomography (CT) system showed that fluorescently labeled GDEVs passed through the stomach without visible degradation and reached the small intestine within 2 h after oral administration. In contrast, mice administered the free Aco-600 dye (dye-only controls) showed no detectable intestinal fluorescence, confirming that the observed signal originated from the labeled GDEVs rather than the residual dye (Figure 4A). Subsequent fluorescence microscopy of frozen intestinal sections (prepared with Kawamoto's film method and stained with DAPI) revealed uptake of EVs by intestinal villi (Figure 4B). Furthermore, immunofluorescence staining using an anti-junctional adhesion molecule-A (JAM-A) antibody demonstrated the localization of GDEVs within intestinal epithelial cells, confirming their internalization (Figure S3).

Stability of GDEVs under simulated gastric conditions

To determine whether GDEVs are stable under stomach-like acidic conditions, we performed *in vitro* assays comparing untreated and hydrochloric acid (HCl)-treated EVs. Specifically, GDEVs were exposed to HCl (pH 2.0, 60 min at 37°C), and their biological activity was compared with that of untreated GDEVs, as well as with mesenchymal stem cell-derived EVs (MSCEVs) and HCl-treated MSCEVs. Functional assays demonstrated that the proliferative effect of MSCEV-HCl was reduced by 19.5% compared with untreated MSCEVs, whereas GDEV-HCl retained proliferative activity equivalent to untreated GDEVs (Figure 5A). Similarly, the suppression of inflammatory gene expression (TNFα, IL-1β, IL-6, Cox2, and MMP3) was attenuated in MSCEV-HCl by 36.9%, 23.8%, 38.0%, 35.4%, and 31.4%, respectively, compared with untreated MSCEVs. In contrast, GDEV-HCl showed comparable suppressive effects to untreated GDEVs, indicating no loss of function after acid exposure (Figure 5B). These results suggest that GDEVs are resistant to gastric acid and maintain their biological activity, supporting their feasibility as orally administered therapeutic agents.

Concentration of 6-gingerol and 6-shogaol in GDEVs

Liquid chromatography-mass spectrometry (LC-MS) analysis using a triple quadrupole mass spectrometer (TQD, Waters Corporation, USA) (negative electrospray ionization mode) detected distinct peaks corresponding to the ginger bioactive compounds. 6-Gingerol was detected at a retention time of 3.25–3.75 min, while 6-shogaol was detected at 4.15–4.65 min (Figure 6A). Quantification indicated that 1 mg/mL of GDEV protein contained 6-gingerol at 5,312.3 ± 87.5 ng/mL (range: 5,225–5,400) and 6-shogaol at 137.3 ± 12.5 ng/mL (range: 125–150) ($n = 3$). These findings demonstrate that 6-gingerol is the predominant bioactive compound in GDEVs.

Comparison of anti-inflammatory effects of GDEVs and 6-gingerol

Cell proliferation was assessed using the Cell Counting Kit-8 (CCK-8; Dojindo Laboratories, Kumamoto, Japan) in TNF-α/IL-1β-stimulated human RASFs. Treatment with GDEVs (1X) significantly reduced cell proliferation compared to the inflammation-only group (17.0% reduction) and the 6-gingerol group (10.3% reduction) (Figure 6B).

Similarly, RT-qPCR was performed to evaluate inflammatory and catabolic gene expression in the same cells. GDEV treatment markedly suppressed MMP3 (57.3%), IL-6 (70.0%), and COX-2 (34.4%) expression relative to the inflammation-only group. Notably,

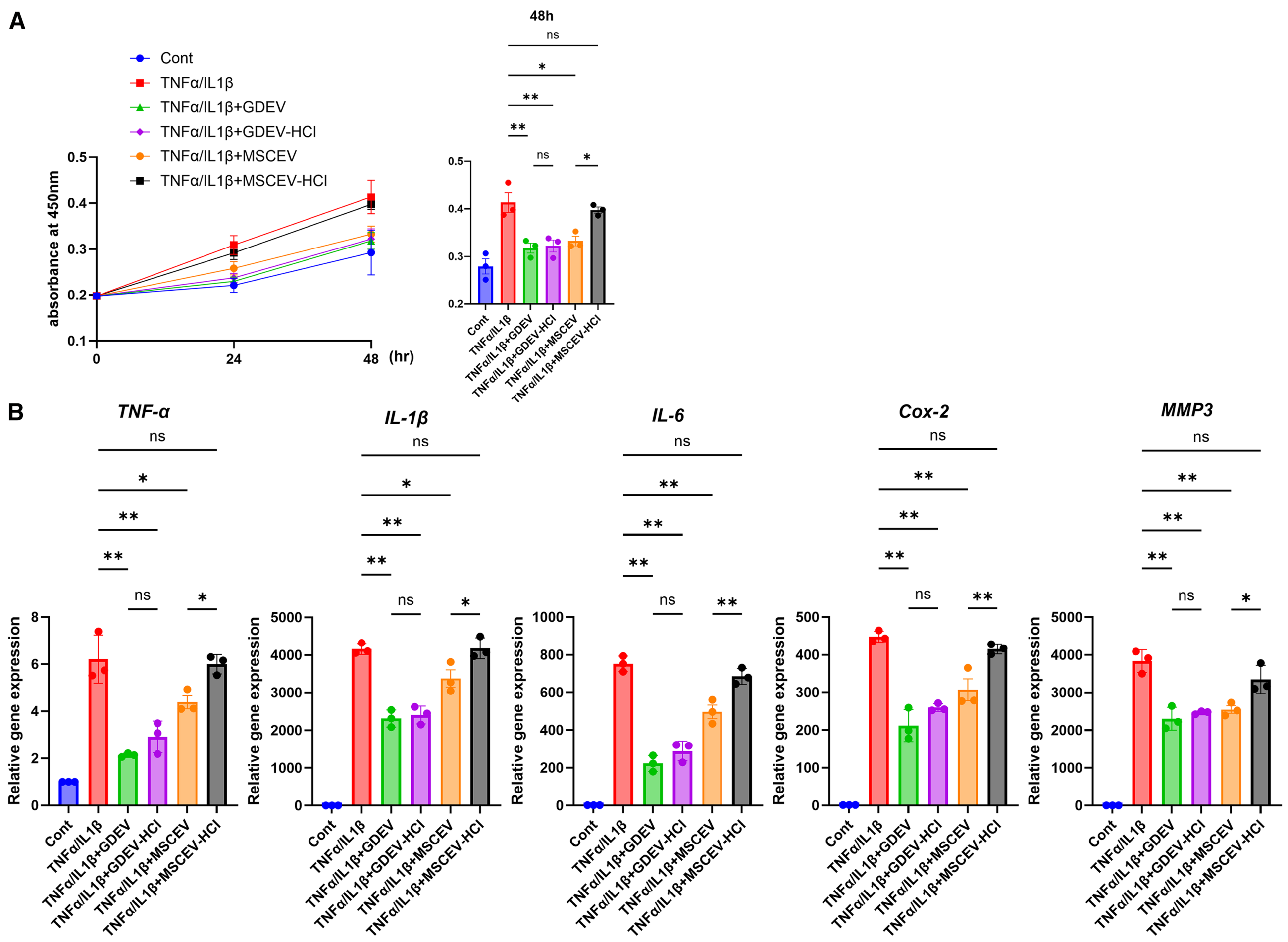

Figure 5. *In vitro* stability of GDEVs under simulated gastric conditions

(A) Cell viability of RASF measured by MTT assay after 48 h. Six groups were analyzed ($n = 3$ per group): Cont, TNFα/IL1β, TNFα/IL1β + GDEV, TNFα/IL1β + GDEV-HCl, TNFα/IL1β + MSCEV, and TNFα/IL1β + MSCEV-HCl. GDEV-HCl retained proliferative activity equivalent to untreated GDEVs, whereas MSCEV-HCl showed reduced proliferation. (B) Gene expression analysis of inflammatory mediators (TNFα, IL-1β, IL-6, COX-2, and MMP3) by RT-qPCR under the same experimental conditions ($n = 3$ per group). GDEV-HCl maintained suppression of inflammatory gene expression comparable to untreated GDEVs, while MSCEV-HCl exhibited attenuated inhibitory effects. ns, not significant. $^{**}p < 0.01$, $^{*}p < 0.05$.

compared with the 6-gingerol group, GDEVs further decreased MMP3, IL-6, and COX-2 expression by 33.2%, 37.8%, and 19.6%, respectively (Figure 6C), indicating that GDEVs reduce inflammatory gene expression more effectively than 6-gingerol alone.

RNA sequencing and miRNA analysis

RNA sequencing (RNA-seq) analysis revealed substantial transcriptional changes in RASFs after inflammation and following GDEV treatment. In Figure 7A, 1,089 genes were upregulated in the inflammation group compared with the control group, and 764 genes were downregulated in the GDEV group compared with the inflammation group. Among these, 192 genes were common to both comparisons. Gene Ontology (GO) enrichment analysis of these 192 genes identified that pathways such as chemotaxis, glucocorticoid receptor pathway, cAMP metabolic process, regulation of acute inflammatory response, and TNFs bind their physiological receptors, all of which are associated with arthritis exacerbation. Notably, these inflammation-related gene sets were suppressed by GDEV treatment.

In Figure 7B, 1,334 genes were downregulated in the inflammation group compared with the control group and 474 genes were upregulated in the GDEV group compared with the inflammation group. A total of 136 genes were common to both comparisons. GO analysis of these 136 genes did not reveal enrichment of arthritis-related pathways, indicating that GDEV treatment did not promote the expression of genes known to exacerbate arthritis.

To investigate whether miRNAs are associated with the anti-inflammatory effects of GDEVs, we performed microarray analysis to identify common miRNAs between MSCEVs, which have been reported to exert anti-inflammatory effects in RA[33] and GDEVs. In Figure 7C, a total of 2,578 miRNAs were detected in MSCEVs, among which

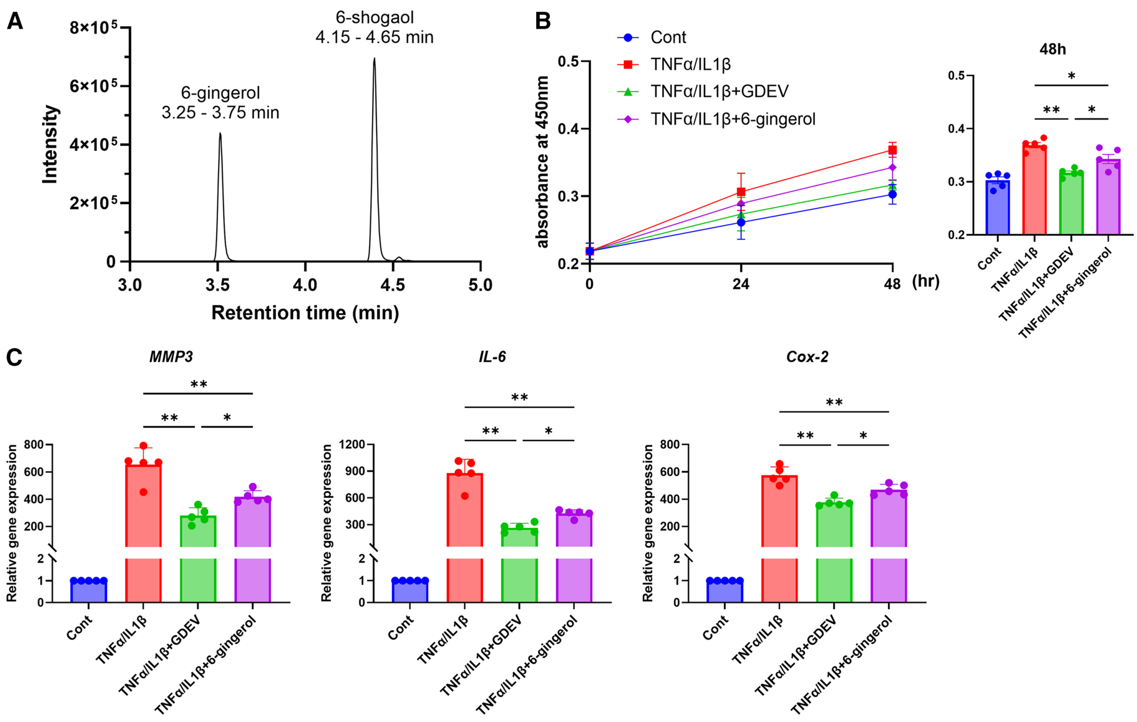

Figure 6. Evaluation of 6-gingerol and GDEVs on RASF

(A) LC-MS chromatogram showing peaks of 6-gingerol (3.25–3.75 min) and 6-shogaol (4.15–4.65 min). (B) Cell viability of RASF measured by MTT assay (n = 5 per group). Four groups were analyzed: Cont, TNFα/IL1β, TNFα/IL1β + GDEV, and TNFα/IL1β + 6-gingerol. GDEVs significantly enhanced RASF viability compared with 6-gingerol. (C) Gene expression analysis of inflammatory mediators (MMP3, IL-6, and COX-2) in RASF following treatment (n = 5 per group). Four groups: Cont, TNFα/IL1β, TNFα/IL1β + GDEV, and TNFα/IL1β + 6-gingerol. GDEVs more effectively suppressed inflammatory gene expression compared with 6-gingerol. **$p < 0.01$, *$p < 0.05$.

2,307 were also present in GDEVs (the full list is available in the supplemental information file). Given the large number of shared miRNAs, we focused on the most abundant species by selecting those ranked within the top 20 in expression level and having expression values greater than 1,000 (Table S3). From this high-expression group, miR-149 was chosen because it has been previously reported in RASFs to inhibit cell proliferation and inflammatory cytokine expression,[34] and it ranked among the top expressed miRNAs in GDEVs, making it a more generalizable candidate compared with the less-characterized miR-6087 and miR-6088.

Cross-comparison of predicted miR-149 target genes (n = 1,129, predicted using TargetScan) with RNA-seq of differentially expressed genes (DEGs) that were downregulated in the GDEV group compared to the inflammation group (n = 764) identified 58 overlapping genes (Figure 7D). GO enrichment analysis of these 58 genes revealed that key arthritis-related pathways were modulated, particularly Rat sarcoma virus oncogene (Ras) signaling and the mitogen-activated protein kinase (MAPK) cascade. Notably, genes such as Fms-related receptor tyrosine kinase 4 (FLT4), phospholipase A2 group IIA (PLA2G2A), kinase suppressor of Ras 1 (KSR1), and tau tubulin kinase 1 (TTBK1) were associated with Ras signaling, while insulin-like growth factor binding protein 4 (IGFBP4), SRY-box transcription factor 9 (SOX9), and dual specificity phosphatase 19 (DUSP19) were linked to the MAPK cascade (Table S4).

To experimentally validate these transcriptomic predictions, we next examined whether miR-149 could recapitulate the anti-inflammatory effects of GDEVs *in vitro*. Under TNF-α/IL-1β stimulation, cell proliferation assessed by CCK-8 was reduced by 13.4% with miR-149 compared to the inflammation-only group (Figure S4A). RT-qPCR further demonstrated that miR-149 significantly suppressed the expression of TNFα (46.0%), IL-1β (28.3%), IL-6 (54.8%), COX-2 (53.0%), and MMP3 (45.8%) relative to the inflammation-only group (Figure S4B).

To further clarify whether miRNAs within GDEVs contribute to their anti-inflammatory effects, we compared the GDEV group, the GDEV treated with ribonuclease (GDEV-RNase) group, and a combination of miR-149 with 6-gingerol. The miR-149 + 6-gingerol group reproduced anti-inflammatory responses comparable to those of the GDEV group, showing 22% suppression of proliferation (Figure 8A) and reductions of TNFα (52.8%), IL-1β (42.0%), IL-6 (56.8%), COX-2 (71.8%), and MMP3 (43.4%) relative to the inflammation group (Figure 8B). In contrast, the GDEV-RNase group showed significantly weaker suppression of proliferation and inflammatory gene expression (proliferation: 13.2% vs. 22.0%; TNFα: 11.3% vs.

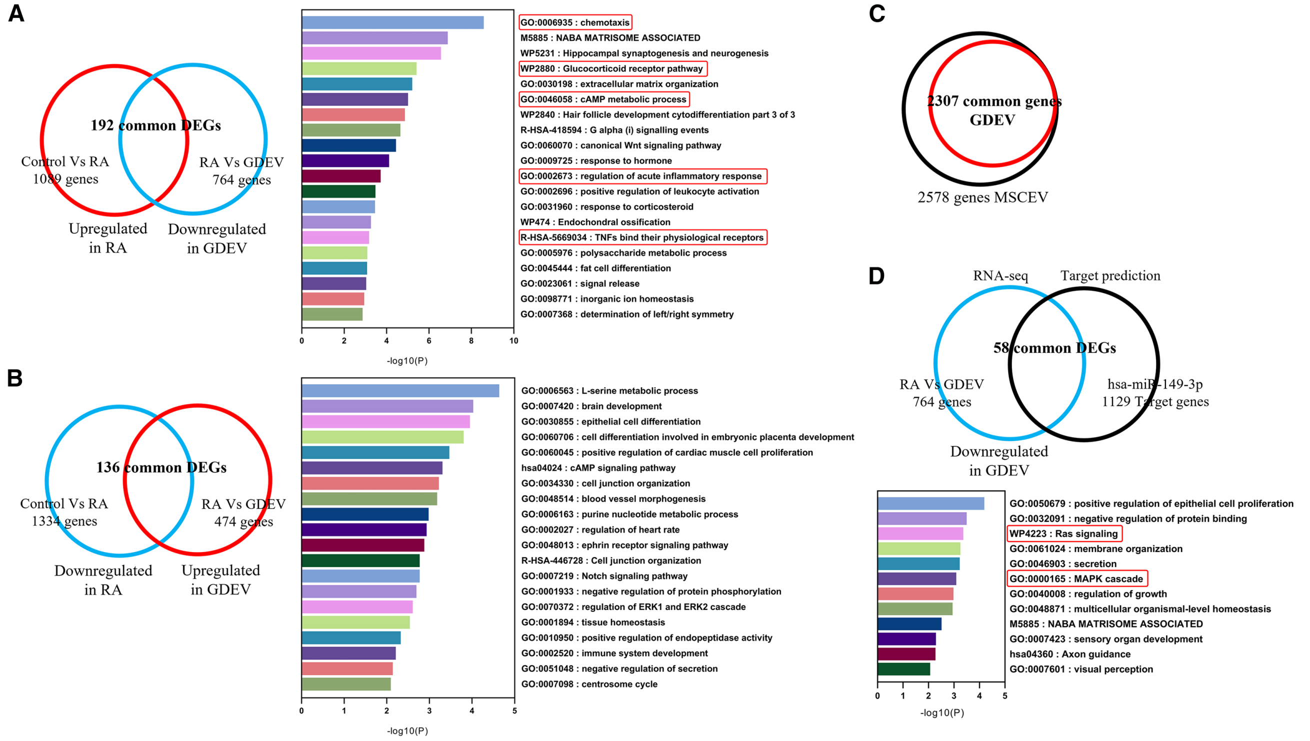

Figure 7. Transcriptomic analysis of GDEV effects on RASFs

(A) DEGs upregulated in RASF under inflammatory conditions (RA group) and downregulated following GDEV treatment. GO enrichment clusters are shown. Genes related to arthritis exacerbation are boxed in red. A total of 192 genes were common between the comparisons (RA vs. control; GDEV vs. RA). (B) DEGs downregulated in RASF under inflammatory conditions (RA group) and upregulated following GDEV treatment. GO enrichment clusters are shown. A total of 136 genes were common between the comparisons. No arthritis-exacerbating pathways were enriched, indicating that GDEV treatment did not promote harmful gene expression. (C) Microarray comparison of human MSCEVs and GDEVs. A total of 2,578 miRNAs were detected in MSCEVs, of which 2,307 were also present in GDEVs. The top-expressed miRNAs (expression value > 1,000) were further analyzed, and miR-149 was selected as a candidate associated with anti-inflammatory effects. (D) RNA-seq analysis and miR-149 target gene identification. Downregulated DEGs in the GDEV-treated group ($n = 764$) were cross-compared with predicted miR-149 targets ($n = 1,129$) to identify 58 overlapping genes. GO enrichment analysis revealed modulation of key arthritis-related pathways, including Ras signaling and the MAPK cascade. Genes related to inflammation are boxed in red. Ras, Rat sarcoma virus oncogene.

52.8%; IL-1β: 2.4% vs. 42.0%; IL-6: −3.0% vs. 56.8%; COX-2: 7.5% vs. 71.8%; MMP3: 13.2% vs. 43.4%) (Figure 8B). To examine whether miR-149 and 6-gingerol exert additive or synergistic effects, we compared the combined treatment with each single treatment. The miR-149 + 6-gingerol group showed an additional 14.9% inhibition of cell proliferation compared with miR-149 alone and 15.4% compared with 6-gingerol alone (Figure S5A). Similarly, the combined treatment produced greater anti-inflammatory effects, suppressing TNFα by an additional 31.0% (vs. miR-149) and 34.9% (vs. 6-gingerol), IL-1β by 22.3% and 22.5%, IL-6 by 28.1% and 32.2%, COX-2 by 61.8% and 58.3%, and MMP3 by 29.6% and 22.5%, respectively (Figure S5B). These findings suggest that the anti-inflammatory effects of GDEVs are at least partly attributable to their miRNA cargo, particularly miR-149, which may act synergistically with 6-gingerol.

DISCUSSION

This study revealed that oral administration of GDEV ameliorated arthritis in mice through its anti-inflammatory effects. GDEV contain high levels of miR-149 and 6-gingerol, both of which inhibited inflammation, cell proliferation, and migration in RASF, suggesting their therapeutic potential in RA. Our results highlight the potential for cross-kingdom communication, in which PDEVs, such as those from ginger, can influence mammalian cells.[35] Unlike MSCEVs, GDEVs offer advantages, such as lower production costs, greater stability, and the added benefit of oral administration, making them viable for large-scale use.[36]

Previous studies have demonstrated that specific bioactive components of ginger, such as 6-gingerol and 6-shogaol, exert anti-inflammatory effects by modulating key pathways including NF-κB and MAPK (Table S5).[17,18] In our study, GDEVs were found to contain higher levels of 6-gingerol compared to 6-shogaol, and importantly, GDEVs exhibited stronger anti-inflammatory effects than either compound alone. These findings indicate that the therapeutic efficacy of GDEVs cannot be attributed solely to the presence of ginger-derived small molecules. Our data further suggest that

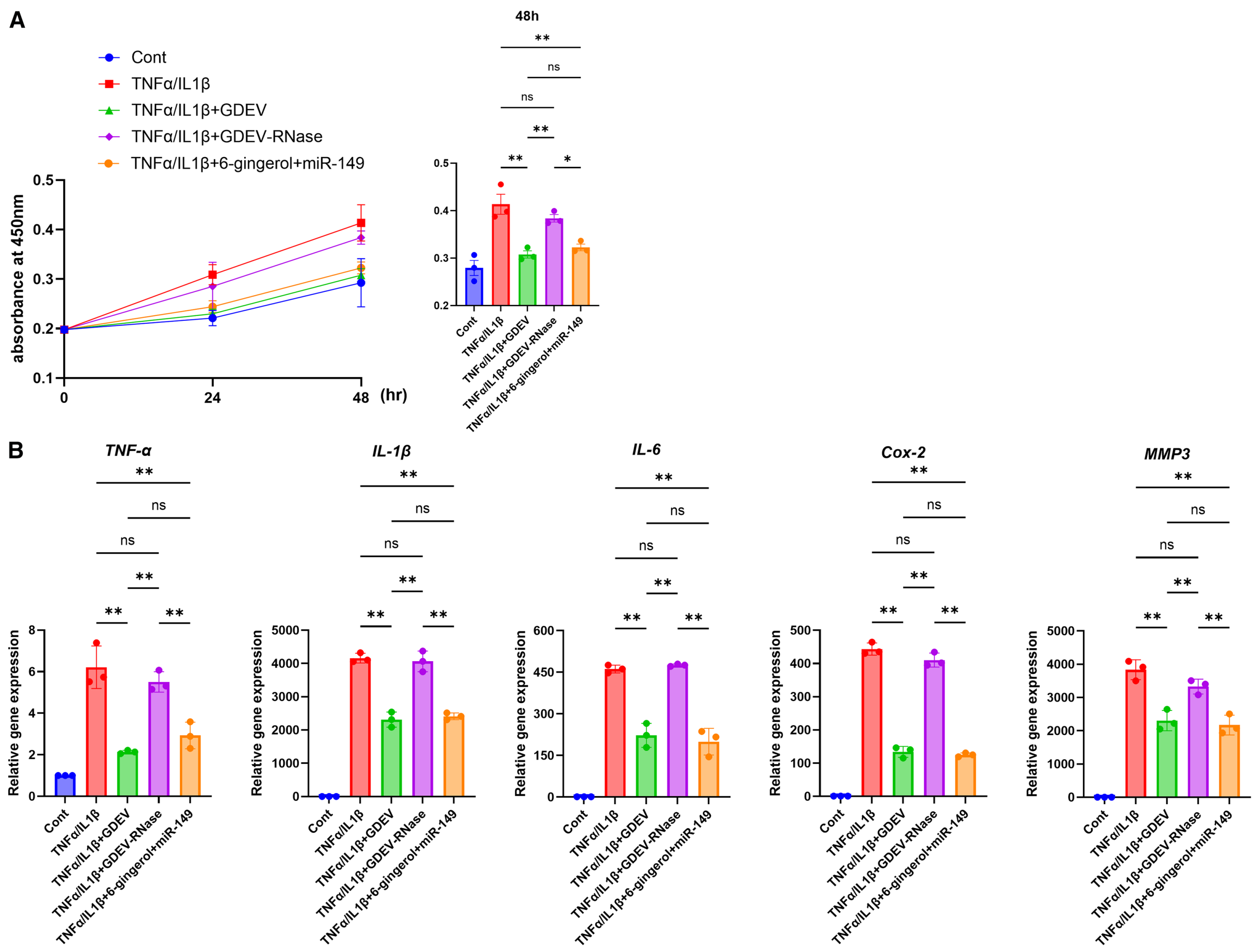

Figure 8. *In vitro* effects of RNA depletion of GDEVs and combination treatment with miR-149 and 6-gingerol on RASF

(A) Cell viability of RASF measured by MTT assay after 48 h ($n = 3$ per group). Five groups were analyzed: Cont, TNFα/IL1β, TNFα/IL1β + GDEV, TNFα/IL1β + GDEV-RNase, and TNFα/IL1β + 6-gingerol + miR-149. The GDEV-RNase group showed significantly weaker suppression of proliferation compared with the GDEV group, whereas the combination of miR-149 and 6-gingerol reproduced anti-inflammatory effects comparable to intact GDEVs. (B) Gene expression analysis of inflammatory mediators (TNFα, IL-1β, IL-6, COX-2, and MMP3) by RT-qPCR under the same experimental conditions ($n = 3$ per group). The GDEV-RNase group exhibited attenuated suppression of inflammatory genes, while the combination of miR-149 and 6-gingerol reproduced effects similar to intact GDEVs. $^{**}p < 0.01$, $^{*}p < 0.05$.

GDEVs provide enhanced therapeutic effects due to the synergistic action of bioactive molecules including miRNAs, proteins, and lipids, combined with efficient cellular uptake of EVs. Among these, miR-149 emerged as a key regulatory component. In line with this, our additional experiments demonstrated that RNase-treated GDEVs lost a significant portion of their anti-inflammatory capacity, supporting the conclusion that GDEV-associated RNase-sensitive cargos, particularly miRNAs, are functionally critical.

Compared to conventional anti-inflammatory therapies such as DMARDs, NSAIDs, and biologics, GDEVs offer several advantages. Conventional RA treatments are associated with high costs and adverse effects, including immunosuppression and increased risk of infection.[8] In contrast, PDEVs provide a natural and potentially safer alternative with fewer side effects, particularly those derived from edible plants, which have been reported to exhibit minimal immunogenicity and favorable biocompatibility.[15] Reported adverse effects of PDEVs are minimal, and their negative zeta potential contributes to colloidal stability, prevents aggregation, and enhances bioavailability.[36] An additional critical advantage is their gastrointestinal stability. While previous studies mainly relied on fluorescent dyes (such as Aco-600) or morphological observation with transmission electron microscopy to suggest intestinal delivery, these approaches could not exclude dye release or failed to assess functional stability.[37] In our study, mice administered free Aco-600 dye in PBS (dye-only controls) showed no detectable intestinal fluorescence, whereas Aco-600-labeled GDEVs were localized within JAM-A^{+} intestinal epithelial cells, confirming their cellular uptake and

supporting their intestinal absorption. To extend these findings, we examined the stability of GDEVs under acidic conditions (HCl, pH 2.0) that mimic gastric acid. Notably, GDEVs maintained significant anti-inflammatory activity in CCK-8 and RT-qPCR assays after acid treatment, whereas MSCEVs lost activity under the same conditions. These results indicate that GDEVs are intrinsically resistant to acidic conditions, a property likely attributable to their lipid bilayer composition.[38] This stability supports their feasibility as orally administered therapeutics. Thus, GDEVs, unlike many mammalian EVs, can remain intact and biologically active through the gastrointestinal tract.

In the present study, RNase treatment of GDEVs markedly reduced their anti-inflammatory effects, indicating that RNA cargos, including miRNAs, play a critical functional role. Analysis of these RNA cargos highlighted miR-149 as a key regulatory molecule mediating the anti-inflammatory effects of GDEVs. RNA-seq revealed that several genes in the Ras and MAPK pathways, including FLT4, PLA2G2A, KSR1, and DUSP19, were significantly downregulated in the EV-treated group compared to the inflammation group (Table S4). These genes are conserved targets of miR-149,[39,40] suggesting that GDEVs exert their effects at least in part through miR-149-mediated suppression of pro-inflammatory signaling. Mechanistically, reduced FLT4 expression could modulate immune cell trafficking,[41] while suppression of PLA2G2A may attenuate eicosanoid production.[42] Consistent downregulation of KSR1 and DUSP19 further implies that GDEVs fine-tune MAPK activity to prevent excessive inflammation.[43,44] These mechanistic insights align with our functional assays, where miR-149 suppressed proliferation and reduced expression of MMP-3, IL-6, COX-2, TNFα, and IL-1β. Taken together, our findings indicate that miR-149 appears to be a central mediator of the RNA-dependent anti-inflammatory activity of GDEVs, although other miRNAs and bioactive cargos may also contribute to the broader therapeutic effects.[16]

Despite these promising findings, this study had several limitations. First, although the *in vitro* experiments using RASFs clearly demonstrated the anti-inflammatory effects of GDEVs, to evaluate potential clinical applications, it is necessary to first confirm these effects *in vivo*. Considering the concept of cross-kingdom communication,[35] in which PDEVs such as GDEVs can exert effects across species, a mouse model was used. As with previous studies on PDEVs, further preclinical research and clinical trials will be necessary to confirm the reproducibility of these findings in human patients with RA and to evaluate the safety and efficacy of GDEVs in clinical settings.[29] Second, the bioavailability and pharmacokinetics of orally administered GDEVs require further investigation. Although our data confirmed intestinal uptake, the circulatory half-life and joint-specific accumulation of GDEVs remain unclear, as we did not measure blood concentration or percent injected dose. Furthermore, unlike mammalian EVs, GDEVs lack specific surface markers such as CD9 or CD63, limiting precise tracking of their biodistribution.[45] In addition, we were unable to quantify whether miR-149 levels increased in the joint after oral GDEV administration. In the mouse arthritis model used in this study, the volume of synovial fluid and the size of joint tissues were extremely small, making reliable quantitative assessment difficult. Moreover, miR-149 is present in GDEVs at relatively low abundance and may be rapidly consumed within inflamed joint tissues, further complicating detection. Future studies using more sensitive analytical approaches or larger-animal models will be required to clarify the *in vivo* kinetics and joint accumulation of miR-149. However, GDEVs may exert systemic anti-inflammatory effects, potentially mediated in part through modulation of the gut microbiota, as reported in models of colitis.[31] In our study, the observed functional outcomes provide indirect evidence that orally administered GDEVs can reach target tissues and exert anti-inflammatory effects. Future investigations should evaluate blood levels, joint distribution, and impacts on the gut microbiome in arthritis models. Third, the optimal dosing regimen and long-term effects of ginger EVs remain unclear. Although direct comparison with raw ginger was not performed in the present study, previous studies have shown that GDEVs contain higher levels of miRNAs and exert stronger anti-inflammatory effects than equivalent amounts of raw or cooked ginger.[16] In the present study, the administered dose was equivalent to approximately 120 individual ginger units per 60-kg human body weight. One ginger unit weighed approximately 35 g, corresponding to a total of roughly 4.2 kg of fresh ginger. Direct consumption of such a large amount of ginger ($\geq$4 g per day) could cause gastrointestinal discomfort or other adverse effects,[46] whereas GDEVs can be concentrated and administered conveniently.[47] Future investigations should evaluate the long-term effects, dose dependency, and optimal dosing intervals of GDEVs to establish safe and effective therapeutic regimens. Last, although miR-149 is a key anti-inflammatory component of GDEVs, the precise mechanisms remain incompletely understood. Combined administration of miR-149 and 6-gingerol reproduced effects comparable to GDEVs, suggesting these are major contributors. GDEVs also contain other miRNAs, proteins, and lipids, which may collectively enhance their therapeutic potential,[16] highlighting their promise as a novel therapeutic strategy.

In conclusion, this study demonstrated that GDEVs exert significant therapeutic effects in RA, partly through RNA cargos such as miR-149 that mediate anti-inflammatory activity. Their oral availability, strong efficacy, and scalable production highlight the novelty and clinical potential of GDEVs as a natural and noninvasive therapeutic strategy for inflammatory diseases.

MATERIALS AND METHODS

All procedures were performed in accordance with the Guidelines for Animal Experimentation, Hiroshima University and with the approval of the Committee of Research Facilities for Laboratory Animal Sciences, Graduate School of Biomedical Sciences, Hiroshima University (Approval number: A22-144).

Isolation and characterization of GDEVs

Isolation of GDEVs

The GDEVs were isolated using the following procedure as previously described.[48,49] Fresh ginger was chopped and homogenized using a blender, and the homogenate was squeezed using a food

squeezer to obtain the crude extract. The extract was sequentially centrifuged at 300 × *g* for 10 min at 4°C, followed by 2,000 × *g* for 20 min at 4°C to remove cellular debris. The supernatant was then filtered through 0.45- and 0.22-μm filters. The filtered extract was ultracentrifuged at 39,500 rpm (approximately 100,000 × *g*) for 160 min at 4°C using a SW55Ti rotor (Beckman Coulter, Brea, CA, USA) in 5.2-mL open-top polypropylene tubes. After ultracentrifugation, the EV pellets were resuspended in 100 μL of phosphate-buffered saline (PBS) per tube, pooled together, and stored at 4°C. The isolated EVs were used for further analysis within 1 week.

High-resolution SE-ADM system

We evaluated EVs using an SE-ADM imaging system based on field emission scanning electron microscopy with an SU5000 (Hitachi High-Tech Corp., Japan), following a method described previously.[50] This system enables observation of biological specimens in water without metal staining. Exosomes were imaged under the following conditions: magnification of 60,000×, resolution of 1,280 × 1,024 pixels, 40 s scanning time, 7 mm working distance, 7 kV accelerating electron beam, and 10 pA current. The original SE-ADM images were processed using a two-dimensional Gaussian filter (GF) with a kernel size of 7 × 7 pixels and a radius of 1.2 σ. Background subtraction was performed by subtracting the SE-ADM images from the filtered images by using a broad GF (400 × 400 pixels, 160 σ). EVs were identified based on their characteristic spherical morphology and a diameter of 50–150 nm, consistent with previous reports.[50] Particles that were damaged, overlapping, or not clearly distinguishable from the background were excluded from analysis. Quantitative measurements were performed on at least three independent images per sample to ensure reproducibility.

Nanoparticle imaging analysis

The size distribution and concentration of the GDEVs were analyzed using a nanoparticle imaging analyzer (VideoDrop, Meiwafosis, Japan).[51] This system enables real-time visualization and quantification of nanoparticles in a liquid suspension without the need for labeling. A diluted EV suspension (1:100 in PBS) was loaded into the VideoDrop chamber, and measurements were performed according to the manufacturer's protocol. VideoDrop software (v.3.2) was used to determine particle size distribution and concentration.[51] Each sample was analyzed in triplicate.

Protein quantification

The protein content of GDEVs was measured using the Qubit Protein Assay Kit (Invitrogen, Carlsbad, CA, USA) according to the manufacturer's instructions.[52] Briefly, the Qubit working solution was prepared using Qubit Reagent and Qubit Buffer. GDEV samples were mixed with the working solution, and fluorescence was measured using the Qubit 2.0 Fluorometer (Invitrogen, Carlsbad, CA, USA).

In vitro experiment: Cell culture and assays

In vitro experiments were performed to evaluate the effects of GDEVs on inflammation and cellular function.

Patients and RASF culture

Fresh synovial tissues were obtained from 5 RA patients (3 females, 2 males; mean age, 65.6 years; range, 57–75; mean disease duration, 12.2 years; range, 5–27) undergoing total knee arthroplasty, total hip arthroplasty, or total ankle arthroplasty at Hiroshima University (Table S6). All patients met the 2010 American College of Rheumatology/European Alliance of Associations for Rheumatology (ACR/EULAR) classification criteria for RA.[53] The study protocol was approved by the Institutional Review Board of Hiroshima University (Approval No. E-508), and written informed consent was obtained from all participants.

Synovial tissue samples (approximately 0.5–1.0 cm^3 each) were collected from three distinct biopsy sites per patient, selected on the basis of macroscopic signs of active inflammation, such as pronounced synovial hypertrophy, vascular proliferation, and erythema. RA affects multiple joints systemically. Thus, tissues were collected from various joints to reflect this systemic inflammation and minimize site-specific bias. Tissues were rinsed with PBS, minced into ~1 mm^3 fragments, and placed in 10-cm culture dishes containing Dulbecco's modified Eagle medium ([DMEM], high glucose, with L-glutamine, phenol red, and sodium pyruvate; FUJIFILM Wako) supplemented with 10% fetal bovine serum ([FBS], Thermo Fisher Scientific) and 1% penicillin-streptomycin-amphotericin B (AB; FUJIFILM Wako).[54] Cultures were maintained at 37°C with 5% CO_2 in a humidified incubator, allowing fibroblast-like synoviocytes to migrate out from tissue explants. Nonadherent tissue pieces were removed after 5–7 days, and the medium was replaced every 3–4 days.

Although RASFs at passage 2 maintain morphological stability, they may still reflect acute inflammatory status from the donor, leading to sample variability. Fourth to sixth passages of RASFs were used for subsequent experiments to ensure high fibroblast purity, reduce patient variability, and maintain RA-specific inflammatory phenotypes.[55]

Induction of inflammation

To induce an inflammatory response, cells were treated with a combination of TNF-α (5 ng/mL, PeproTech, Rocky Hill, NJ, USA) and IL-1β (5 ng/mL, PeproTech, Rocky Hill, NJ, USA) for 24 h. For cell proliferation assays, the cells were treated with TNF-α and IL-1β for 24 h or 48 h, and the proliferation was assessed at both time points. This inflammatory model was selected based on previous studies, which have shown that TNF-α and IL-1β are key pro-inflammatory cytokines involved in RA pathogenesis.[3,56] This ensured a reproducible inflammatory model regardless of donor variability.

Cell proliferation assay

Cell proliferation was evaluated using the 3-(4,5-dimethylthiazol-2-yl)-2,5-diphenyltetrazolium bromide (MTT) Assay (CCK-8; Dojindo Laboratories, Kumamoto, Japan).[49] RASFs were seeded in 96-well plates at a density of 3 × 10^3 cells/well and cultured for 15 h in DMEM (high glucose, without L-glutamine and phenol

red; FUJIFILM WAKO) supplemented with 10% FBS and 1% AB. After this, the following treatment groups were formed: Control group (100 μL/well), DMEM 100 μL; inflammatory group (100 μL/well), DMEM 100 μL + TNFα (5 ng/mL) + IL-1β (5 ng/mL); EV group (100 μL per well), DMEM 95 μL + TNFα (5 ng/mL) + IL-1β (5 ng/mL) + GDEVs (0.5 μL at various concentrations) + PBS (4.5 μL). The GDEVs were diluted in PBS to achieve the final concentration, with the total volume of EVs and PBS adjusted to 5 μL (GDEVs 0.5 μL + PBS 4.5 μL). After treatment, the CCK-8 reagent was added to each well, and the cells were incubated for 1 h. Absorbance was measured at 450 nm using a microplate reader (Tecan Infinite 200 PRO, Tecan Group Ltd., Männedorf, Switzerland) at 0, 24, and 48 h to evaluate cell proliferation.

Gene expression analysis by RT-qPCR

RT-qPCR was performed to evaluate the expression levels of catabolic factors associated with arthritis, as described in our previous reports.[57] Total RNA was extracted from RASFs using ISOGEN reagent (Nippon Gene, Tokyo, Japan) and RNA purification kit (Direct-zol RNA Microprep, Zymo Research). Complementary DNA (cDNA) was synthesized using the iScript Supermix Reverse Transcription System (Bio-Rad Laboratories, Hercules, CA, USA) according to the manufacturer's protocol. Real-time PCR was performed using TaqMan Gene Expression Assay probes (Thermo Fisher Scientific, Waltham, MA, USA) for the following inflammation-related cytokines and enzymes involved in cartilage degradation: TNF-α, IL-1β, IL-6, Cox-2, and MMP3. The assay ID for each gene is listed in the supplemental information (Table S7). The RASFs were seeded in 24-well plates at a density of 5×10^4 cells/well and cultured for 15 h in DMEM supplemented with 10% FBS and 1% AB. After this, the following treatments were applied for 24 h: Control group: DMEM (500 μL); inflammatory group: DMEM (500 μL) + TNFα (5 ng/mL) + IL-1β (5 ng/mL); EV group: DMEM (480 μL) + TNFα (5 ng/mL) + IL-1β (5 ng/mL) + GDEVs (2 μL) + PBS (18 μL). After 24 h of treatment, total RNA was extracted using ISOGEN reagent, and 120 ng of RNA was used for reverse transcription to synthesize cDNA for subsequent RT-qPCR analysis. Gene expression was normalized to that of glyceraldehyde-3-phosphate dehydrogenase (GAPDH), which was used as the internal control. The delta-delta cycle threshold (ΔΔCt) method was used to calculate relative gene expression levels.

Scratch assay for cell migration

The effect of GDEVs on cell migration was evaluated using the scratch assay.[57] RASFs were seeded in 24-well plates at a density of 5×10^4 cells/well and allowed to reach confluence. Once confluence was reached, a straight-line scratch was made through the cell monolayer using a 200 μL pipette tip. After scratching, the cells were washed twice with PBS and replaced with DMEM containing 10% FBS and 1% AB. The following treatment groups were used: Control group (DMEM) and GDEVs group (DMEM + ginger EVs). Migration was monitored using an inverted microscope (Olympus, Tokyo, Japan), and images of the scratched areas were captured at 0, 12, 18, and 24 h. The migrated area was quantified by measuring the difference in the scratch area at each time point relative to the initial area at 0 h using ImageJ software (v.1.53p; NIH, USA).

In vivo experiment

Animal model and treatment

Male DBA/1J mice (Japan SLC, Tokyo, Japan) aged 7–9 weeks (body weight, mean 22.2 ± 1.1 g, range 19.6–24.0 g; $n = 21$) were used in this study, as this strain and sex are commonly employed in CAIA models according to previous reports.[58] After 1 week of acclimatization, mice were housed in temperature-controlled quarters (23°C ± 1°C) with a 12-h light-dark cycle, in groups of two to five per cage (S cage: 143 mm × 293 mm × H148 mm). All mice had free access to food and water. CAIA was induced using a CAIA induction kit (Chondrex, Redmond, WA, USA) as previously reported.[59] Mice were injected intraperitoneally (IP) with a 5-clone monoclonal antibody cocktail (1.5 mg) on day 0, followed by an IP injection of lipopolysaccharide (50 μg) on day 3 to enhance arthritis induction. The mice were monitored daily for development of arthritis. GDEVs or PBS were administered orally. The treatment groups were as follows: EV group ($n = 11$): GDEV 20 μL + PBS 80 μL = 100 μL/day, Control group ($n = 10$): PBS 100 μL/day. Mice were euthanized on day 10 with isoflurane anesthesia followed by cervical dislocation, based on previous reports using the DBA/1J CAIA model.[58] On sacrifice, blood and bilateral ankle joints were collected for further analysis.[60]

Arthritis score evaluation

Arthritis severity was assessed using a qualitative clinical scoring system based on joint swelling and redness, as described previously.[61,62] Each paw was scored individually using the following criteria: 0, normal; 1, mild but definite redness and swelling of the ankle or wrist or redness and swelling limited to individual digits; 2, moderate redness and swelling of the ankle or wrist; 3, severe redness and swelling of the entire paw, including the digits; and 4, maximally inflamed limb involving multiple joints. The total arthritis score was calculated as the sum of the scores from all four paws, with a maximum possible score of 16 points.

Open-field test

To assess locomotor activity and pain-related behavior, mice were subjected to an open-field test on day 10. The test was conducted in a square arena (100 cm long × 100 cm wide × 60 cm high) under a central light intensity of 500 lx based on a previously described method.[63] Each mouse was placed in a corner of the arena and allowed to explore freely for 10 min. Locomotor activity was automatically recorded using a tracking system (SMART; Panlab SL, Barcelona, Spain). The total distance traveled and resting time were measured as key parameters to evaluate movement and pain-related behavior.

Histological analysis

At the time of euthanasia, the ankle joints were collected and fixed in 4% paraformaldehyde (PFA) at 4°C for 48 h. The samples were then decalcified using an ethylenediaminetetraacetic acid (EDTA)-based neutral decalcifying solution (EDT-X; FALMA Co., Tokyo, Japan)

at room temperature for 4 days. After decalcification, the tissues were embedded in paraffin and sectioned into 6-μm-thick slices. Histological evaluation was performed using HE, safranin-O, and fast green staining, as described in our previous reports.[49,57] To evaluate osteoclast activity, TRAP staining was performed using a commercially available kit (Wako Pure Chemical Industries, Ltd., Osaka, Japan) according to the manufacturer's protocol.[64] Several parameters were used in the histological scoring of the foot joints[65,66]: Synovitis score: For synovial inflammation, high-power magnification fields (HPFs) were scored for the percentage of infiltrating mononuclear inflammatory cells as follows: 0 = absent, 1 = mild (1%–10%), 2 = moderate (11%–50%), and 3 = severe (51%–100%). Cartilage degradation score: Cartilage degradation was assessed based on safranin-O staining of proteoglycans, with results expressed as the percentage of cartilage that lost staining: 0 = no loss of staining, 1 = mild loss (1%–10%), 2 = moderate loss (11%–50%), 3 = severe loss (51%–100%). TRAP+ osteoclast score: TRAP-positive multinucleated cells containing more than three nuclei were identified as osteoclasts. Osteoclast counts were determined by evaluating three fields (500 μm × 500 μm each) and calculating their average. Observations and measurements were performed using a BZ-X710 all-in-one microscope (KEYENCE Corporation, Osaka, Japan) and its software. Histological scores were calculated as the sum of both left and right joints. Three different researchers, blinded to the experimental groups, performed the scoring.

Serum parameter evaluation and body weight monitoring

Blood samples were collected via cardiac puncture after euthanasia. After coagulation at 4°C for 1 h, the blood samples were centrifuged at 1,500 × *g* for 30 min at 4°C. The supernatants were collected for subsequent analyses.[60] Serum biomarkers, including AST, ALT, BUN, CRE, and AMY, were analyzed at Nagahama Life Science Laboratory (Nagahama, Japan) using routine laboratory methods. Specifically, AST, ALT, and AMY were measured using the standardized method of the Japan Society of Clinical Chemistry, whereas BUN and CRE were measured using enzymatic methods. These analyses were performed to assess the potential systemic side effects of the treatment. Additionally, changes in body weight were monitored on days 0, 3, 5, 7, 9, and 10 to evaluate any systemic or side effects of the treatment.

Evaluation of oral absorption of GDEVs

Fluorescence imaging analysis

Fluorescence imaging was performed using an IVIS Spectrum CT instrument (PerkinElmer Inc., Waltham, MA, USA).[67] The EVs were labeled with Aco-600 (Acoerela, Inc., Singapore) at a final concentration of 5 μM before administration.[68] Living Image 4.3.1 software was used to analyze the images. The excitation filter was set at 640 nm to capture the fluorescence signals. C57BL/6J male mice (8–10 weeks old; Japan SLC, Tokyo, Japan) were orally administered either labeled GDEVs (20 μL GDEVs +80 μL PBS = 100 μL) or free Aco-600 dye in PBS (5 μM, 100 μL; dye-only control) after an 18-h fasting period, under the same dosing regimen described in the *in vivo* experiment section. Fluorescence imaging was performed 2 h post-administration using the IVIS system.

Tissue distribution analysis (fluorescence microscopy on frozen sections)

At the designated time points, the animals were euthanized, and the small intestine, where fluorescence was detected via IVIS, was harvested. The tissues were immediately frozen in Optimal Cutting Temperature (OCT) compound (Tissue-Tek, Sakura Finetek, Tokyo, Japan) and sectioned into 5-μm thick slices using a CryoStar NX50 (Thermo Fisher Scientific, Waltham, MA, USA) with Kawamoto's film method (SECTION-LAB, Co. Ltd., Yokohama, Japan). Briefly, Kawamoto's film method employs a special adhesive film to support the frozen tissue during sectioning, which allows preparation of high-quality thin sections without curling or fragmentation, even from fragile or undecalcified tissues.[69] The frozen sections were stained with DAPI (Dojindo Laboratories Co., Ltd. Kumamoto, Japan) to visualize nuclei and were then analyzed using a BZ-X710 all-in-one fluorescence microscope (KEYENCE, Osaka, Japan).[57]

In vitro *stability of GDEVs under simulated gastric conditions*

To evaluate the stability of GDEVs in stomach-like acidic conditions, GDEVs were incubated with HCl (pH 2.0; Fujifilm Wako Pure Chemical, Tokyo, Japan) at 37°C for 1 h. After incubation, the samples were re-purified by ultracentrifugation at 39,500 rpm for 160 min at 4°C to remove residual acid.[37,70] The same procedure was applied to MSCEVs to obtain HCl-treated MSCEVs. The recovered GDEV-HCl and MSCEV-HCl were subsequently applied to RASF for functional assays. Cell proliferation was assessed using the method described above, and the expression levels of inflammatory genes (TNFα, IL1β, IL6, Cox2, and MMP3) were determined by RT-qPCR. Untreated GDEVs and untreated MSCEVs were used as controls.

6-Gingerol concentration measurement

The beneficial effects of ginger can be attributed to biologically active compounds in its rhizome, such as gingerols and shogaols, which are known for their anti-inflammatory properties.[18] Among the various bioactive compounds found in ginger, 6-gingerol is the most abundant and pharmacologically active constituent.[71,72] To quantify the concentration of 6-gingerol in GDEVs, LC-MS analysis was performed by TQD triple quadrupole mass spectrometer (Waters, USA) using negative-mode electrospray ionization (ESI−).[37] A reference standard of 6-gingerol (purchased from Selleckchem, USA) was used for calibration, and curcumin was employed as internal standard for analysis.[73] The parameters of the mass spectrometer were optimized as follows: capillary voltage, −3,000 V; core voltage, −20 V for 6-gingerol, −22 V for curcumin; source temperature, 120°C; desolvation temperature, 350°C; desolvation gas flow, 600 L/h; cone gas flow, 50 L/h; collision, 10 for gingerol and 18 for curcumin detection; the m/z 293 > 193 and m/z 367 > 149 ion transitions were employed for gingerol and curcumin detection as (M-H)−. Chromatographic separation was using ACQUITY UPLC system and BEH C18 column (130 Å, 1.7 μm, 2.1 mm × 50 mm, Waters, USA).[73] Chromatographic separations were carried out at 40°C. The mobile phases consisted of 10% acetonitrile containing 0.1% formic acid (A) and 10% acetonitrile containing 0.1% formic acid (B).

Gradient elution at a flow of 200 μL/min was performed with changing %A as follows: 0–7 min: 0%–100%; 7.5–8.5 min: 100%; 8.5–9 min 100%–0%: 9–10 min: 0%. The extracted EV samples were analyzed using an LC-MS system operated under optimized conditions, including multiple reaction monitoring (MRM) mode, to ensure high sensitivity and specificity.

RNA-seq and miRNA analysis

RNA-seq analysis (DEG analysis and pathway analysis)

RNA-seq was done to analyze the gene expression profiles of Control, Inflammation, and GDEV-treated RASFs.[74] Low-quality reads (such as those containing adaptor contamination or with low Phred quality scores) were filtered out to ensure that only high-quality reads were retained, and the remaining high-quality reads were mapped to the human reference genome (GRCh38). Gene expression was normalized using appropriate methods for RNA-seq, and DEGs were identified between the following groups: Control vs. Inflammation and Inflammation vs. GDEV-treated.[75] The RNA-seq data were submitted for analysis to Bioinformatics, Inc. (Tokyo, Japan), where the gene expression data were evaluated. DEGs were then subjected to GO enrichment analysis using the gprofiler2 (v.0.2.1) and clusterProfiler (v.4.6.2) packages to identify biological processes, cellular components, and molecular functions associated with gene expression changes. These R packages are widely used for functional enrichment and pathway analysis, allowing the identification of overrepresented GO terms and pathways from DEG lists and providing robust statistical control for multiple testing.[76,77] These analyses provide insights into the biological mechanisms underlying the therapeutic effects of GDEVs.

Microarray analysis of miRNA

Microarray analysis was performed using a human miRNA chip (Filgen, Inc., Nagoya, Japan) to detect hsa-miRs enriched in GDEVs.[78] The hsa-miRNA profiles were compared with those of MSCEVs to identify common hsa-miRs, as MSCEVs are also known to contain therapeutic miRNAs.[79,80] The analysis focused on hsa-miRs, which are potentially involved in the therapeutic effects of GDEVs. Among the detected miRNAs, those with expression values greater than 1,000 were selected for further investigation, as highly abundant miRNAs in EVs are more likely to exert functional effects due to their competitive binding to target mRNAs.[81] Target genes of highly expressed miRNAs were identified using TargetScan (https://www.targetscan.org), and a cross-study comparison was performed with genes identified by RNA-seq, which had showed decreased expression following GDEV administration. This approach helped identify common miRNA-targeted genes and those downregulated by GDEV treatment. Commonly expressed genes were extracted and subjected to GO enrichment analysis using Metascape (https://metascape.org) to identify the associated biological processes, cellular components, and molecular functions associated with the identified genes.[82]

Transfection of miR-149-3p mimic into RASF

See supplemental information.

RNA depletion of GDEVs and combination treatment with miR-149 and 6-gingerol

To deplete RNA from GDEVs, purified GDEVs were mixed with RNase (10 μg/mL; Nippon Gene, Tokyo, Japan) and incubated at 37°C for 1 h. The GDEV-RNase was subsequently washed with PBS and pelleted by ultracentrifugation (39,500 rpm, 160 min, 4°C).[83] The resulting GDEV-RNase was used for functional assays.

For combination experiments, RASF were transfected with miR-149 mimics as described in the supplemental information and treated with 6-gingerol at the same concentration as described above.

Statistical analysis

Statistical analyses were performed using GraphPad Prism 10.0 (San Diego, CA, USA). Data were analyzed using an unpaired t test for comparisons between two groups and one-way ANOVA with Tukey's post-hoc test for comparisons among three or more groups. Tukey's post-hoc test was applied to correct for multiple comparisons and is widely used to identify specific group differences after ANOVA.[84] Results are presented as mean ± standard deviation. For all statistical analyses, a p value <0.05 was considered statistically significant.

DATA AVAILABILITY

The datasets during and/or analyzed during the current study are available from the corresponding author on reasonable request.

ACKNOWLEDGMENTS

We thank E. Ueda, T. Miyata, and Y. Takagi for their technical support. We would like to thank Editage (www.editage.com) for English language editing. This research was supported by a JCR Grant for Promoting Research for Early RA, the JSPS Program for Forming Japan's Peak Research Universities (JSPS J-PEAKS), and the Japan Agency for Medical Research and Development (AMED) under the HK^2-MIRAI Program (grant number JP256f0137011). The LC-MS analysis was conducted using facilities at the Natural Science Center for Basic Research and Development (N-BARD) and Hiroshima University (NBARD-0100).

AUTHOR CONTRIBUTIONS

H.K., T.N., and S.M. contributed to the study conception and design. Material preparation, experimental procedures, and data analysis were performed by H.K., T.N., Y.D., D.M., R.K., T.O., and S.M. The first draft of the manuscript was written by H.K., and all authors commented on previous versions of the manuscript. All authors read and approved of the final manuscript.

DECLARATION OF INTERESTS

The authors declare no competing interests.

SUPPLEMENTAL INFORMATION

Supplemental information can be found online at https://doi.org/10.1016/j.omtn.2026.102840.

REFERENCES

1. Evangelatos, G., Fragoulis, G.E., Koulouri, V., and Lambrou, G.I. (2019). MicroRNAs in rheumatoid arthritis: From pathogenesis to clinical impact. Autoimmun. Rev. *18*, 102391.

2. Smolen, J.S., Aletaha, D., Barton, A., Burmester, G.R., Emery, P., Firestein, G.S., Kavanaugh, A., McInnes, I.B., Solomon, D.H., Strand, V., et al. (2018). Rheumatoid arthritis. Nat. Rev. Dis. Primers *4*, 18001.

3. Nygaard, G., and Firestein, G.S. (2020). Restoring synovial homeostasis in rheumatoid arthritis by targeting fibroblast-like synoviocytes. Nat. Rev. Rheumatol. *16*, 316–333.
4. Figus, F.A., Piga, M., Azzolin, I., McConnell, R., and Iagnocco, A. (2021). Rheumatoid arthritis: Extra-articular manifestations and comorbidities. Autoimmun. Rev. *20*, 102776.
5. Hansildaar, R., Vedder, D., Baniaamam, M., Tausche, A.K., Gerritsen, M., and Nurmohamed, M.T. (2021). Cardiovascular risk in inflammatory arthritis: rheumatoid arthritis and gout. Lancet. Rheumatol. *3*, e58–e70.
6. Burmester, G.R., and Pope, J.E. (2017). Novel treatment strategies in rheumatoid arthritis. Lancet *389*, 2338–2348.
7. Bluml, S. (2020). [Biologicals and small molecules for rheumatoid arthritis]. Z. Rheumatol. *79*, 223–231.
8. Sparks, J.A. (2019). Rheumatoid Arthritis. Ann. Intern. Med. *170*, ITC1–ITC16.
9. Withrow, J., Murphy, C., Liu, Y., Hunter, M., Fulzele, S., and Hamrick, M.W. (2016). Extracellular vesicles in the pathogenesis of rheumatoid arthritis and osteoarthritis. Arthritis Res. Ther. *18*, 286.
10. Foers, A.D., Cheng, L., Hill, A.F., Wicks, I.P., and Pang, K.C. (2017). Review: Extracellular Vesicles in Joint Inflammation. Arthritis Rheumatol. *69*, 1350–1362.
11. Nawaz, M., Shah, N., Zanetti, B.R., Maugeri, M., Silvestre, R.N., Fatima, F., Neder, L., and Valadi, H. (2018). Extracellular Vesicles and Matrix Remodeling Enzymes: The Emerging Roles in Extracellular Matrix Remodeling, Progression of Diseases and Tissue Repair. Cells *7*, 167.
12. Del Pozo-Acebo, L., López de Las Hazas, M.C., Margollés, A., Dávalos, A., and García-Ruiz, A. (2021). Eating microRNAs: pharmacological opportunities for cross-kingdom regulation and implications in host gene and gut microbiota modulation. Br. J. Pharmacol. *178*, 2218–2245.
13. Del Pozo-Acebo, L., López de Las Hazas, M.C., Tomé-Carneiro, J., Del Saz-Lara, A., Gil-Zamorano, J., Balaguer, L., Chapado, L.A., Busto, R., Visioli, F., and Dávalos, A. (2022). Therapeutic potential of broccoli-derived extracellular vesicles as nanocarriers of exogenous miRNAs. Pharmacol. Res. *185*, 106472.
14. Ito, Y., Taniguchi, K., Kuranaga, Y., Eid, N., Inomata, Y., Lee, S.W., and Uchiyama, K. (2021). Uptake of MicroRNAs from Exosome-Like Nanovesicles of Edible Plant Juice by Rat Enterocytes. Int. J. Mol. Sci. *22*, 3749.
15. Song, L., Fang, Y., Chen, L., Wang, J., and Chen, X. (2021). Role of non-coding RNAs in plant immunity. Plant Commun. *2*, 100180.
16. Yin, L., Yan, L., Yu, Q., Wang, J., Liu, C., Wang, L., and Zheng, L. (2022). Characterization of the MicroRNA Profile of Ginger Exosome-like Nanoparticles and Their Anti-Inflammatory Effects in Intestinal Caco-2 Cells. J. Agric. Food Chem. *70*, 4725–4734.
17. Ballester, P., Cerda, B., Arcusa, R., Marhuenda, J., Yamedjeu, K., and Zafrilla, P. (2022). Effect of Ginger on Inflammatory Diseases. Molecules *27*, 7223.
18. Pazmandi, K., Szollosi, A.G., and Fekete, T. (2024). The "root" causes behind the anti-inflammatory actions of ginger compounds in immune cells. Front. Immunol. *15*, 1400956.
19. Jo, S., Samarpita, S., Lee, J.S., Lee, Y.J., Son, J.E., Jeong, M., Kim, J.H., Hong, S., Yoo, S.A., Kim, W.U., et al. (2022). 8-Shogaol inhibits rheumatoid arthritis through targeting TAK1. Pharmacol. Res. *178*, 106176.
20. Zhang, Y.M., Shen, J., Zhao, J.M., Guan, J., Wei, X.R., Miao, D.Y., Li, W., Xie, Y.C., and Zhao, Y.Q. (2021). Cedrol from Ginger Ameliorates Rheumatoid Arthritis via Reducing Inflammation and Selectively Inhibiting JAK3 Phosphorylation. J. Agric. Food Chem. *69*, 5332–5343.
21. Chando, A., Basudkar, V., Gharat, S., Momin, M., and Khan, T. (2024). Development and preclinical assessment of nanoemulgel loaded with phytoconstituents for the management of rheumatoid arthritis. Drug Deliv. Transl. Res. *14*, 524–541.
22. Li, N., Li, X., Deng, L., Yang, H., Gong, Z., Wang, Q., Pan, D., Zeng, S., and Chen, J. (2023). 6-Shogaol inhibits the proliferation, apoptosis, and migration of rheumatoid arthritis fibroblast-like synoviocytes via the PI3K/AKT/NF-kappaB pathway. Phytomedicine *109*, 154562.
23. Hwang, Y.H., Kim, T., Kim, R., and Ha, H. (2018). The Natural Product 6-Gingerol Inhibits Inflammation-Associated Osteoclast Differentiation via Reduction of Prostaglandin E(2) Levels. Int. J. Mol. Sci. *19*, 2068.
24. Villalvilla, A., da Silva, J.A., Largo, R., Gualillo, O., Vieira, P.C., Herrero-Beaumont, G., and Gómez, R. (2014). 6-Shogaol inhibits chondrocytes' innate immune responses and cathepsin-K activity. Mol. Nutr. Food Res. *58*, 256–266.
25. Levy, A.S., Simon, O., Shelly, J., and Gardener, M. (2006). 6-Shogaol reduced chronic inflammatory response in the knees of rats treated with complete Freund's adjuvant. BMC Pharmacol. *6*, 12.
26. Zick, S.M., Djuric, Z., Ruffin, M.T., Litzinger, A.J., Normolle, D.P., Alrawi, S., Feng, M.R., and Brenner, D.E. (2008). Pharmacokinetics of 6-gingerol, 8-gingerol, 10-gingerol, and 6-shogaol and conjugate metabolites in healthy human subjects. Cancer Epidemiol. Biomarkers Prev. *17*, 1930–1936.
27. Zhu, H., and He, W. (2023). Ginger: a representative material of herb-derived exosome-like nanoparticles. Front. Nutr. *10*, 1223349.
28. Liu, X., Lou, K., Zhang, Y., Li, C., Wei, S., and Feng, S. (2024). Unlocking the Medicinal Potential of Plant-Derived Extracellular Vesicles: current Progress and Future Perspectives. Int. J. Nanomedicine *19*, 4877–4892.
29. Han, R., Zhou, D., Ji, N., Yin, Z., Wang, J., Zhang, Q., Zhang, H., Liu, J., Liu, X., Liu, H., et al. (2025). Folic acid-modified ginger-derived extracellular vesicles for targeted treatment of rheumatoid arthritis by remodeling immune microenvironment via the PI3K-AKT pathway. J. Nanobiotechnology *23*, 41.
30. Zeng, Y., Yu, S., Lu, L., Zhang, J., and Xu, C. (2024). Ginger-derived nanovesicles attenuate osteoarthritis progression by inhibiting oxidative stress via the Nrf2 pathway. Nanomedicine (Lond) *19*, 2357–2373.
31. Yan, L., Cao, Y., Hou, L., Luo, T., Li, M., Gao, S., Wang, L., Sheng, K., and Zheng, L. (2024). Ginger exosome-like nanoparticle-derived miRNA therapeutics: A strategic inhibitor of intestinal inflammation. J. Adv. Res. *69*, 1–15.
32. OECD (2008). Test No. 407: Repeated Dose 28-Day Oral Toxicity Study in Rodents. In OECD Guidelines for the Testing of Chemicals, Section 4 (Paris, France: OECD Publishing), https://www.oecd.org/content/dam/oecd/en/publications/reports/2008/10/test-no-407-repeated-dose-28-day-oral-toxicity-study-in-rodents_g1gh292f/9789264070684-en.pdf.
33. Shin, T.H., Kim, H.S., Kang, T.W., Lee, B.C., Lee, H.Y., Kim, Y.J., Shin, J.H., Seo, Y., Won Choi, S., Lee, S., et al. (2016). Human umbilical cord blood-stem cells direct macrophage polarization and block inflammasome activation to alleviate rheumatoid arthritis. Cell Death Dis. *7*, e2524.
34. Su, W., Ye, Z., Wang, G., Huang, H., and Fang, Y. (2024). Circ_0008410 contributes to fibroblast-like synoviocytes dysfunction by regulating miR-149-5p/HIPK2 axis. Microbiol. Immunol. *68*, 100–110.
35. Alshehri, B. (2021). Plant-derived xenomiRs and cancer: Cross-kingdom gene regulation. Saudi J. Biol. Sci. *28*, 2408–2422.
36. Alzahrani, F.A., Khan, M.I., Kameli, N., Alsahafi, E., and Riza, Y.M. (2023). Plant-Derived Extracellular Vesicles and Their Exciting Potential as the Future of Next-Generation Drug Delivery. Biomolecules *13*, 839.
37. Zhang, M., Viennois, E., Prasad, M., Zhang, Y., Wang, L., Zhang, Z., Han, M.K., Xiao, B., Xu, C., Srinivasan, S., et al. (2016). Edible ginger-derived nanoparticles: A novel therapeutic approach for the prevention and treatment of inflammatory bowel disease and colitis-associated cancer. Biomaterials *101*, 321–340.
38. Woith, E., Guerriero, G., Hausman, J.F., Renaut, J., Leclercq, C.C., Weise, C., Legay, S., Weng, A., and Melzig, M.F. (2021). Plant Extracellular Vesicles and Nanovesicles: Focus on Secondary Metabolites, Proteins and Lipids with Perspectives on Their Potential and Sources. Int. J. Mol. Sci. *22*, 3719.
39. Shi, J., Shan, S., Li, H., Song, G., and Li, Z. (2017). Anti-inflammatory effects of millet bran derived-bound polyphenols in LPS-induced HT-29 cell via ROS/miR-149/Akt/NF-kappaB signaling pathway. Oncotarget *8*, 74582–74594.
40. Zhang, Q., Su, J., Wang, Z., Qi, H., Ge, Z., Li, Z., Chen, W.D., and Wang, Y.D. (2017). MicroRNA-149* suppresses hepatic inflammatory response through antagonizing STAT3 signaling pathway. Oncotarget *8*, 65397–65406.
41. Kuonqui, K., Campbell, A.C., Sarker, A., Roberts, A., Pollack, B.L., Park, H.J., Shin, J., Brown, S., Mehrara, B.J., and Kataru, R.P. (2023). Dysregulation of Lymphatic Endothelial VEGFR3 Signaling in Disease. Cells *13*, 68.
42. Taketomi, Y., and Murakami, M. (2022). Regulatory Roles of Phospholipase A(2) Enzymes and Bioactive Lipids in Mast Cell Biology. Front. Immunol. *13*, 923265.

43. Ingersoll, M.A., Lutze, R.D., Kelmann, R.G., Kresock, D.F., Marsh, J.D., Quevedo, R.V., Zuo, J., and Teitz, T. (2023). KSR1 knockout mouse model demonstrates MAPK pathway's key role in cisplatin- and noise-induced hearing loss. Pre print at. bioRxiv. https://doi.org/10.1101/2023.11.08.566316.

44. Yao, Z.Z., Hu, A.X., and Liu, X.S. (2017). DUSP19 regulates IL-1beta-induced apoptosis and MMPs expression in rat chondrocytes through JAK2/STAT3 signaling pathway. Biomed. Pharmacother. *96*, 1209–1215.

45. Kalluri, R., and LeBleu, V.S. (2020). The biology, function, and biomedical applications of exosomes. Science *367*, eaau6977.

46. Aregawi, L.G., and Zoltan, C. (2025). Evaluation of Adverse Effects and Tolerability of Dietary Ginger Supplementation in Patients With Functional Dyspepsia. Curr. Ther. Res. Clin. Exp. *102*, 100792.

47. Man, F., Meng, C., Liu, Y., Wang, Y., Zhou, Y., Ma, J., and Lu, R. (2021). The Study of Ginger-Derived Extracellular Vesicles as a Natural Nanoscale Drug Carrier and Their Intestinal Absorption in Rats. AAPS PharmSciTech *22*, 206.

48. Welsh, J.A., Goberdhan, D.C.I., O'Driscoll, L., Buzas, E.I., Blenkiron, C., Bussolati, B., Cai, H., Di Vizio, D., Driedonks, T.A.P., Erdbrügger, U., et al. (2024). Minimal information for studies of extracellular vesicles (MISEV2023): From basic to advanced approaches. J. Extracell. Vesicles *13*, e12404.

49. Hayashi, Y., Yimiti, D., Sanada, Y., Ding, C., Omoto, T., Ogura, T., Nakasa, T., Ishikawa, M., Hiemori, K., Tateno, H., et al. (2022). The therapeutic capacity of bone marrow MSC-derived extracellular vesicles in Achilles tendon healing is passage-dependent and indicated by specific glycans. FEBS Lett. *596*, 1047–1058.

50. Ogura, T. (2015). Nanoscale analysis of unstained biological specimens in water without radiation damage using high-resolution frequency transmission electric-field system based on FE-SEM. Biochem. Biophys. Res. Commun. *459*, 521–528.

51. Turkki, V., Alppila, E., Yla-Herttuala, S., and Lesch, H.P. (2021). Experimental Evaluation of an Interferometric Light Microscopy Particle Counter for Titering and Characterization of Virus Preparations. Viruses *13*, 939.

52. Bocian, A., Slawek, S., Jaromin, M., Hus, K.K., Buczkowicz, J., Lysiak, D., Petrilla, V., Petrillova, M., and Legath, J. (2020). Comparison of Methods for Measuring Protein Concentration in Venom Samples. Animals (Basel) *10*, 448.

53. Aletaha, D., Neogi, T., Silman, A.J., Funovits, J., Felson, D.T., Bingham, C.O., 3rd, Birnbaum, N.S., Burmester, G.R., Bykerk, V.P., Cohen, M.D., et al. (2010). 2010 rheumatoid arthritis classification criteria: an American College of Rheumatology/European League Against Rheumatism collaborative initiative. Ann. Rheum. Dis. *69*, 1580–1588.

54. Nishida, K., Komiyama, T., Miyazawa, S.I., Shen, Z.N., Furumatsu, T., Doi, H., Yoshida, A., Yamana, J., Yamamura, M., Ninomiya, Y., et al. (2004). Histone deacetylase inhibitor suppression of autoantibody-mediated arthritis in mice via regulation of p16INK4a and p21(WAF1/Cip1) expression. Arthritis Rheum. *50*, 3365–3376.

55. Hu, F., Shi, L., Mu, R., Zhu, J., Li, Y., Ma, X., Li, C., Jia, R., Yang, D., Li, Y., et al. (2013). Hypoxia-inducible factor-1alpha and interleukin 33 form a regulatory circuit to perpetuate the inflammation in rheumatoid arthritis. PLoS One *8*, e72650.

56. Yu, F.Y., Xie, C.Q., Jiang, C.L., Sun, J.T., and Huang, X.W. (2018). TNF-alpha increases inflammatory factor expression in synovial fibroblasts through the toll-like receptor-3-mediated ERK/AKT signaling pathway in a mouse model of rheumatoid arthritis. Mol. Med. Rep. *17*, 8475–8483.

57. Yimiti, D., Uchibe, K., Toriyama, M., Hayashi, Y., Ikuta, Y., Nakasa, T., Akiyama, H., Watanabe, H., Kondoh, G., Takimoto, A., et al. (2025). CD1530, selective RARgamma agonist, facilitates Achilles tendon healing by modulating the healing environment including less chondrification in a mouse model. J. Orthop. Res. *43*, 273–284.

58. Nagata, Y., Nakasa, T., Mochizuki, Y., Ishikawa, M., Miyaki, S., Shibuya, H., Yamasaki, K., Adachi, N., Asahara, H., and Ochi, M. (2009). Induction of apoptosis in the synovium of mice with autoantibody-mediated arthritis by the intraarticular injection of double-stranded MicroRNA-15a. Arthritis Rheum. *60*, 2677–2683.

59. Hutamekalin, P., Saito, T., Yamaki, K., Mizutani, N., Brand, D.D., Waritani, T., Terato, K., and Yoshino, S. (2009). Collagen antibody-induced arthritis in mice: development of a new arthritogenic 5-clone cocktail of monoclonal anti-type II collagen antibodies. J. Immunol. Methods *343*, 49–55.

60. Ding, C., Yimiti, D., Sanada, Y., Matsubara, Y., Nakasa, T., Matsubara, K., Adachi, N., and Miyaki, S. (2024). High-fat diet-induced obesity accelerates the progression of spontaneous osteoarthritis in senescence-accelerated mouse prone 8. Mod. Rheumatol. *34*, 831–840.

61. Khachigian, L.M. (2006). Collagen antibody-induced arthritis. Nat. Protoc. *1*, 2512–2516.

62. Moore, A.R., Allden, S., Bourne, T., Denis, M.C., Kranidioti, K., Okoye, R., Sotsios, Y., Stencel, Z., Vugler, A., Watt, G., et al. (2014). Collagen II antibody-induced arthritis in Tg1278TNFko mice: optimization of a novel model to assess treatments targeting human TNFalpha in rheumatoid arthritis. J. Transl. Med. *12*, 285.

63. Crumeyrolle-Arias, M., Jaglin, M., Bruneau, A., Vancassel, S., Cardona, A., Daugé, V., Naudon, L., and Rabot, S. (2014). Absence of the gut microbiota enhances anxiety-like behavior and neuroendocrine response to acute stress in rats. Psychoneuroendocrinology *42*, 207–217.

64. Kato, Y., Nakasa, T., Sumii, J., Kanemitsu, M., Ishikawa, M., Miyaki, S., and Adachi, N. (2023). Changes in the Subchondral Bone Affect Pain in the Natural Course of Traumatic Articular Cartilage Defects. Cartilage *14*, 247–255.

65. Brenner, M., Meng, H.C., Yarlett, N.C., Griffiths, M.M., Remmers, E.F., Wilder, R.L., and Gulko, P.S. (2005). The non-major histocompatibility complex quantitative trait locus Cia10 contains a major arthritis gene and regulates disease severity, pannus formation, and joint damage. Arthritis Rheum. *52*, 322–332.

66. Shibuya, H., Nakasa, T., Adachi, N., Nagata, Y., Ishikawa, M., Deie, M., Suzuki, O., and Ochi, M. (2013). Overexpression of microRNA-223 in rheumatoid arthritis synovium controls osteoclast differentiation. Mod. Rheumatol. *23*, 674–685.

67. Saiz, A.M., Rahmati, M., Johnson, S.D., Bhat, A.S., Baldini, T.D., Øvrebø, Ø., Nogueira, L.P., Khassawna, T.E., Stötzel, S., Fierro, F.A., et al. (2025). Systemic versus local delivery of mesenchymal stem cells to improve the early stages of fracture healing in a polytrauma model. J. Biol. Eng. *19*, 82.

68. Tran, T.T.T., Phung, C.D., Yeo, B.Z.J., Prajogo, R.C., Jayasinghe, M.K., Yuan, J., Tan, D.S.W., Yeo, E.Y.M., Goh, B.C., Tam, W.L., et al. (2024). Customised design of antisense oligonucleotides targeting EGFR driver mutants for personalised treatment of non-small cell lung cancer. EBioMedicine *108*, 105356.

69. Kawamoto, T., and Shimizu, M. (2000). A method for preparing 2- to 50-micron-thick fresh-frozen sections of large samples and undecalcified hard tissues. Histochem. Cell Biol. *113*, 331–339.

70. Umezu, T., Takanashi, M., Murakami, Y., Ohno, S.I., Kanekura, K., Sudo, K., Nagamine, K., Takeuchi, S., Ochiya, T., and Kuroda, M. (2021). Acerola exosome-like nanovesicles to systemically deliver nucleic acid medicine via oral administration. Mol. Ther. Methods Clin. Dev. *21*, 199–208.

71. Mao, Q.Q., Xu, X.Y., Cao, S.Y., Gan, R.Y., Corke, H., Beta, T., and Li, H.B. (2019). Bioactive Compounds and Bioactivities of Ginger (Zingiber officinale Roscoe). Foods *8*, 185.

72. Wang, S., Zhang, C., Yang, G., and Yang, Y. (2014). Biological properties of 6-gingerol: a brief review. Nat. Prod. Commun. *9*, 1027–1030.

73. Zheng, Y., Rouillon, S., Khemakhem, M., Balakirouchenane, D., Lui, G., Abdalla, S., Sanoufi, M.R., Sauvaitre, L., Thebault, L., Hirt, D., et al. (2024). A rapid LC-MS/MS method for the simultaneous quantification of ivacaftor, lumacaftor, elexacaftor, tezacaftor, hexyl-methyl ivacaftor and ivacaftor carboxylate in human plasma. J. Pharm. Biomed. Anal. *248*, 116322.

74. Wang, Z., Gerstein, M., and Snyder, M. (2009). RNA-Seq: a revolutionary tool for transcriptomics. Nat. Rev. Genet. *10*, 57–63.

75. Love, M.I., Huber, W., and Anders, S. (2014). Moderated estimation of fold change and dispersion for RNA-seq data with DESeq2. Genome Biol. *15*, 550.

76. Raudvere, U., Kolberg, L., Kuzmin, I., Arak, T., Adler, P., Peterson, H., and Vilo, J. (2019). g:Profiler: a web server for functional enrichment analysis and conversions of gene lists (2019 update). Nucleic Acids Res. *47*, W191–W198.

77. Wu, T., Hu, E., Xu, S., Chen, M., Guo, P., Dai, Z., Feng, T., Zhou, L., Tang, W., Zhan, L., et al. (2021). clusterProfiler 4.0: A universal enrichment tool for interpreting omics data. Innovation *2*, 100141.

78. Miyaki, S., Sato, T., Inoue, A., Otsuki, S., Ito, Y., Yokoyama, S., Kato, Y., Takemoto, F., Nakasa, T., Yamashita, S., et al. (2010). MicroRNA-140 plays dual roles in both cartilage development and homeostasis. Genes Dev. *24*, 1173–1185.

79. Phinney, D.G., Di Giuseppe, M., Njah, J., Sala, E., Shiva, S., St Croix, C.M., Stolz, D.B., Watkins, S.C., Di, Y.P., Leikauf, G.D., et al. (2015). Mesenchymal stem cells use extracellular vesicles to outsource mitophagy and shuttle microRNAs. Nat. Commun. *6*, 8472.

80. Phinney, D.G., and Pittenger, M.F. (2017). Concise Review: MSC-Derived Exosomes for Cell-Free Therapy. Stem Cell. *35*, 851–858.

81. Than, U.T.T., Guanzon, D., Broadbent, J.A., Parker, T.J., and Leavesley, D.I. (2020). Deep Sequencing MicroRNAs from Extracellular Membrane Vesicles Revealed the Association of the Vesicle Cargo with Cellular Origin. Int. J. Mol. Sci. *21*, 1141.

82. Zhou, Y., Zhou, B., Pache, L., Chang, M., Khodabakhshi, A.H., Tanaseichuk, O., Benner, C., and Chanda, S.K. (2019). Metascape provides a biologist-oriented resource for the analysis of systems-level datasets. Nat. Commun. *10*, 1523.

83. Chen, X., Zhou, Y., and Yu, J. (2019). Exosome-like Nanoparticles from Ginger Rhizomes Inhibited NLRP3 Inflammasome Activation. Mol. Pharm. *16*, 2690–2699.

84. McHugh, M.L. (2011). Multiple comparison analysis testing in ANOVA. Biochem. Med. *21*, 203–209.

OMTN, Volume *37*

Supplemental information

Oral ginger-derived extracellular vesicles ameliorate arthritis via anti-inflammatory actions of microRNA-149 and 6-gingerol

Hiroki Kaneta, Tomoyuki Nakasa, Dilimulati Yimiti, Dan Moriwaki, Riku Kawasaki, Toshihiko Ogura, Shigeru Miyaki, and Nobuo Adachi

Supplemental Figures

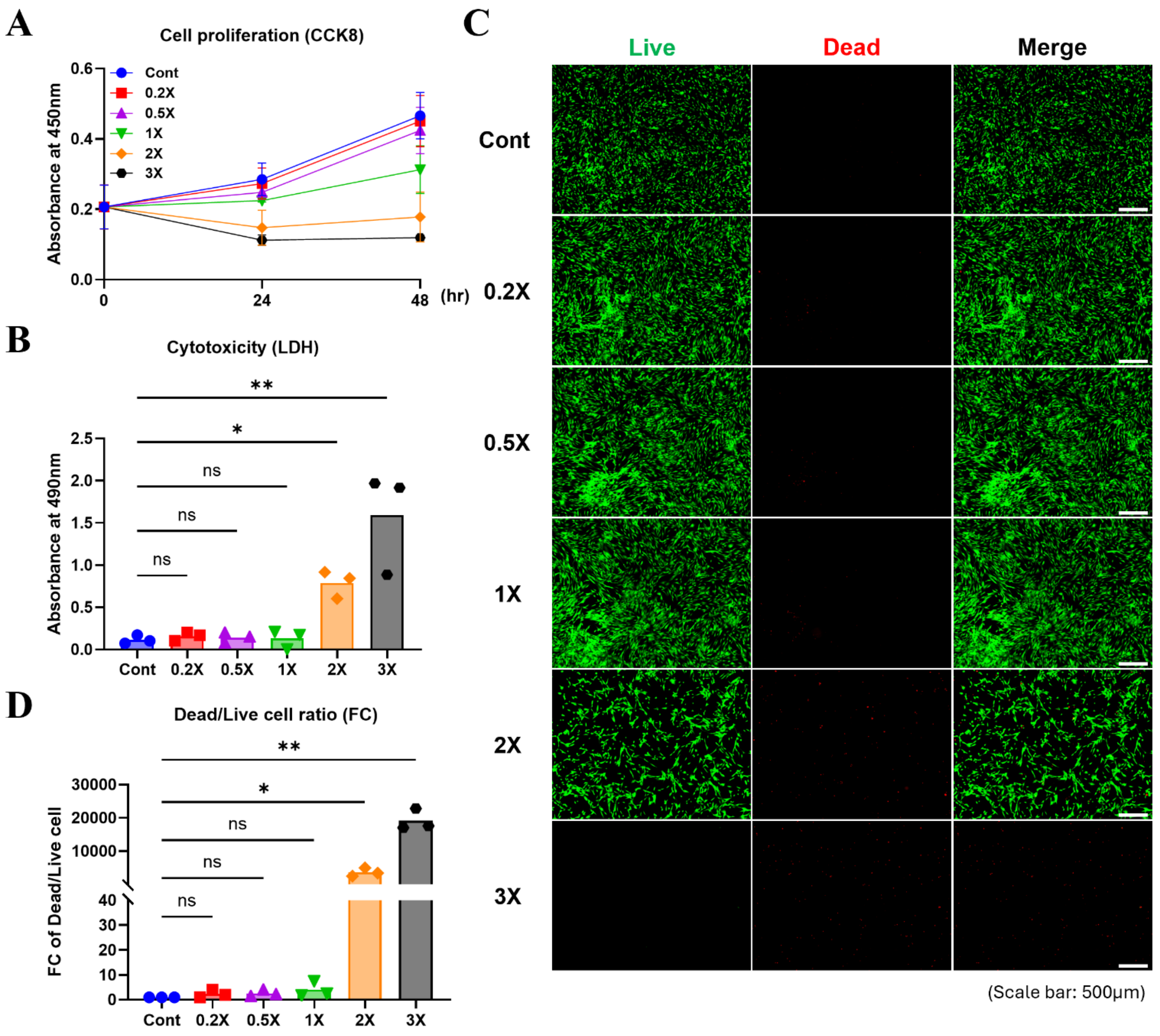

Figure S1. Dose-dependent effects of GDEVs on RASF proliferation and cytotoxicity. (A) Cell viability of RASF treated with different concentrations of GDEVs (0.2X, 0.5X, 1X, 2X, 3X) for 24 and 48 h, measured using the CCK-8 assay (n = 3 per group). Higher concentrations (2X and 3X) showed stronger inhibition of proliferation compared with 1X. (B) Cytotoxicity assessed by LDH release after 24 h of treatment with various concentrations of GDEVs (n = 3 per group). LDH release increased markedly at concentrations ≥ 2X, indicating cytotoxicity. (C, D) Live/Dead staining of RASF treated with different GDEV concentrations. In

the fluorescence images, live cells appear green and dead cells appear red. Representative fluorescence images (C) and quantification of live/dead cell ratios (D) are shown (n = 3 per group). GDEVs at 2X and 3X induced evident cell death, whereas 1X did not show cytotoxic effects. GDEV, ginger-derived extracellular vesicles; RASF, rheumatoid arthritis synovial fibroblasts; CCK-8, Cell Counting Kit-8; LDH, lactate dehydrogenase; FC, Fold Change; cont, control; ns, not significant. **, $p < 0.01$; *, $p < 0.05$.

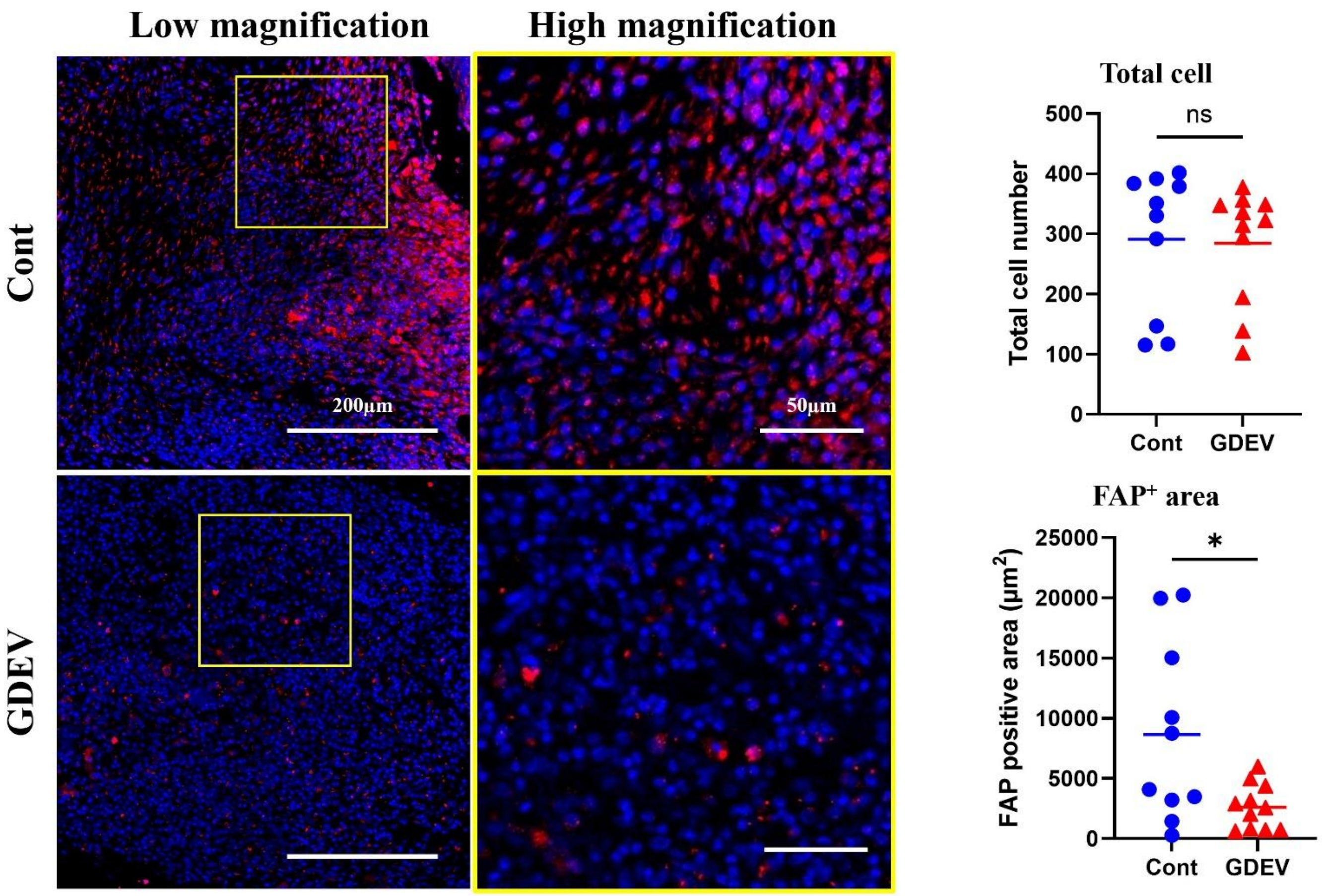

Figure S2. FAP immunofluorescence staining in CAIA mice. Representative images of synovial tissue stained with anti-FAPα antibody (red) and DAPI (blue) are shown at low (left) and high (right) magnification. Yellow squares in low-magnification images indicate regions of interest (200 μm × 200 μm). Quantification of total $DAPI^+$ nuclei and FAP-positive area are shown in the graphs. GDEV treatment did not significantly affect total $DAPI^+$ nuclei, but significantly reduced FAP-positive area compared with the control group (Cont: n = 10, GDEV: n = 11). FAP, fibroblast activation protein-α; CAIA, collagen antibody-induced arthritis; DAPI, 4′,6-diamidino-2-phenylindole; GDEV, ginger-derived extracellular vesicle; cont, control; ns, not significant. *, $p < 0.05$

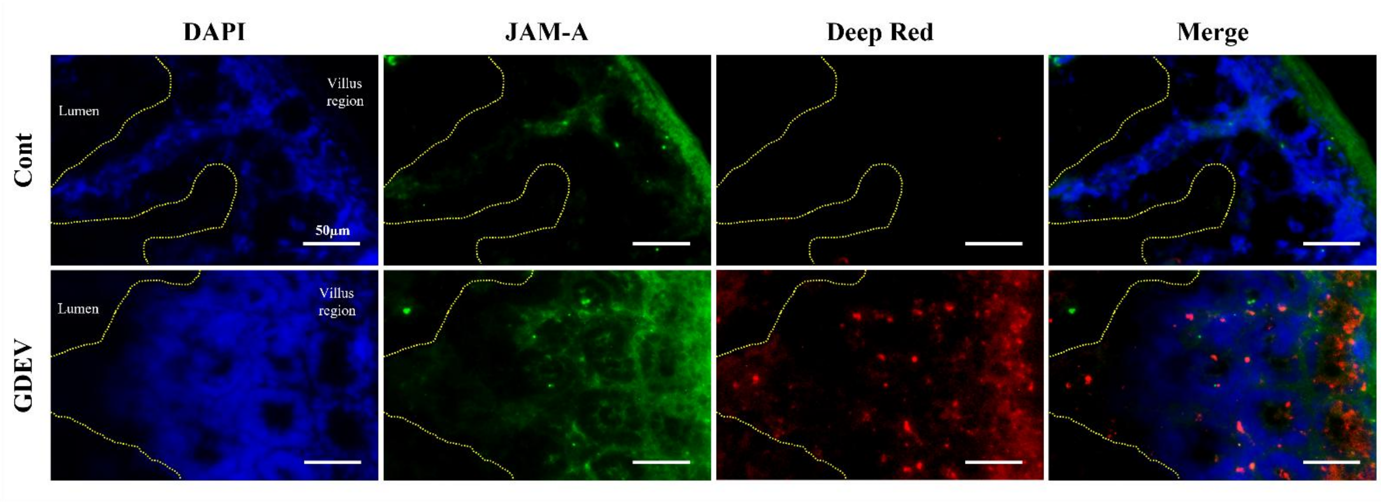

Figure S3. Uptake of GDEVs by intestinal epithelial cells (immunofluorescence for JAM-A).

Representative frozen sections of the small intestine obtained from the same experimental conditions shown in Figure 4, with additional immunofluorescence staining for JAM-A to identify intestinal epithelial cells. Nuclei were counterstained with DAPI (blue), GDEVs were labeled with Aco600 (red), and JAM-A was visualized in green. The luminal side is shown on the left, and the intestinal epithelial layer (villus region) is on the right. Merged images demonstrate the localization and internalization of Aco600-labeled GDEVs within JAM-A^{+} epithelial cells. GDEV, ginger-derived extracellular vesicles; DAPI, 4′ ,6-diamidino-2-phenylindole; Aco600, fluorescent dye used to label GDEVs; JAM-A, junctional adhesion molecule-A; cont, control.

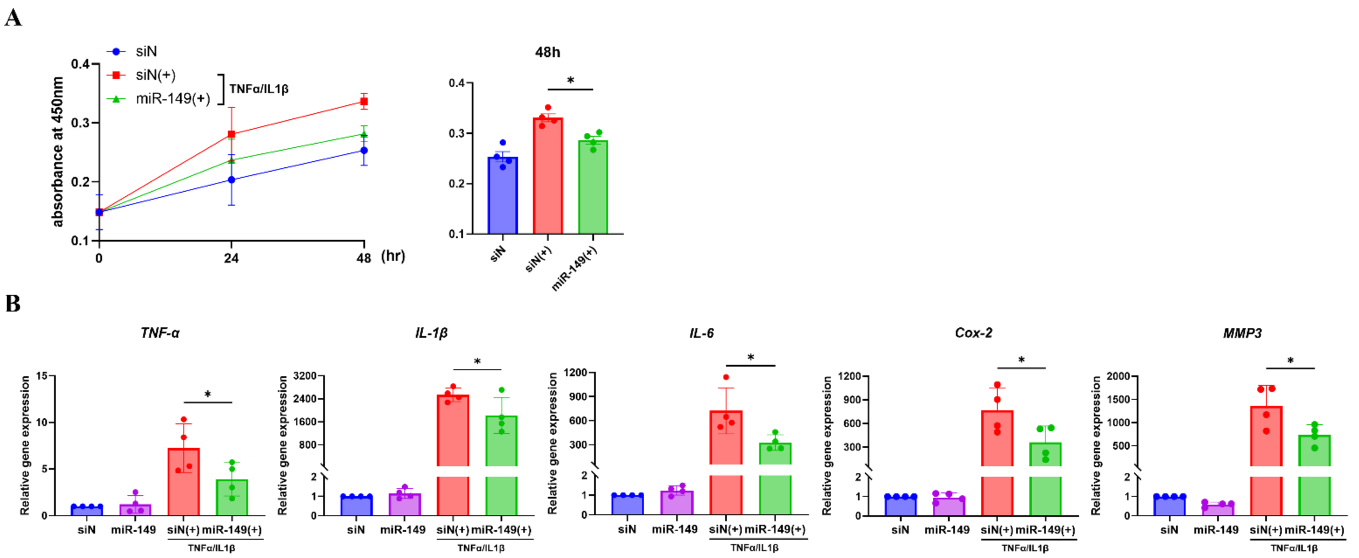

Figure S4. In vitro analysis of miR-149 effects on RASF. (A) Cell viability of RASF measured by MTT assay (n = 4 per group). Three groups were analyzed: SiN, SiN + TNFα/IL1β, TNFα/IL1β + miR-149. MiR-149 suppressed RASF proliferation under TNFα/IL1β stimulation. (B) Gene expression analysis of inflammatory mediators (TNFα, IL-1β, IL-6, COX-2, and MMP3) by qRT-PCR under the same experimental conditions (n = 4 per group). Four groups were analyzed: SiN, miR-149, SiN + TNFα/IL1β, and TNFα/IL1β + miR-149. MiR-149 significantly suppressed expression of these inflammatory genes compared with the inflammation-only group. RASF, rheumatoid arthritis synovial fibroblasts; MTT, 3-(4,5-dimethylthiazol-2-yl)-2,5-diphenyltetrazolium bromide; SiN, scrambled negative control; TNFα, tumor necrosis factor alpha; IL-1β, interleukin-1 beta; IL-6, interleukin-6; COX-2, cyclooxygenase-2; MMP3, matrix metalloproteinase-3; qRT-PCR, quantitative reverse-transcription polymerase chain reaction. *, $p < 0.05$

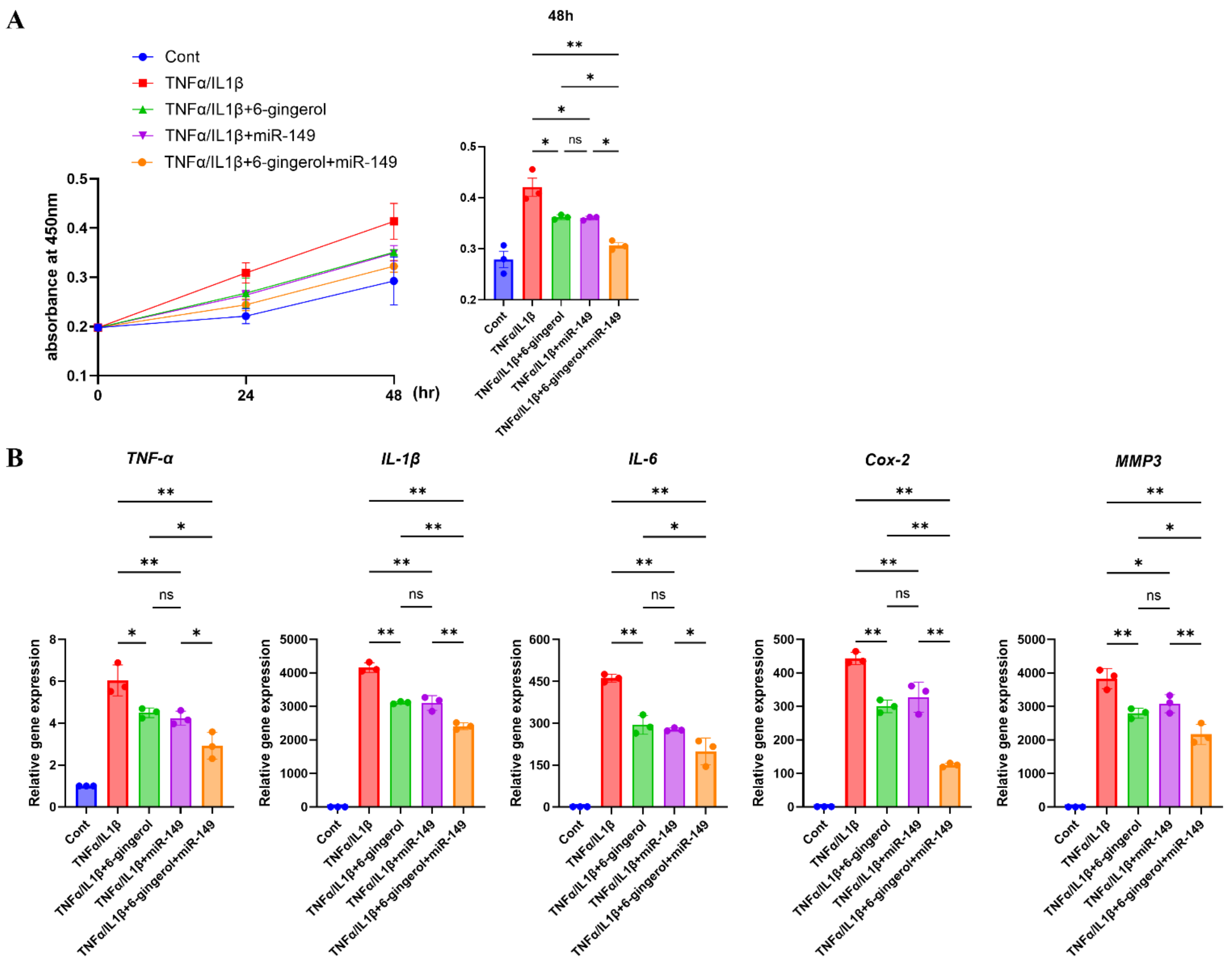

Figure S5. In vitro effects of combination treatment with miR-149 mimic and 6-gingerol on RASF. (A) RASFs were transfected with miR-149 mimic and subsequently treated with 6-gingerol. Cell viability was assessed using CCK-8 after 24 h (n = 3 per group). Compared with each single treatment, the combination of miR-149 mimic and 6-gingerol showed an enhanced suppressive effect on cell activation. (B) Gene expression analysis of inflammatory mediators was performed by qRT-PCR under the same conditions (n = 3 per group). The combination treatment resulted in greater suppression of inflammatory gene expression compared with either treatment alone. miR, microRNA; RASF, rheumatoid arthritis synovial fibroblasts; CCK-8, Cell Counting Kit-8; qRT-PCR, quantitative reverse-transcription polymerase chain reaction. **, $p < 0.01$; *, $p < 0.05$.

Supplemental Tables

Table S1: Representative plant-derived EV (PDEV) bioactive components and their therapeutic potential

**Disease / Condition**	**Source of PDEVs**	**Key miRNAs / Components**	**Mechanism / Therapeutic Potential**	**Reference**
Colitis	Ginger EVs	miR-7267-3p, bioactive lipids	Targets ycNE, increasing I3A and IL-22, leading to attenuation of colitis , and modulates gut microbiota	Suppl. Ref.1
Colitis	Ginger EVs	>125 miRNAs (unidentified individually)	Downregulates TNF-α, IL-6, IL-1β, and upregulates IL-10 and IL-22, providing protection against DSS-induced colitis	Suppl. Ref.2
Colitis	Ginger EVs	27 highly expressed miRNAs	Inhibits NF-κB, IL-6, IL-8, and TNF-α; uptake occurs via caveolin-mediated endocytosis and micropinocytosis	16
Intestinal barrier / inflammation	Broccoli EVs	Plant miRNAs, sulforaphane-containing lipids	Activates AMPK in dendritic cells, inducing tolerogenic DCs and improving DSS colitis	Suppl. Ref.3
Cancer (chemo-sensitization)	Grapefruit EVs	miR-17	Modulates MHC-I expression, enhancing chemo-sensitivity	Suppl. Ref.4
Rheumatoid arthritis	Milk EVs	miR-30a, miR-223, miR-92a	Downregulates IL-6, MCP-1, and IgG2a, ameliorating cartilage and bone inflammation	Suppl. Ref.5
Liver injury (hepatoprotection)	Ginger EVs	miRNAs + 6-gingerol / 6-shogaol	Reduces ALT and AST, decreases TG, and protects against alcohol-induced liver injury	Suppl. Ref.6

EV, Extracellular vesicle; miRNA/miR, microRNA; ycNE, yeast cell–derived nanovesicles; I3A, indole-3-aldehyde; IL, interleukin; DSS, dextran sulfate sodium; NF-κB, nuclear factor kappa-light-chain-enhancer of activated B cells; TNF-α, Tumor necrosis factor-alpha; AMPK, AMP-activated protein kinase; DCs, dendritic cells; MHC-I, major histocompatibility complex class I; MCP-1, monocyte chemoattractant protein-1; IgG2a, immunoglobulin G2a; ALT, alanine aminotransferase; AST, aspartate aminotransferase; TG, triglyceride; Suppl. Ref, Supplemental References

Table S2: Characterization of Ginger EVs

Parameter		ginger EV
Particle concentration	(particles/mL)	2.66×10^9
Median diameter	(nm)	239
Protein concentration	(mg/mL)	3.8

EV, Extracellular vesicle

Table S3: Highly Expressed miRNAs in Ginger EVs (Microarray Analysis)

No.	miRNA	Expression value	Mechanism / Target	Therapeutic Effect / Disease	Reference
1	hsa-miR-6087	15942.4	Regulates CCNA2, RFC3/4, BLM in hESC cardiomyogenesis via polysome association	Controls cell cycle during cardiomyocyte differentiation; tissue regeneration	Suppl. Ref. 7
2	hsa-miR-6088	14876.4	Regulated in glioma cells via ceRNA effect of lncRNA MEG3, affecting SMARCB1 expression	Tumor-suppressive role in glioma	Suppl. Ref. 8
3	hsa-miR-149-3p	4776.4	Inhibits HIPK2-mediated RASF proliferation, migration, invasion; modulates circ_0008410 axis	Anti-inflammatory; suppresses RASF dysfunction	34
			Inhibits NF-κB activation; downregulates TNF-α, IL-6, IL-1β	Anti-inflammatory; suppresses inflammation in RA	40
			Enhances AMPK, inhibits NF-κB; downregulated in IBD	Anti-inflammatory; ameliorates colitis; potential biomarker for IBD severity	Suppl. Ref. 9,10
4	hsa-miR-6800-5p	4485.4	Highly expressed in patient serum; biomarker with high diagnostic accuracy	Potential biomarker for ovarian cancer	Suppl. Ref. 11
5	hsa-miR-6085	4238.4	Not defined	Not defined	
6	hsa-miR-6089	3429.4	Targets TLR4, downregulating IL-6, IL-29, TNF-α in LPS-stimulated macrophages	Anti-inflammatory; potential biomarker/therapeutic target in rheumatoid arthritis	Suppl. Ref. 12
7	hsa-miR-3665	2825.4	Downregulated by promoter hypermethylation; MAPK/RAS pathway	Promoter hypermethylation linked to poor prognosis in esophageal squamous cell carcinoma	Suppl. Ref. 13
8	hsa-miR-6763-5p	2558.4	Significantly downregulated in plasma exosomes of epithelial ovarian cancer (EOC) patients	Potential non-invasive diagnostic biomarker for EOC	Suppl. Ref. 14
9	hsa-miR-4281	2345.4	Upregulated in fulminant myocarditis; inflammation-related	Potential circulating biomarker for fulminant myocarditis	Suppl. Ref. 15

10	hsa-miR-3188	2205.4	Targets mTOR, suppressing PI3K/AKT signaling	Tumor-suppressive; inhibits cell proliferation in non-small cell lung cancer	Suppl. Ref. 16
11	hsa-miR-3960	2006.4	Targets PHLDA2; inhibits IL-1β-mediated inflammation in chondrocytes	Anti-inflammatory; protects cartilage in osteoarthritis	Suppl. Ref. 17
12	hsa-miR-8072	1692.4	Not defined	Not defined	
13	hsa-miR-937-5p	1649.4	Targets IL-1β; suppresses TNF-α/IL-17 signaling in airway epithelial cells	Anti-inflammatory; protects airway epithelial cells post-CSE damage in COPD	Suppl. Ref. 18
14	hsa-miR-6511b-5p	1517.4	Upregulated in serum exosomes of RA patients in remission	Potential biomarker for RA disease activity	Suppl. Ref. 19
15	hsa-miR-5787	1401.4	Targets TLR4/NF-κB signaling in macrophages	Anti-inflammatory; inhibits macrophage proliferation and migration (cerebral infarction model)	Suppl. Ref. 20
16	hsa-miR-6090	1310.4	Not defined	Not defined	
17	hsa-miR-642a-3p	1257.4	Targets SERPINE1; promotes EMT, migration, and invasion	Tumor progression in hepatocellular carcinoma	Suppl. Ref. 21
			Protects β cells against glucolipotoxicity	β cell protection in diabetes	Suppl. Ref. 22
18	hsa-miR-4529-3p	1109.4	Targets RB1; activates ERK1/2 pathway	Promotes progression of retinoblastoma	Suppl. Ref. 23
19	hsa-miR-150-3p	1014.4	Targets Trim14; modulates NF-κB and IFN-β signaling	Suppresses cartilage degradation; protective effect in osteoarthritis	Suppl. Ref. 24

EV, Extracellular vesicle; hsa, Homo sapiens (human); miRNA/miR, microRNA; CCNA2, Cyclin A2; RFC3/4, Replication factor C subunit 3/4; BLM, Bloom syndrome RecQ-like helicase; hESC, Human embryonic stem cell; ceRNA, Competing endogenous RNA; lncRNA, Long non-coding RNA; MEG3, Maternally expressed gene 3; SMARCB1, SWI/SNF-related, matrix-associated, actin-dependent regulator of chromatin subfamily B member 1; HIPK2, Homeodomain-interacting protein kinase 2; RASF, Rheumatoid arthritis synovial fibroblasts; NF-κB, Nuclear factor kappa-light-chain-enhancer of activated B cells; TNF-α, Tumor necrosis factor-alpha; IL, Interleukin; AMPK, AMP-activated protein kinase; IBD, Inflammatory bowel disease; TLR4, Toll-like receptor 4; LPS, Lipopolysaccharide; MAPK, Mitogen-activated protein kinase; RAS, Rat sarcoma (proto-oncogene family); EOC, Epithelial ovarian cancer; mTOR, Mechanistic target of rapamycin; PI3K, Phosphoinositide 3-kinase; AKT, Protein kinase B; PHLDA2, Pleckstrin homology-like domain, family A, member 2; CSE, Cigarette smoke extract; COPD, Chronic obstructive pulmonary disease; SERPINE1, Serpin family E member 1 (plasminogen activator inhibitor-1, PAI-1); EMT, Epithelial-mesenchymal transition; RB1, Retinoblastoma protein; ERK1/2, Extracellular signal-regulated kinase 1/2; Trim14, Tripartite motif-containing protein 14; IFN-β, Interferon-beta; Suppl. Ref, Supplemental References

Table S4: Target Genes Based on Across-Study Analysis

Pathway	Target Genes	Functional relevance	Reference
Ras signaling	FLT4	Lymphangiogenesis, immune cell trafficking	[41]
	PLA2G2A	Eicosanoid production, pro-inflammatory mediator	[42]
	KSR1	MAPK scaffold, regulates ERK activity	[43]
	TTBK1	Neuroinflammation, immune modulation	[Suppl. Ref. 25]
MAPK cascade	IGFBP4	IGF signaling, cytokine regulation	[Suppl. Ref. 26]
	SOX9	Cartilage homeostasis, ECM maintenance	[Suppl. Ref. 27]
	DUSP19	MAPK phosphatase, fine-tunes inflammation	[44]

Ras, Rat sarcoma virus oncogene; MAPK, mitogen-activated protein kinase; FLT4, Fms related receptor tyrosine kinase 4; PLA2G2A, Phospholipase A2 group IIA; KSR1, Kinase suppressor of Ras 1; TTBK1, Tau tubulin kinase 1; IGFBP4, Insulin-like growth factor binding protein 4; SOX9, SRY-box transcription factor 9; DUSP19, Dual specificity phosphatase 19; ERK, extracellular signal–regulated kinase; ECM, extracellular matrix; Suppl. Ref, Supplemental References

Table S5: Ginger-derived Bioactive Components and Reported Therapeutic Effects

Compound	Reported therapeutic effects	References
6-Gingerol	Anti-inflammatory: Inhibits synovitis; reduces MMPs, TNF-α, IL-6; suppresses 5-LOX and prostaglandin synthase activity	[21]
	Anti-osteoclastogenic: Inhibits inflammation-associated osteoclast differentiation via reduction of PGE_2 levels	[23]
6-Shogaol	Anti-inflammatory: Inhibits synovitis; reduces proliferation and apoptosis of synovial tissue via PI3K/Akt/NF-κB signaling; effective in collagen-induced and carrageenan-induced arthritis models	[22, 25]
	Cartilage-protective: Inhibits chondrocyte innate immune responses and cathepsin-K activity, thereby reducing cartilage degradation.	[24]
8-Shogaol	Anti-inflammatory: Inhibits synovitis; suppresses TNF-α, IL-1β, IL-17-mediated inflammation and migration; improves arthritis in vivo (AIA rat model)	[19]
Zingerone	Anti-inflammatory (inhibits NF-κB/MAPK, reduces TNF-α, IL-1β, IL-6); Antioxidant (scavenges free radicals, prevents lipid peroxidation)	[Suppl. Ref. 28]
6-Paradol	Anti-inflammatory; inhibits proliferation and metastasis via EGFR/PI3K/AKT; reduces cytokines IL-6, TNF-α; antioxidant	[Suppl. Ref. 29]

MMP, matrix metalloproteinase; TNF-α, tumor necrosis factor-alpha; IL-6, interleukin-6; 5-LOX, 5-lipoxygenase; PGE_2, prostaglandin E_2; PI3K, phosphatidylinositol 3-kinase; Akt, protein kinase B; NF-κB, nuclear factor-kappa B; IL-1β, interleukin-1 beta; IL-17, interleukin-17; AIA, adjuvant-induced arthritis; MAPK, mitogen-activated protein kinase; EGFR, Epidermal Growth Factor Receptor; Suppl. Ref, Supplemental References

Table S6: Rheumatoid arthritis patient profiles

No.	Age (years)	Sex	BMI (kg/m^2)	Larsen grade *	Medicine	RF (IU/mL)	CRP (mg/dL)	ESR (mm/h)	Duration of disease (years)	Surgical treatment
1	57	F	18.6	3	Methotrexate, Bucillamine, Salazosulfapyridine, Prednisolone	21.5	0.96	37	7	TAA
2	73	F	23.5	4	Celecoxib, Salazosulfapyridine, Prednisolone, Tocilizumab	45.8	1.15	46	5	TAA
3	75	F	17.6	4	Methotrexate, Prednisolone, Etanercept	127.7	3.30	122	7	TKA
4	65	M	15.3	4	Loxoprofen, Methotrexate, Golimumab	6.7	4.11	78	15	TKA
5	58	M	24.7	3	Celecoxib, Prednisolone, Etanercept	170.5	0.67	9	27	THA

BMI, Body Mass Index; RF, Rheumatoid Factor; CRP, C-Reactive Protein; ESR, Erythrocyte Sedimentation Rate; TAA, Total Ankle Arthroplasty; TKA, Total Knee Arthroplasty; THA, Total Hip Arthroplasty; *, Supplemental reference [30]

Table S7. TaqMan Gene Expression Assay IDs for qRT-PCR

Gene	Target Name	Assay ID
TNF-α	Tumor necrosis factor-alpha	Hs00174128_m1
IL-1β	Interleukin-1 beta	Hs01555410_m1
IL-6	Interleukin-6	Hs00985639_m1
Cox-2	Cyclooxygenase-2	Hs00153133_m1
MMP3	Matrix metalloproteinase 3	Hs00233962_m1
GAPDH	Glyceraldehyde-3-phosphate dehydrogenase	Hs99999905_m1

qRT-PCR, quantitative reverse-transcription polymerase chain reaction.

Table S8. List of common miRNAs between MSCEVs and GDEVs

This table provides the list of 2,307 miRNAs commonly detected in MSCEVs and GDEVs by microarray analysis.

Due to the large dataset, this table is provided as a separate Excel file.

Supplemental Materials and Methods

Cell Proliferation Assay

To evaluate the appropriate concentration range of ginger-derived extracellular vesicles (GDEVs), Rheumatoid arthritis synovial fibroblasts (RASFs) were seeded in 96-well plates at a density of 3×10^3 cells/well in Dulbecco's modified Eagle medium (DMEM; high glucose, without L-glutamine and phenol red; FUJIFILM Wako Pure Chemical Corporation, Osaka, Japan) supplemented with 10% fetal bovine serum (FBS; Thermo Fisher Scientific, Waltham, MA, USA) and 1% penicillin–streptomycin–amphotericin B (AB; FUJIFILM Wako Pure Chemical Corporation, Osaka, Japan).

After overnight incubation, the following treatment groups were prepared: non-treated control and GDEVs at various concentrations (0.2X, 0.5X, 1X, 2X, 3X). After 24 h of treatment, CCK-8 reagent (Dojindo Laboratories, Kumamoto, Japan) was added to each well and incubated for 1 h.

Absorbance was measured at 450 nm using a microplate reader (Tecan Infinite 200 PRO, Tecan Group Ltd., Männedorf, Switzerland) at 0, 24, and 48 h to evaluate cell proliferation. This procedure was performed following the same methodology described in the Main Methods section.

Cytotoxicity Assay (LDH Release and Live/Dead Cell Staining)

Cytotoxicity induced by GDEVs was evaluated using a Lactate Dehydrogenase (LDH) release assay. RASFs were seeded in 24-well plates at 5×10^4 cells/well and treated with various concentrations of GDEVs for 24 h.

Culture supernatants (100 μL) were collected and mixed with reaction reagent from the Cytotoxicity LDH Assay Kit-WST (Dojindo Laboratories, Kumamoto, Japan) according to the manufacturer's protocol. After a 30-min incubation at room temperature in the dark, absorbance was measured at 490 nm.[31]

Live and dead cells were assessed using the Cellstain Double Staining Kit (Dojindo Laboratories, Kumamoto, Japan). After washing with phosphate-buffered saline (PBS), cells were incubated with calcein-AM (live cell indicator) and propidium iodide (dead cell indicator) diluted in PBS for 15 min at 37 °C.[32]

Fluorescent images were acquired using a fluorescence microscope (BZ-X710, Keyence Corporation, Osaka, Japan), and live/dead cells were counted using Keyence analysis software.

FAP immunofluorescence staining and quantification

Fibroblast activation protein-α (FAPα) is highly expressed in synovial fibroblasts of patients with rheumatoid arthritis (RA), where it has been implicated in synovial inflammation and joint destruction.[33] Based on this disease relevance, FAP expression was evaluated in our experimental model by immunofluorescence staining. Paraffin sections of EDTX-decalcified mouse joint tissue were deparaffinized and treated with Proteinase K (DAKO, S3020, Glostrup, Denmark) for antigen retrieval. After PBS washes, sections were blocked with 5% normal goat serum (Invitrogen, MA, USA), 1% BSA (Wako, Osaka, Japan), and 0.1% Triton X-100 (Sigma-Aldrich, MO, USA) in PBS. Sections were incubated overnight at 4 °C with the primary antibody against fibroblast activation protein-α (FAPα; Cloud-Clone, PAC469Mu01, TX, USA) or rabbit IgG isotype control (Invitrogen, MA, USA). After rinsing with PBS, sections were treated with ImmunoClear (Matsunami, Osaka, Japan), followed by incubation with Alexa Fluor 568-conjugated anti-rabbit IgG secondary antibody (Invitrogen, A-11011, MA, USA; 1:500) and DAPI (Dojindo Laboratories Co., Ltd., Kumamoto, Japan; 1:1000) for 1 h at room temperature. Finally, sections were treated with ImmunoClear, washed with PBS, and mounted with antifade medium (Fluoromount, Cosmo Bio, Tokyo, Japan).

Fluorescence images were acquired using a KEYENCE BZ-X710 fluorescence microscope (KEYENCE, Osaka, Japan) under identical acquisition settings. For each sample, five random regions of interest (ROIs; 0.04 mm^2 each) were selected within the synovial tissue, excluding non-synovial tissues such as bone and muscle using the KEYENCE BZ-X Analyzer. Quantitative analysis was performed using the KEYENCE BZ-X Analyzer, measuring total DAPI-stained nuclei and FAP-positive area. The mean values of the five ROIs per sample were used for statistical analysis.

Immunofluorescence staining of JAM-A to identify intestinal epithelial cells

Junctional adhesion molecule-A (JAM-A) is a tight junction protein specifically expressed in intestinal epithelial cells, and thus was used as a marker to identify epithelial regions.[34] Cryosections of mouse intestinal tissue were air-dried and rinsed in PBS. Sections were blocked with 5% normal goat serum (Invitrogen, MA, USA), 1% BSA (Wako, Osaka, Japan), and 0.1% Triton X-100 (Sigma-Aldrich, MO, USA) in PBS for 30 min at 37 °C. The primary antibody against JAM-A (Proteintech, 16183-1-AP, IL, USA; rabbit polyclonal, 1:100 dilution in blocking buffer) was applied overnight at 4 °C, following a previously described method.[35] The following day, sections were washed twice with ImmunoClear (Matsunami, Osaka, Japan) and three times with PBS. Secondary antibody (Alexa Fluor 488–conjugated donkey anti-rabbit IgG (H+L); Invitrogen, A32790, MA, USA; 1:500 dilution in blocking buffer) and DAPI (Dojindo Laboratories Co., Ltd., Kumamoto, Japan; 1:1000) were applied for 1 h at 37 °C. Finally, sections were washed with PBS and mounted with antifade medium (Fluoromount, Cosmo Bio, Tokyo, Japan).

Transfection of miR-149-3p mimic into RASF

Double-stranded RNA oligonucleotides representing the mature miR-149-3p sequence (sense: 5′-UCUGGCUCCGUGUCUUCACUCCC-3′ and antisense: 5′-GGGAGUGAAGACACGGAGCCAGA-3′) were synthesized by Hokkaido System Science Co., Ltd. (Sapporo, Japan). A final concentration of 10 nM miR-149-3p mimic or a negative control RNA (Silencer Negative Control siRNA #1, Thermo Fisher Scientific, Waltham, MA, USA) was transfected into rheumatoid arthritis synovial fibroblasts (RASF) using Lipofectamine RNAiMAX reagent (Thermo Fisher Scientific, Waltham, MA, USA) according to the manufacturer's instructions.[36] The concentration of 10 nM was selected based on our preliminary experiments showing stable transfection efficiency and minimal cytotoxicity at 1, 5, and 10 nM, as well as previously reported studies employing similar concentrations for miR-149-3p transfection in cultured cells.[37] After 24 h of incubation, transfected cells were used for the subsequent analyses.

Combination Treatment of miR-149 and 6-Gingerol

To evaluate the combinatory effects of miR-149 and 6-gingerol, RASFs were assigned to three groups: (1) miR-149 only, (2) 6-gingerol only, and (3) miR-149 + 6-gingerol (combined treatment). miR-149 mimic transfection and 6-gingerol administration were performed as described in the main Methods. After treatment under inflammatory stimulation (TNFα 5 ng/mL and IL-1β 5 ng/mL), cell proliferation was assessed by CCK-8 assay, and inflammatory gene expression (TNFα, IL-1β, IL-6, COX-2, MMP3) was analyzed by qRT-PCR following standard procedures described above. This procedure was performed following the same methodology described in the Main Methods section.

Supplemental References

1. Teng, Y., Ren, Y., Sayed, M., Hu, X., Lei, C., Kumar, A., Hutchins, E., Mu, J., Deng, Z., Luo, C., et al. (2018). Plant-Derived Exosomal MicroRNAs Shape the Gut Microbiota. Cell Host Microbe *24*, 637-652 e638.
2. Zhang, M., Viennois, E., Prasad, M., Zhang, Y., Wang, L., Zhang, Z., Han, M.K., Xiao, B., Xu, C., Srinivasan, S., et al. (2016). Edible ginger-derived nanoparticles: A novel therapeutic approach for the prevention and treatment of inflammatory bowel disease and colitis-associated cancer. Biomaterials *101*, 321-340.
3. Deng, Z., Rong, Y., Teng, Y., Mu, J., Zhuang, X., Tseng, M., Samykutty, A., Zhang, L., Yan, J., Miller, D., et al. (2017). Broccoli-Derived Nanoparticle Inhibits Mouse Colitis by Activating Dendritic Cell AMP-Activated Protein Kinase. Mol Ther *25*, 1641-1654.
4. Zhuang, X., Teng, Y., Samykutty, A., Mu, J., Deng, Z., Zhang, L., Cao, P., Rong, Y., Yan, J., Miller, D., et al. (2016). Grapefruit-derived Nanovectors Delivering Therapeutic miR17 Through an Intranasal Route Inhibit Brain Tumor Progression. Mol Ther *24*, 96-105.
5. Arntz, O.J., Pieters, B.C., Oliveira, M.C., Broeren, M.G., Bennink, M.B., de Vries, M., van Lent, P.L., Koenders, M.I., van den Berg, W.B., van der Kraan, P.M., et al. (2015). Oral administration of bovine milk derived extracellular vesicles attenuates arthritis in two mouse models. Mol Nutr Food Res *59*, 1701-1712.
6. Zhuang, X., Deng, Z.B., Mu, J., Zhang, L., Yan, J., Miller, D., Feng, W., McClain, C.J., and Zhang, H.G. (2015). Ginger-derived nanoparticles protect against alcohol-induced liver damage. J Extracell Vesicles *4*, 28713.
7. Machado, H.C., Bispo, S., and Dallagiovanna, B. (2023). miR-6087 Might Regulate Cell Cycle-Related mRNAs During Cardiomyogenesis of hESCs. Bioinform Biol Insights *17*, 11779322231161918.
8. Gong, X. and Huang, M.Y. (2020). Tumor-Suppressive Function of lncRNA-MEG3 in Glioma Cells by Regulating miR-6088/SMARCB1 Axis. Biomed Res Int *2020*, 4309161.
9. Feng, Q., Li, Y., Zhang, H., Wang, Z., Nie, X., Yao, D., Han, L., Chen, W.D., and Wang, Y.D. (2022). Deficiency of miRNA-149-3p shaped gut microbiota and enhanced dextran sulfate sodium-induced colitis. Mol Ther Nucleic Acids *30*, 208-225.
10. Luo, S. and Chen, X.H. (2024). Tissue and serum miR-149-3p/5p in hospitalized patients with inflammatory bowel disease: Correlation with disease severity and inflammatory markers. Kaohsiung J Med Sci *40*, 131-138.
11. Hamidi, F., Gilani, N., Arabi Belaghi, R., Yaghoobi, H., Babaei, E., Sarbakhsh, P., and Malakouti, J. (2023). Identifying potential circulating miRNA biomarkers for the diagnosis and prediction of ovarian cancer using machine-learning approach: application of Boruta. Front Digit Health *5*, 1187578.
12. Xu, D., Song, M., Chai, C., Wang, J., Jin, C., Wang, X., Cheng, M., and Yan, S. (2019). Exosome-encapsulated miR-6089 regulates inflammatory response via targeting TLR4. J Cell Physiol *234*, 1502-1511.
13. Zhou, J., Liu, S., Zhang, J., Zeng, Q., Lin, Z., Fu, R., Lin, Y., and Hu, Z. (2025). Discovery and validation of Hsa-microRNA-3665 promoter methylation as a potential biomarker for the prognosis of esophageal squaous cell carcinoma. Int J Clin Oncol *30*, 309-319.
14. Wang, S., Song, X., Wang, K., Zheng, B., Lin, Q., Yu, M., Xie, L., Chen, L., and Song, X. (2022). Plasma

exosomal miR-320d, miR-4479, and miR-6763-5p as diagnostic biomarkers in epithelial ovarian cancer. Front Oncol *12*, 986343.

15. Nie, X., He, M., Wang, J., Chen, P., Wang, F., Lai, J., Li, C., Yu, T., Zuo, H., Cui, G., et al. (2020). Circulating miR-4763-3p Is a Novel Potential Biomarker Candidate for Human Adult Fulminant Myocarditis. Mol Ther Methods Clin Dev *17*, 1079-1087.
16. Wang, C., Liu, E., Li, W., Cui, J., and Li, T. (2018). MiR-3188 Inhibits Non-small Cell Lung Cancer Cell Proliferation Through FOXO1-Mediated mTOR-p-PI3K/AKT-c-JUN Signaling Pathway. Front Pharmacol *9*, 1362.
17. Ye, P., Mi, Z., Wei, D., Gao, P., Ma, M., and Yang, H. (2022). miR-3960 from Mesenchymal Stem Cell-Derived Extracellular Vesicles Inactivates SDC1/Wnt/beta-Catenin Axis to Relieve Chondrocyte Injury in Osteoarthritis by Targeting PHLDA2. Stem Cells Int *2022*, 9455152.
18. Liu, T. (2021). miR-937 serves as an inflammatory inhibitor in cigarette smoke extract-induced human bronchial epithelial cells by targeting IL1B and regulating TNF-alpha/IL-17 signaling pathway. Tob Induc Dis *19*, 55.
19. Lim, M.K., Yoo, J., Sheen, D.H., Ihm, C., Lee, S.K., and Kim, S.A. (2020). Serum Exosomal miRNA-1915-3p Is Correlated With Disease Activity of Korean Rheumatoid Arthritis. In Vivo *34*, 2941-2945.
20. Bao, Z., Zhang, S., and Li, X. (2021). MiR-5787 Attenuates Macrophages-Mediated Inflammation by Targeting TLR4/NF-kappaB in Ischemic Cerebral Infarction. Neuromolecular Med *23*, 363-370.
21. Zhang, S., Cao, G., Shen, S., Wu, Y., Tan, X., and Jiang, X. (2024). CAF-derived miR-642a-3p supports migration, invasion, and EMT of hepatocellular carcinoma cells by targeting SERPINE1. PeerJ *12*, e18428.
22. Pinhancos, S.S., Teixeira de Oliveira, J., Alves, C.H., Deus, C.M., de Winter, T.J.J., Viana, S., Reis, F., Santos, J., Buitinga, M., Carlotti, F., et al. (2025). miRNA-642a-3p protects beta cells from glucolipotoxicity. Mol Ther Nucleic Acids *36*, 102498.
23. Gao, Y. and Du, P. (2024). miR-4529-3p Promotes the Progression of Retinoblastoma by Inhibiting RB1 Expression and Activating the ERK Signaling Pathway. Mol Biotechnol *66*, 102-111.
24. Wang, H., Shu, J., Zhang, C., Wang, Y., Shi, R., Yang, F., and Tang, X. (2022). Extracellular Vesicle-Mediated miR-150-3p Delivery in Joint Homeostasis: A Potential Treatment for Osteoarthritis? Cells *11*.
25. Dillon, G.M., Henderson, J.L., Bao, C., Joyce, J.A., Calhoun, M., Amaral, B., King, K.W., Bajrami, B., and Rabah, D. (2020). Acute inhibition of the CNS-specific kinase TTBK1 significantly lowers tau phosphorylation at several disease relevant sites. PLoS One *15*, e0228771.
26. Bayati, P., Taherian, M., and Mojtabavi, N. (2024). Immunomodulatory effects of the induced pluripotent stem cells through expressing IGF-related factors and IL-10 in vitro. Int J Immunopathol Pharmacol *38*, 3946320241276899.
27. Haseeb, A., Kc, R., Angelozzi, M., de Charleroy, C., Rux, D., Tower, R.J., Yao, L., Pellegrino da Silva, R., Pacifici, M., Qin, L., et al. (2021). SOX9 keeps growth plates and articular cartilage healthy by inhibiting chondrocyte dedifferentiation/osteoblastic redifferentiation. Proc Natl Acad Sci U S A *118*.
28. Ahmad, B., Rehman, M.U., Amin, I., Arif, A., Rasool, S., Bhat, S.A., Afzal, I., Hussain, I., Bilal, S., and Mir, M. (2015). A Review on Pharmacological Properties of Zingerone (4-(4-Hydroxy-3-methoxyphenyl)-2-butanone).

ScientificWorldJournal *2015*, 816364.
29. Jiang, X., Wang, J., Chen, P., He, Z., Xu, J., Chen, Y., Liu, X., and Jiang, J. (2021). [6]-Paradol suppresses proliferation and metastases of pancreatic cancer by decreasing EGFR and inactivating PI3K/AKT signaling. Cancer Cell Int *21*, 420.
30. Larsen, A., Dale, K., and Eek, M. (1977). Radiographic evaluation of rheumatoid arthritis and related conditions by standard reference films. Acta Radiol Diagn (Stockh) *18*, 481-491.
31. Takeda, Y. and Dai, P. (2022). Capsaicin directly promotes adipocyte browning in the chemical compound-induced brown adipocytes converted from human dermal fibroblasts. Sci Rep *12*, 6612.
32. Zhu, L., Yang, J., Zhang, J., and Peng, B. (2014). A comparative study of BioAggregate and ProRoot MTA on adhesion, migration, and attachment of human dental pulp cells. J Endod *40*, 1118-1123.
33. Bauer, S., Jendro, M.C., Wadle, A., Kleber, S., Stenner, F., Dinser, R., Reich, A., Faccin, E., Godde, S., Dinges, H., et al. (2006). Fibroblast activation protein is expressed by rheumatoid myofibroblast-like synoviocytes. Arthritis Res Ther *8*, R171.
34. Wong, E.A. and Kinstler, S.R. (2023). Research Note: Junctional adhesion molecule A is expressed in epithelial cells of the crypt and villi whereas junctional adhesion molecule 2 is expressed in vascular cells. Poult Sci *102*, 102693.
35. Li, Y., Peng, L., Cao, X., Yang, K., Wang, Z., Xiao, Y., Xiao, H., Qian, C., and Liu, H. (2022). The Long Non-Coding RNA HOXC-AS3 Promotes Glioma Progression by Sponging miR-216 to Regulate F11R Expression. Front Oncol *12*, 845009.
36. Sumimoto, Y., Harada, Y., Yimiti, D., Watanabe, C., Miyaki, S., and Adachi, N. (2024). MicroRNA-26a deficiency attenuates the severity of frozen shoulder in a mouse immobilization model. J Orthop Res *42*, 2623-2633.
37. Jin, L., Li, Y., Liu, J., Yang, S., Gui, Y., Mao, X., Nie, G., and Lai, Y. (2016). Tumor suppressor miR-149-5p is associated with cellular migration, proliferation and apoptosis in renal cell carcinoma. Mol Med Rep *13*, 5386-5392.
